# Endogenous *Coriobacteriaceae* enriched by a high-fat diet promotes colorectal tumorigenesis through the CPT1A-ERK axis

**DOI:** 10.1038/s41522-023-00472-7

**Published:** 2024-01-20

**Authors:** Qiulin Tang, Huixi Huang, Huanji Xu, Hongwei Xia, Chenliang Zhang, Di Ye, Feng Bi

**Affiliations:** Division of Abdominal Cancer, Department of Medical Oncology, Cancer Center and Laboratory of Molecular Targeted Therapy in Oncology, West China Hospital, Sichuan University, Chengdu, Sichuan Province 610041 China

**Keywords:** Clinical microbiology, Microbiota

## Abstract

A high-fat diet (HFD) may be linked to an increased colorectal cancer (CRC) risk. Stem cell proliferation and adipokine release under inflammatory and obese conditions are the main factors regulating CRC progression. Furthermore, alterations in intestinal flora have been linked to tumorigenesis and tumour progression. However, whether a HFD can promote CRC occurrence by altering intestinal flora remains unclear. The objective of this study was to identify bacterial strains enriched by a HFD and investigate the association and mechanism by which a HFD and bacterial enrichment promote CRC occurrence and development. In this study, the intestinal microbiota of mice was assessed using 16S rRNA and metagenomic sequencing. Serum metabolites of HFD-fed mice were assessed using tandem liquid chromatography-mass spectrometry. CRC cell lines and organoids were co-cultured with *Coriobacteriaceae* to evaluate the effect of these bacteria on the CPT1A-ERK signalling pathway. We found that *Coriobacteriaceae* were enriched in the colons of HFD-fed mice. An endogenous *Coriobacteriaceae* strain, designated as *Cori*.ST1911, was successfully isolated and cultured from the stools of HFD-fed mice, and the tumorigenic potential of *Cori*.ST1911 in CRC was validated in several CRC mouse models. Furthermore, *Cori*.ST1911 increased acylcarnitine levels by activating CPT1A, demonstrating the involvement of the CPT1A-ERK axis. We also found that the endogenous *Lactobacillus* strain *La.mu*730 can interfere with *Cori*.ST1911 colonisation and restore gut barrier function. In conclusion, we identified a novel endogenous intestinal *Coriobacteriaceae*, *Cori*.ST1911, which might lead to a new gut microbiota intervention strategy for the prevention and treatment of CRC.

## Introduction

Colorectal cancer (CRC) is the second leading cause of cancer-related mortality worldwide, and its prevalence is increasing, particularly among people under 50 years of age^1,2^. Intestinal microbiome homeostasis plays a role in the maintenance of host physiological functions and disease occurrence by interfacing with the immune system, and diseases such as metabolic syndrome, obesity-related diseases, liver disease, inflammatory bowel disease, and colorectal cancer may be caused by gut microbiome disruption^3^. Specifically, the relative abundance of putative cancer-promoting microbiota, including *Fusobacterium nucleatum*, pks^+^ *Escherichia coli*, *Bacteroides fragilis*, *Enterococcus faecalis*, *Streptococcus gallolyticus*, and *Peptostreptococcus* are greater in CRC tumour tissues^4–6^.

A high-fat diet (HFD) significantly alters intestinal flora composition, increasing the *Firmicutes/Bacteroidetes* ratio and changing the abundance of specific bacterial groups^7^, as well as increasing the proportion of opportunistic pathogens in the colons of C57BL/6J mice. Numerous studies have indicated a HFD as an independent risk factor in tumorigenesis due to intestinal microflora disruption^8^. Independent of obesity, a HFD can disrupt intestinal microflora and increase tumorigenesis. However, there is no concrete evidence that a HFD induces pathogenic intestinal microflora to induce colon cancer^9^.

*Coriobacteriaceae*, a bacterial family belonging to the order Coriobacteriales, phylum Actinobacteria, were previously assumed to be normal inhabitants the mammalian oral cavity, gastrointestinal tract, and reproductive system^10^. However, these bacteria are associated with various diseases, such as bacteraemia, periodontitis, and vaginosis, and may therefore also be considered as pathogens. Furthermore, *Coriobacteriaceae* have recently gained attention in studies of colon cancer and sequencing of 16S rRNA genes classified as actinomycetes (including *Collinsella spp*.) have indicated increased prevalence of *Collinsella, Eggerthella, Olsenella*, and *Slackia*, genera belonging to the family *Coriobacteriaceae* in the stools of patients with CRC^11,12^.

In this study, we analysed the gut microbiota of mice fed a HFD. We isolated and enriched relevant strains, and identified the bacteria using metagenomic sequencing. Colon bacterial colonisation was investigated in C57BL/6J AOM and APC^Min/+^ mouse models, and the mechanism by which microbial metabolites promote CRC development was investigated using metabolomics, as well as in vitro and in vivo experiments. We found that *Coriobacteriaceae* were enriched in the intestinal microbiota of mice fed a HFD, promoting the development of colon cancer. This was evidenced by intestinal barrier destruction, causing acylcarnitine accumulation in response to upregulated carnitine palmityl transferase 1A (CPT1A) expression and tumorigenesis promoted via p-ERK activation. Moreover, we found that *Lactobacillus murinus* strain *La.mu*730 can inhibit *Coriobacteriaceae* colonisation, effectively reversing their carcinogenic effect.

## Results

### Influence of a HFD on *Coriobacteriaceae* in the mouse intestine

**Fig. 1:**
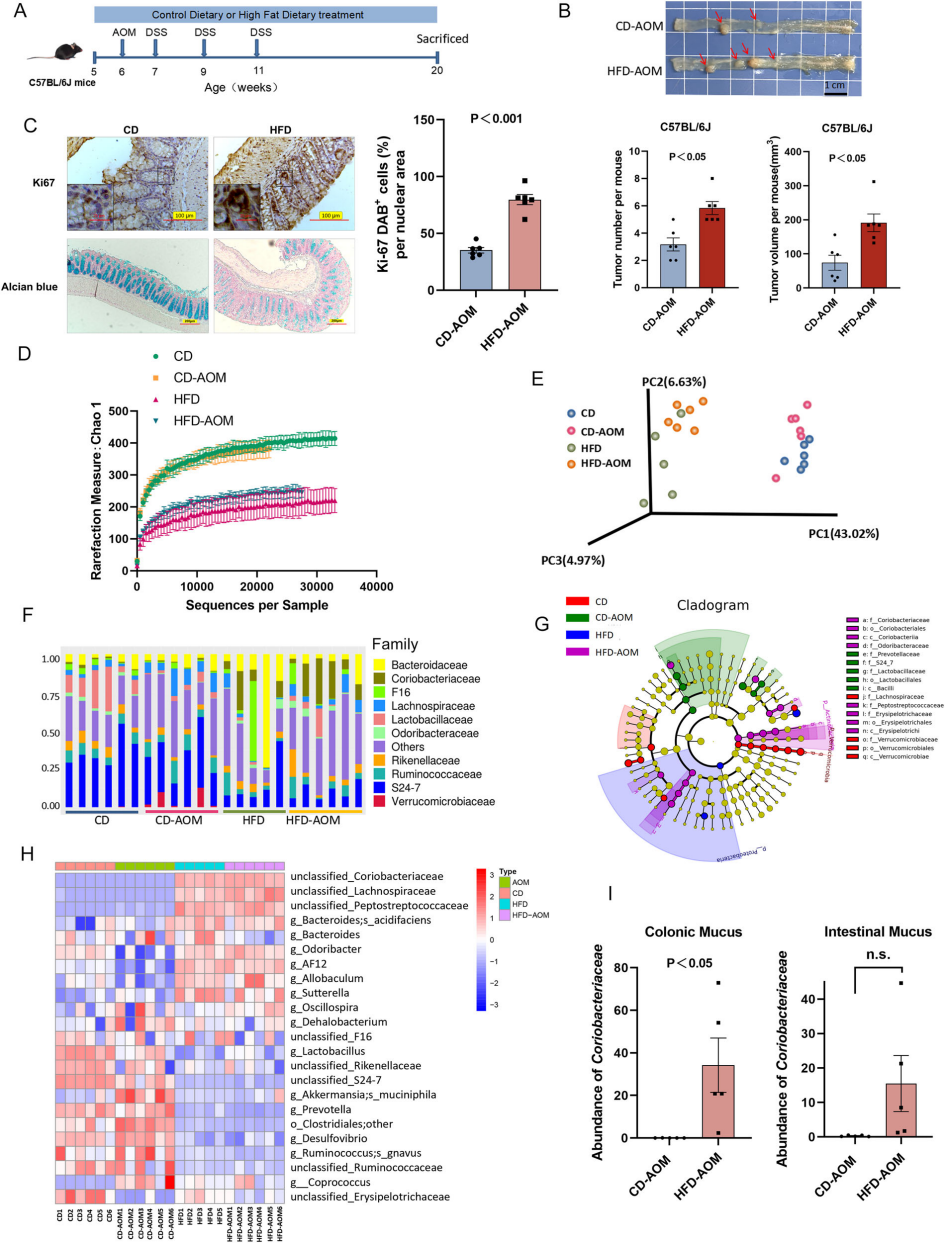
A high-fat diet is related to intestinal microbiota disruption and promotes colon cancer development in C57 mice. **A** Schematic diagram of the experimental procedure of AOM-DSS treatment of CD- and HFD-fed C57BL/6J mice. Six-week-old mice received a single intraperitoneal (i.p.) AOM (10 mg/kg) injection and three cycles of 1.5% DSS drinking water to establish the mouse primary colon cancer model. The mice were simultaneously fed a HFD (*n* = 6) or normal CD (*n* = 6) for 14 weeks. **B** Representative image of colon tumours in CD- and HFD-fed C57BL/6J AOM mice. **C**. Alcian blue staining (×10 magnification) and Ki67 expression (×20 magnification) in CD- and HFD-fed C57BL/6J AOM mice. Scale bars, 200 μm. **D**–**H** 16S rRNA sequencing of faecal microbiota of CD (*n* = 6), CD-AOM (*n* = 6), HFD (*n* = 5), and HFD-AOM (*n* = 6) mice at 20 weeks. **D** Colonic microbiota α-diversity curve (Chao1) of CD- and HFD-fed C57BL/6J AOM mice analysed with the Kruskal–Wallis test. **E** Unifrac PCoA of microbiota in CD- and HFD-fed C57BL/6J AOM mice. **F** Distribution of intestinal microbiota in each sample (grouping) of CD- and HFD-fed C57BL/6J AOM mice. **G** Cladogram of evolutionary branches of each level among CD- and HFD-fed C57BL/6J AOM mice using LEfSe analysis. The vertical axis represents the relative abundance of different species, the horizontal axis represents grouping information. **H** OTU heatmap in CD- and HFD-fed C57BL/6J AOM mice. The counts were log-transformed and used to define the heatmap colour gradient. **I** *Coriobacteriaceae* abundance in the small intestinal (left) and colonic (right) mucus of CD- and HFD-fed C57BL/6J AOM mice determined using qPCR, normalised to universal bacterial primers by targeting 16S rRNA genes (*n* = 5). Data are represented as mean ± SEM, analysed using the two-tailed Student’s t-test. AOM, azoxymethane; DSS, dextran sodium sulfate; CD, control diet; HFD, high-fat diet; OTU, operational taxonomic unit; LEfSe, linear discriminant analysis effect size; PCoA, principal coordinate analysis.

The harmful effects of a HFD on CRC were verified in C57/BL6J AOM mice, a specific-pathogen free (SPF) primary colon cancer mouse model induced using (AOM-DSS) (Fig. 1A). A HFD considerably increased the incidence of colon tumours (Fig. 1B, C) compared with a conventional control diet (CD). Immunohistochemical analysis revealed an increased Ki67 proliferation index in the colon tissue (Fig. 1C), and Alcian blue staining revealed a reduced number of goblet cells and reduced mucus secretion in mice fed a HFD, which suggests compromised intestinal barrier integrity (Fig. 1C).

16 S rRNA sequencing, which was performed to determine the influence of a HFD on the intestinal flora of C57/BL6J AOM and control mice, revealed significantly reduced intestinal bacterial diversity in HFD-fed mice compared with that in CD-fed mice, irrespective of whether AOM-DSS treatment was used (Fig. 1D). Principal component analysis (PCoA) based on the Bray-Curtis distance revealed substantial variations in principal components (PCs) between the HFD and CD groups along PC1, indicating a HFD as the primary factor influencing microbial composition (Fig. 1E). Operational taxonomic unit (OTU) species composition analysis revealed significantly lower *S24-7* and *Lactobacillus* levels in HFD mice compared with CD mice (Fig. 1F). Only *Coriobacteriaceae* were strongly and significantly represented in HFD mice, showing 23 taxa and the greatest abundance difference (Fig. 1F–H, Supplementary Fig. 1).

During tumour progression, changes in mucus layer composition affect microbial colonisation and intestinal permeability, leading to intestinal leakage and tumour development. *Coriobacteriaceae* abundance in the intestinal and colonic mucus of HFD C57/BL6J AOM mice was therefore measured. QPCR indicated that *Coriobacteriaceae* mainly colonise the colonic mucus rather than small intestinal mucus (Fig. 1I), indicating that *Coriobacteriaceae* enrichment may be associated with CRC progression.

### Isolation and identification of *Coriobacteriaceae*

**Fig. 2:**
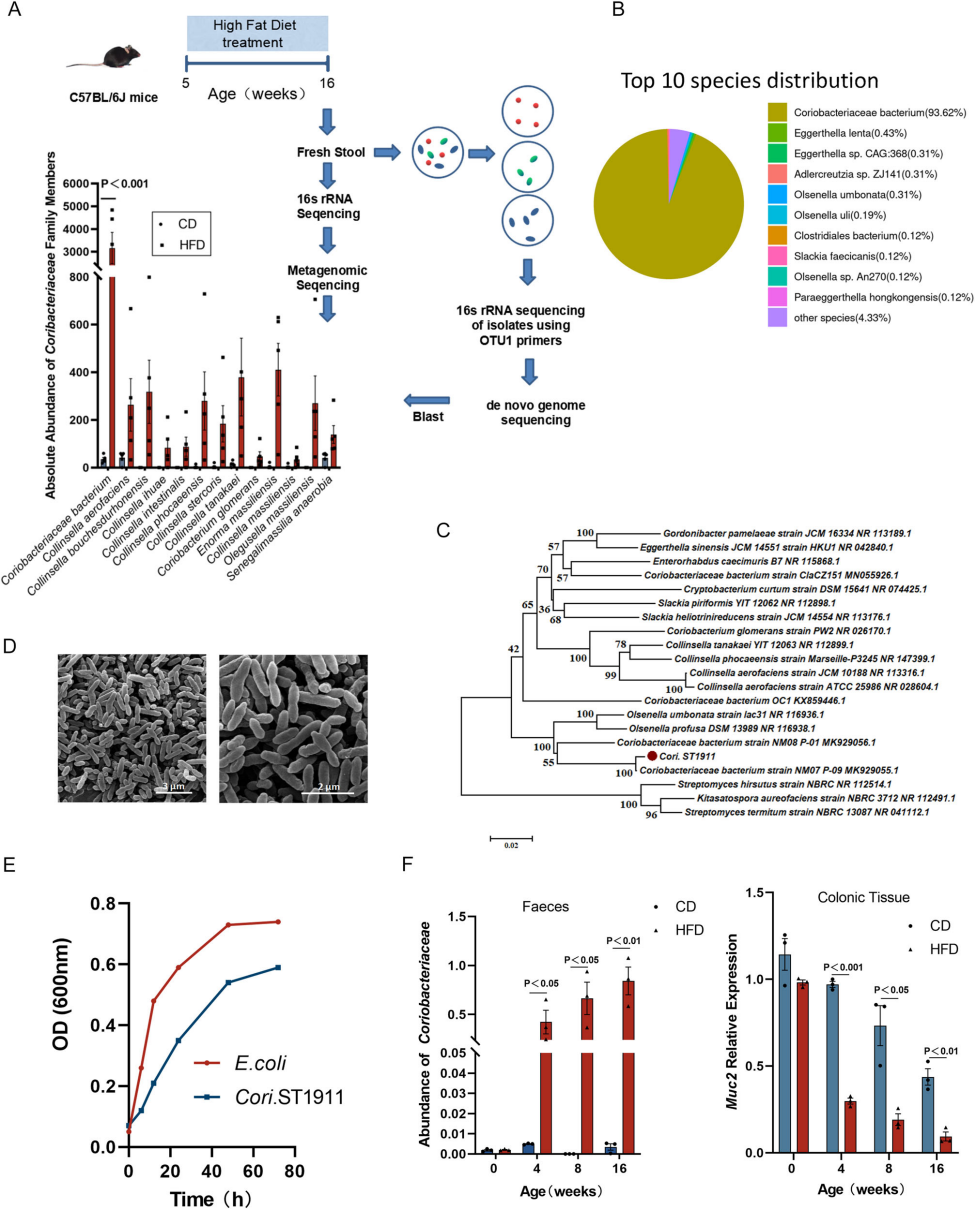
Endogenous *Coriobacteriaceae* strain *Cori*.ST1911 isolated from the stool of HFD-fed mice was related to the enrichment of unclassified *Coriobacteriaceae*. **A** Schematic diagram of the experimental procedure for *Cori*.ST1911 isolation and 16S rRNA and macrogenomic sequencing analysis to confirm its dominant position in the *Coriobacteriaceae family*. Data are represented as mean ± SEM, analysed using the two-tailed Student’s t-test. **B** *Cori*.ST1911 genome top 10 species distribution from the NR database. **C** Framing phylogeny evolutionary tree of the *Coriobacteriaceae* family and the *Cori*.ST1911 isolate. **D** Electron microscopic image showing typical morphological characteristics of *Cori*.ST1911. Scale bars, 3 µm (left), 2 µm (right). **E** Growth curves of *Cori*.ST1911. *E. coli* was used as a control. **F** *Coriobacteriaceae* abundance in stool (left) and Muc2 expression in mucus (right) of CD- and HFD-fed C57BL/6J AOM mice at 0, 4, and 8 weeks determined using qPCR. Data are represented as mean ± SEM, analysed using the two-tailed Student’s t-test. NR, non-redundant; OD, optical density.

To further explore the correlation between *Coriobacteriaceae*, a HFD, and CRC occurrence, we isolated an endogenous *Coriobacteriaceae* strain from the fresh stool of HFD-fed mice at 20 weeks and designated it *Cori*.ST1911 through the “sequence-guided separation” scheme^13^. Using the metagenome of faecal bacteria from HFD-fed mice, *Coriobacteriaceae bacterium* was identified as the primary member of the *Coriobacteriaceae* species influenced by a HFD, representing the most abundant OTU (OTU1) in *Coriobacteriaceae* (Fig. 2A), and exhibiting 100% similarity to the 16S rRNA gene sequence of the OTU1 representative sequence. De novo sequencing of the *Cori*.ST1911 genome indicated a whole genome consisting of a single circular 2,024,667 bp-long chromosome with a 58.49% G + C content (BioProject accession: PRJNA850882). Distribution analysis of the top ten species identified *Cori*.ST1911 as a likely member of the *Coriobacteriaceae* family (Fig. 2B). Phylogeny and its evolutionary relationship with other *Coriobacteriaceae* strains was then determined using the obtained 16S rRNA gene sequences (Fig. 2C). Comparison of the complete genome sequence of *Cori*.ST1911 and publicly available genome sequences of its phylogenetic relatives using average nucleotide identity (ANI) calculations with the species delimitation boundary set at 95–96%^14^, revealed that, *Cori*.ST1911 and *Coriobacteriaceae bacterium* strain NM-08 share ANI values of 96.14% to 98.06%, confirming that *Cori*.ST1911 can be classified as a species belonging to the family *Coriobacteriaceae*.

*Cori*.ST1911 cells are approximately 0.3–0.4 µm × 0.8–2 µm in size and typically appear as short rods or fusiform cells without flagella or other appendages (Fig. 2D). *Cori*.ST1911 reached a stationary phase after ~48 h incubation under anaerobic conditions (Fig. 2E). We extracted intestine and colon mucus from CD - and HFD fed C57BL/6J AOM mice, and quantified *Coriobacteriaceae* through qPCR. The results showed that the enrichment of *Coriobacteriaceae* originated from the colon rather than the small intestine, suggesting that its enrichment may be related to the progression of colon cancer (Fig. 2F). The Kirby-Bauer (K-B) antibiotic resistance sensitivity test for five antibiotics, including neomycin, metronidazole, streptomycin, ampicillin, and vancomycin, indicated that *Cori*.ST1911 has the highest sensitivity to metronidazole (Supplementary Table 1).

### Colonisation of *Cori*.ST1911 promotes the development and progression of CRC

**Fig. 3:**
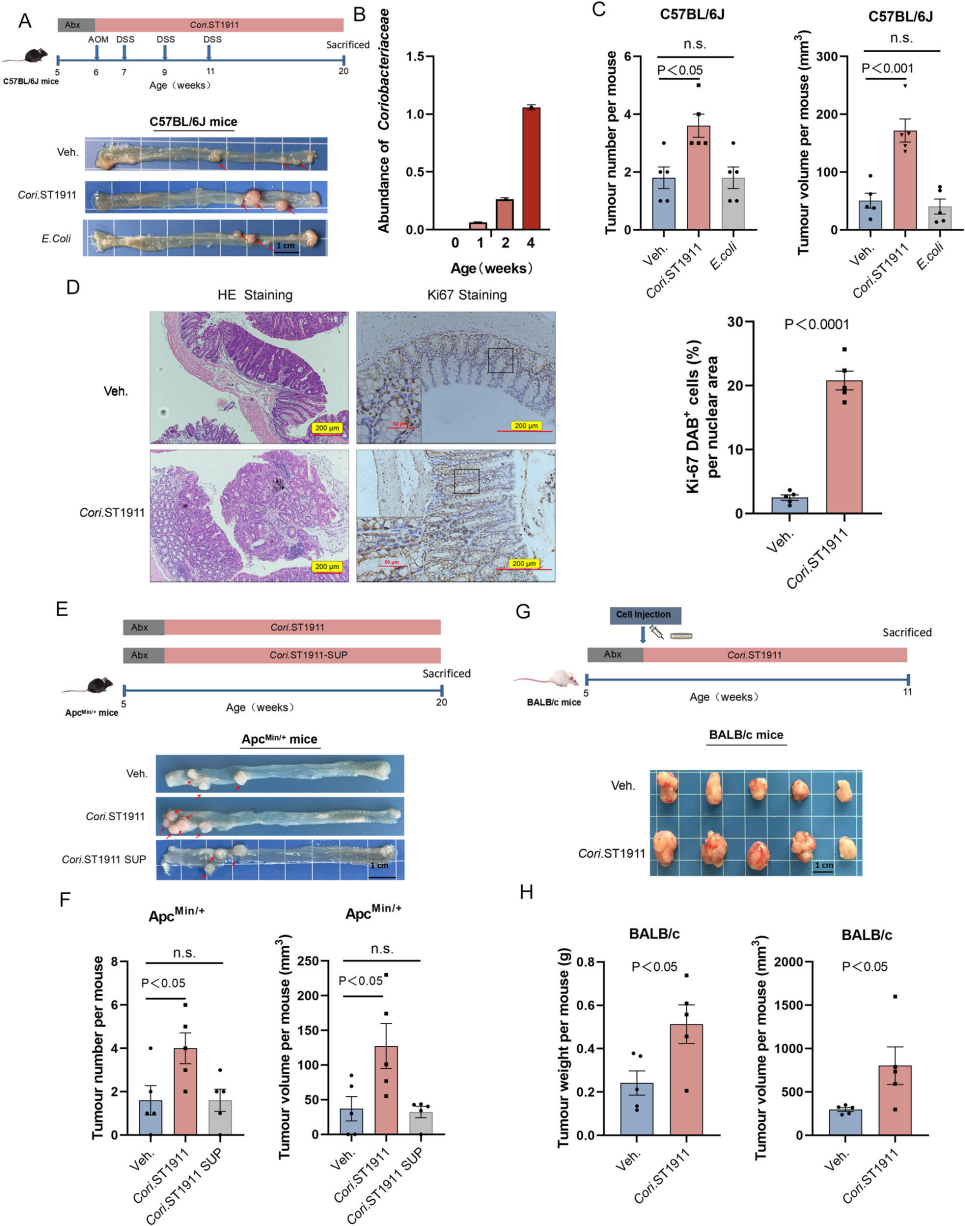
*Cori*.ST1911 promotes colon cancer tumorigenesis and development in different mouse models. **A** Schematic diagram of the experimental procedure for constructing C57BL/6J AOM mouse models and representative colon tumour images. **B** *Cori*.ST1911 abundance at 0, 1, 2, and 4 weeks determined using qPCR and normalised to universal bacterial primers by targeting 16S rRNA genes. **C** Tumour number and volume in C57BL/6J AOM mice (*n* = 5) gavaged with *Cori*.ST1911, *E. coli*, or vehicle. Data are represented as mean ± SEM, analysed using one-way ANOVA. n.s., no significance. **D** Representative images of HE and nuclear Ki67 staining of the *Cori*.ST1911 group and GAM broth control group mice. Scale bars, 200 µm. Percentage of nuclear Ki67^+^ cells (*n* = 5). Data are represented as mean ± SEM, analysed using two-tailed Student’s t-test. **E** Schematic diagram of the experimental procedure for constructing model Apc^Min/+^ mice and representative colon tumour images. **F** Tumour number and volume in Apc^Min/+^ mice (*n* = 5) gavaged with *Cori*.ST1911, *Cori*.ST1911 culture supernatant, or vehicle. Data are represented as mean ± SEM, analysed using one-way ANOVA. n.s., no significance. **G** Schematic diagram of the experimental procedure for constructing a subcutaneous transplanted tumour model in BALB/c mice and representative colon tumour images. Mice inoculated with CT26 cells were randomly assigned to *Cori*.ST1911 and GAM broth control groups when the tumour reached 3–4 mm. Tumour tissue was harvested 5 weeks later. **H** Tumour weight and volume in BALB/c mice gavaged with *Cori*.ST1911 or vehicle. Data are represented as mean ± SEM, analysed using two-tailed Student’s t-test. SUP, supernatant.

*Cori*.ST1911 enrichment induced by a HFD prompted further exploration of whether *Cori*.ST1911 is involved in CRC development in mouse models. The Apc^Min/+^ mouse model, which is widely used to simulate the natural state of CRC, was used as the primary tumour model, whereas the C57/BL6J AOM model was used to simulate inflammation-associated primary colon cancer. Therefore, these two models simulate the primary state of colon cancer via different mechanisms. Briefly, C57/BL6J AOM and Apc^Min/+^ mice were treated with an antibiotic cocktail followed by gavage with *Cori*.ST1911 every 2 days (Fig. 3A, E). Quantitative *Coriobacteriaceae* detection confirmed *Cori*.ST1911 colonisation in the C57/BL6J AOM mouse intestine (Fig. 3B). Both C57/BL6J AOM and Apc^Min/+^ mice colonised with *Cori*.ST1911 exhibited significantly higher colon tumour diversity (Fig. 3A, C; Fig. 3E, F) and a greater total colon tumour volume (Fig. 3C, F) than mice administered GAM broth, confirming that *Cori*.ST1911 promoted CRC development in both C57/BL6J AOM and Apc^Min/+^ mice. Hematoxylin-eosin (HE) and Ki67^+^ cell staining revealed that *Cori*.ST1911 treatment increased tumour cell proliferation in C57/BL6J AOM mouse colon tissue (Fig. 3D). Notably, Apc^Min/+^ mice treated with *Cori*.ST1911 culture supernatants did not exhibit a significant increase in CRC occurrence compared with the control group, indicating that *Cori*.ST1911 does not directly promote tumour formation via cellular metabolites (Fig. 3E, F).

We hypothesised that *Cori*.ST1911 promotes CRC growth by affecting host metabolism. *Cori*.ST1911 treatment of a BALB/c mouse model of subcutaneous transplantation tumours (Fig. 3G) was associated with significantly increased tumour weight and volume (Fig. 3H), which confirms that *Cori*.ST1911 can promote tumour growth in xenograft mouse models without direct contact with tumour cells. These data suggest that *Cori*.ST1911, but not its metabolic products, promotes CRC occurrence and progression, implying that *Cori*.ST1911 promotes tumour growth by interacting with intestinal cells and inducing host metabolic disorders.

### Influence of *Coriobacteriaceae* on tumour cell proliferation and the CPT1A-ERK axis

**Fig. 4:**
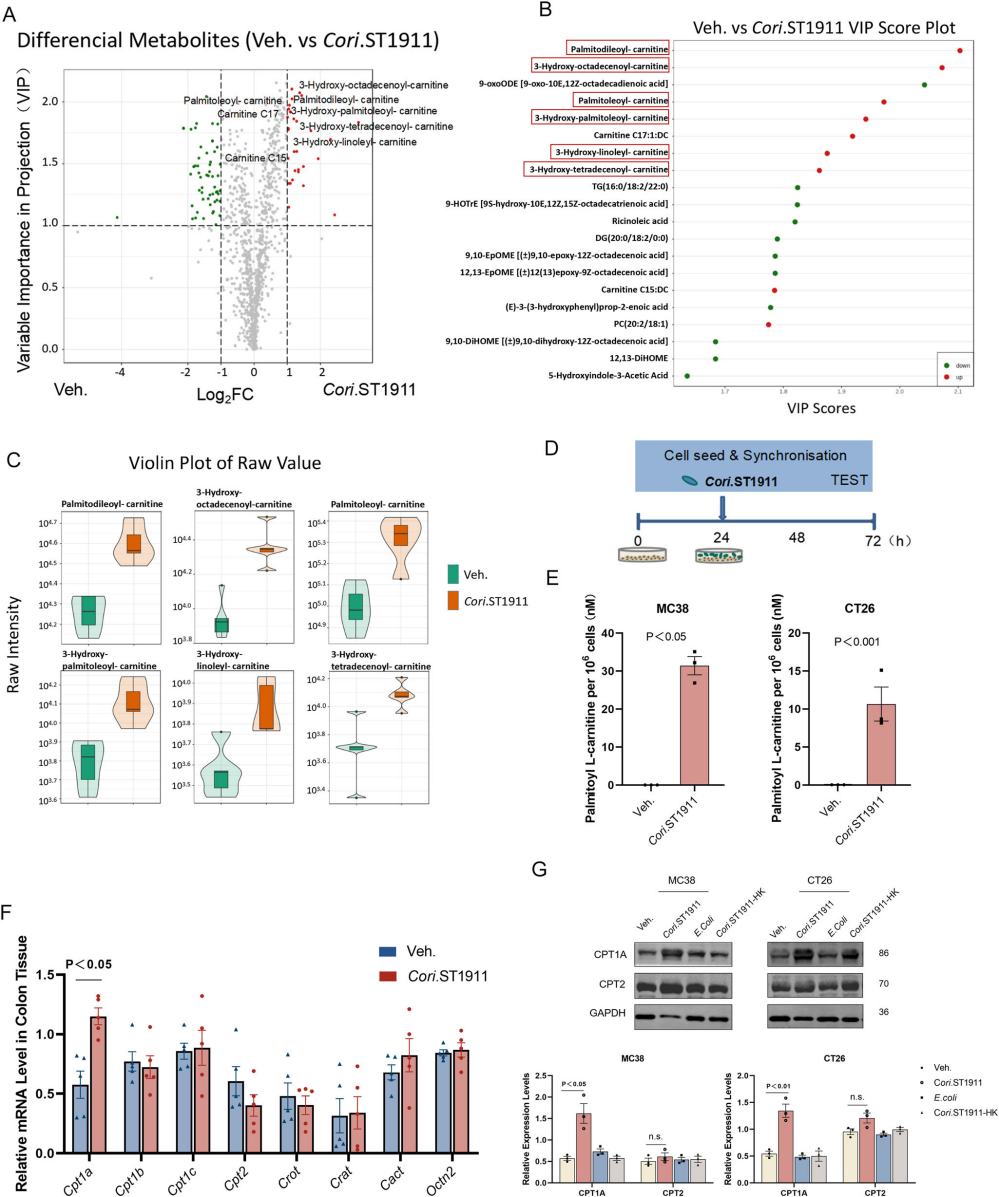
Enrichment of metabolite acylcarnitine is induced by *Coriobacteriaceae* through CPT1A upregulation. **A** Clustering analysis of ESI-Q TRAP-MS/MS metabolite analysis of *Cori*.ST1911 and GAM broth control group serum (Veh., *n* = 5; *Cori*.ST1911, *n* = 5). **B** Differential metabolites VIP of *Cori*.ST1911 and GAM broth control group serum. Red dots represent upregulated differentially expressed metabolites; green dots represent downregulated differentially expressed metabolites. **C** Violin plot of changes in serum acylcarnitine content of the *Cori*.ST1911 and GAM broth control groups. **D** Schematic diagram of the experimental procedure to construct model cell lines co-cultured with *Cori*.ST1911. **E** Intracellular palmitoyl L-carnitine concentration of cells treated with vehicle (*n* = 3) or *Cori*.ST1911 (*n* = 3). Data are represented as mean ± SEM, analysed using two-tailed Student’s t-test. **F** Quantitative RT-PCR analysis of mRNA expression related to acylcarnitine in colonic tissue of mice treated with *Cori*.ST1911 or GAM broth (*n* = 5). Data are represented as mean ± SEM, analysed using one-way ANOVA. **G** Representative western blots showing the effect of *Cori*.ST1911 treatment in cell lines. Heat-killed (HK) *Cori*.ST1911 and *E. coli* were used as controls. Proteins were quantified densitometrically using ImageJ software and analysed using two-tailed Student’s t-test. VIP, variable important in projection; GAM, Gifu anaerobic medium; HK, heat-killed.

To confirm whether *Cori*.ST1911 colonisation alters the host metabolic spectrum and promotes CRC, we performed full-spectrum LC-MS/MS metabolic analysis of sera from 20-week-old mice gavaged with *Cori*.ST1911 or GAM broth. Orthogonal partial least squares discriminant analysis (OPLS-DA) was used to screen differential metabolites, and hierarchical cluster analysis was performed to identify clusters of metabolites that accumulated due to *Cori*.ST1911 colonisation (Fig. 4A). The variable important in projection (VIP) values (Fig. 4B) and violin plots (Fig. 4C) show a prominent change in the long-chain acylcarnitine spectrum. As previous studies reported that acylcarnitine accumulation is linked to hepatocellular carcinoma driven by a HFD^15^, we focused on the carcinogenic mechanism of acylcarnitine in CRC development caused by *Cori*.ST1911. To determine whether *Cori*.ST1911 could directly stimulate acylcarnitine accumulation, colon cancer cell lines CT26 and MC38 were co-cultured with *Cori*.ST1911 for 48 h (Fig. 4D, MOI = 100). Palmitoyl-l-carnitine accumulation was detected in cells, indicating that *Cori*.ST1911 promotes intracellular acylcarnitine accumulation (Fig. 4E). However, higher levels of palmitoyl-l-carnitine were detected in the cell supernatant, indicating that palmitoyl-l-carnitine can be secreted into extracellular and circulatory systems (Supplementary Fig. 2).

We subsequently investigated molecules associated with acylcarnitine metabolism and accumulation in mouse tumour tissues by determining the mRNA expression of several genes involved in acylcarnitine biosynthesis, metastasis, and transport (Fig. 4F). The mRNA levels of carnitine palmitoyltransferase (*CPT1B*, *CPT1C*, and *CPT2*), carnitine octanoyltransferase (*CROT*), carnitine acetyltransferase (*CRAT*), carnitine/acylcarnitine translocase (*CACT*), and carnitine/organic cation transporter 2 (*OCTN2*) were not significantly different or only slightly decreased, whereas the mRNA level of *CPT1A* increased significantly in the *Cori*.ST1911-treated group. Although upregulated *CPT1A* expression was detected in *Cori*.ST1911 co-cultured cells (Supplementary Fig. 3A), there was no significant increase in *CPT2* expression (Supplementary Fig. 3B). These results suggest that CPT1 promotes the conversion of FA to acylcarnitine, but CPT2 does not increase the conversion of acylcarnitine to acyl-CoA, thereby explaining the significant acylcarnitine accumulation.

Western blotting and immunohistochemistry confirmed increased CPT1A protein expression in *Cori*.ST1911 co-cultured cells and colon tissue from 20-week-old mice gavaged with *Cori*.ST1911 or GAM broth, whereas CPT2 protein expression did not change significantly (Fig. 4G and Supplementary Fig. 3C). Furthermore, interference or inhibition of CPT1A inhibited acylcarnitine accumulation in cells (Supplementary Fig. 4). Moreover, no significant differences were seen in the levels of fatty acid oxidation (FAO) metabolites hydroxybutyric acid and acetyl-CoA, further confirming acylcarnitine accumulation (Supplementary Fig. 5).

**Fig. 5:**
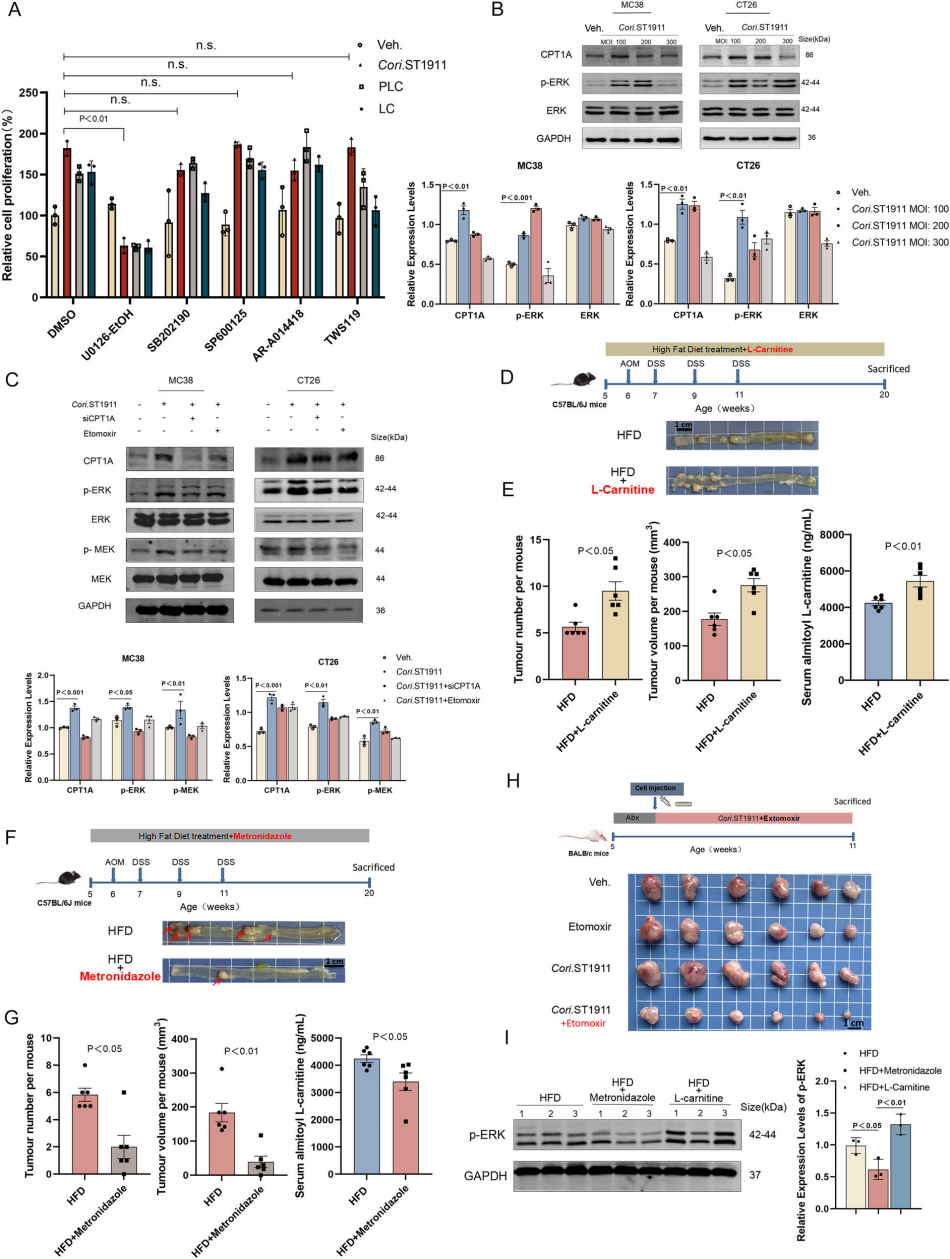
The CPT1A-MAPK pathway is involved in tumorigenesis caused by a high-fat diet. **A** Percentage cell proliferation of CT26 cells treated with MEK inhibitor U0126-EtOH (25 µM), JNK inhibitor SP600125 (25 µM), P38/MAPK inhibitor SB20.2190 (20 µM), GSK3β inhibitor tws119 (20 µM), or AR-A014418 (20 µM), and vehicle or *Cori*.ST1911. Cell OD was measured using the CCK8 method and normalised to cell proliferation of the control. Inhibitor treatment started 4 h before *Cori*.ST1911 treatment. Data are represented as mean ± SEM (*n* = 3 independent tests), analysed by one-way analysis of variance (ANOVA) with Bonferroni’s post-hoc test. n.s., no significance. **B** Representative western blots showing the effect of increasing *Cori*.ST1911 MOI (100-300) on CPT1A and p-ERK. **C** Representative western blots showing the effect of *Cori*.ST1911 on CPT1A and MAPK-related genes after treatment with SiCPT1A (48 h) or etomoxir (100 µM). Proteins were quantified densitometrically using ImageJ software and analysed using two-tailed Student’s t-test (**B**, **C**). **D** Schematic diagram of the experimental procedure of HFD and HFD-LC (L-carnitine, 100 mg/kg/day) treatment of C57BL/6J AOM mice, and representative colon tumour images. **E** Tumour number, tumour volume, and serum palmitoyl L-carnitine concentration in HFD- and HFD-LC-fed C57BL/6J AOM mice (*n* = 6). Data are represented as mean ± SEM, analysed using two-tailed Student’s t-test. **F** Schematic diagram of the experimental procedure of HFD and HFD-metronidazole treatment of C57BL/6J AOM mice, and representative colon tumour images. **G** Tumour number, tumour volume, and serum palmitoyl L-carnitine concentration in HFD- and HFD-metronidazole-fed C57BL/6J AOM mice (*n* = 6). Data are represented as mean ± SEM, analysed using two-tailed Student’s t-test. **H** Schematic diagram of the experimental procedure of *Cori*.ST1911 and *Cori*.ST1911-etomoxir treatment of subcutaneous transplanted tumour BALB/c mice, and representative colon tumour images. BALB/c mice were randomly divided into groups after subcutaneous injection of CT26 cells, followed by intragastric administration of *Cori*.ST1911 (1 × 10^8^ CFU once every 2 days) and intraperitoneal injection of etomoxir (15 mg/kg, 3 times/week). **I** Representative western blots showing MAPK-related protein expression in HFD-, HFD-LC-, and HFD-metronidazole-fed mice. Proteins were quantified densitometrically using ImageJ software and analysed using two-tailed Student’s t-test. MOI, multiplicity of infection; LC, L-carnitine; ERK, extracellular regulated protein kinases; MEK, Mitogen-activated protein kinase; MAPK, mitogen-activated protein kinase.

Screening of kinase inhibitors in combination with *Cori*.ST1911, carnitine, and palmityl carnitine to identify the pathways mediating the tumorigenic effect of *Cori*.ST1911 and acylcarnitine revealed that inhibition of MEK1/2 kinase activity inhibits cell proliferation induced by *Cori*.ST1911 and acylcarnitine (Fig. 5A). This suggests that *Cori*.ST1911 induces acylcarnitine accumulation by upregulating CPT1A expression, resulting in the activation of MEK/ERK signalling. These results are consistent with the immunoblotting results (Fig. 5B). Moreover, interfering with CPT1A or the addition of the CPT inhibitor etomoxir reversed *Cori*.ST1911-induced activation of the MAPK pathway (Fig. 5C). Our results showed that *Cori*.ST1911 activates the MAPK pathway by upregulating CPT1A and inducing acylcarnitine accumulation. Moreover, l-palmityl carnitine and l-carnitine can also activate the MAPK pathway, confirming our previous hypothesis (Supplementary Fig. 6). Notably, l-palmityl carnitine and l-carnitine can increase CPT1A expression without affecting CPT2 expression, forming a positive feedback loop leading to acylcarnitine accumulation.

Previous studies have linked red meat consumption to CRC occurrence and development^16^. As red meat is rich in carnitine, we hypothesised that carnitine supplementation combined with a HFD could promote CRC development. C57/BL6J AOM mice fed a HFD and l-carnitine-supplemented drinking water (Fig. 5D) exhibited an increase in the number and size of colon tumours (Fig. 5E). Furthermore, l-carnitine supplementation increased ERK phosphorylation in C57/BL6J AOM mouse tissues, consistent with the in vitro results (Fig. 5I). A HFD combined with carnitine supplementation therefore synergistically promotes CRC development, further supporting the tumour-promoting effect of acylcarnitine.

### Mechanism of HFD-induced tumour progression via the CPT1A-MAPK pathway and disrupted intestinal flora

To investigate whether intestinal flora disruption and the *Coriobacteriaceae*-CPT axis are crucial mechanisms in tumour development promoted by HFD-induced *Coriobacteriaceae* enrichment, we administered the antibiotic metronidazole to HFD-fed C57/BL6J AOM mice (Fig. 5F). The results revealed that, compared with the control group, *Coriobacteriaceae* are not enriched when a HFD is combined with metronidazole (Supplementary Fig. 7A), which inhibits the carcinogenic effect of the HFD and serum acylcarnitine accumulation (Fig. 5G). Immunohistochemistry results revealed that CPT1A expression did not increase, while tumour cell proliferation factor Ki67 expression was significantly reduced (Supplementary Fig. 7B). Moreover, metronidazole inhibited the HFD-induced increase in ERK phosphorylation (Fig. 5I).

Metronidazole is a nitroimidazole antibiotic that has antibacterial effects against most anaerobic bacteria but the inhibitory effect of metronidazole can only partially explain the intestinal flora disruption.

The mechanism by which a HFD promotes CRC was verified by oral administration of *Cori*.ST1911 combined with intraperitoneal injection of the CPT1-specific inhibitor etomoxir into BALB/c mice with subcutaneously transplanted tumours (Fig. 5H). The results revealed that the carcinogenic effect of *Cori*.ST1911 was reversed when combined with etomoxir (Supplementary Fig. 8), which suggest that *Cori*.ST1911 promotes tumour development by upregulating CPT1A expression.

### Effect of *Lactobacillus murinus* on the colonisation and tumorigenic effect of *Cori*.ST1911

Preliminary analysis of intestinal flora revealed significantly lower *Lactobacillus* abundance in HFD-fed mice than in wild-type (WT) mice (Supplementary Fig. 9). *Lactobacillus*, a widely used probiotic, regulates intestinal ecological balance, repairs intestinal mucosal immunity, and maintains normal intestinal function^17^. We therefore hypothesised that *Lactobacillus* re-colonisation may reverse the intestinal bacterial imbalance caused by a HFD. Furthermore, previous studies reported that enrichment of *Coriobacteriaceae* in the intestine of HFD-fed mice is negatively correlated with intestinal barrier integrity^18^. We therefore hypothesised that *Lactobacillus* from the typical intestinal environment could antagonise *Cori*.ST1911 colonisation and enrichment in HFD-fed mice, thereby preserving intestinal barrier integrity and reversing the carcinogenic effect of *Cori*.ST1911.

Two *Lactobacillus* strains were isolated from the fresh stool of healthy WT mice and identified as *Lactobacillus murinus* and *Lactobacillus johnsonii*, and named *La.mu*730 and *La.jo*181, respectively. *La.mu*730 and *La.jo*181 were gavaged into HFD-fed mice, and it was found that both species antagonised *Cori*.ST1911 colonisation, inhibiting *Cori*.ST1911 enrichment (Supplementary Fig. 10A, B). *La.mu*730 antagonised *Cori*.ST1911 colonisation more rapidly than *La.jo*181, and its antagonistic effect was more pronounced (Supplementary Fig. 10C, D).

**Fig. 6:**
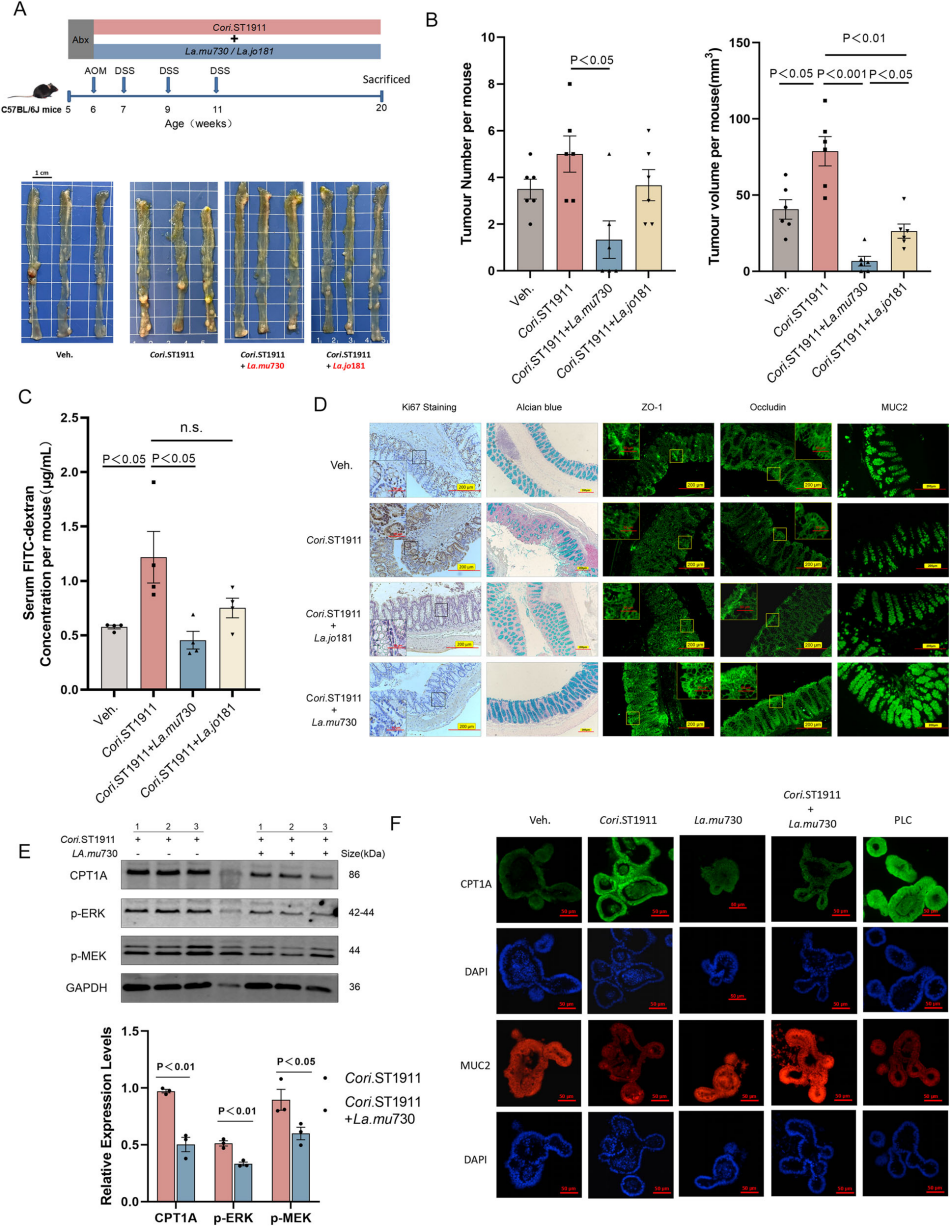
*Lactobacillus murinus* ameliorates tumour development induced by *Cori*.ST1911. **A** Schematic diagram of the experimental procedure of C57BL/6J AOM mice gavaged with *Cori*.ST1911, and *Cori*.ST1911 combined with *La.mu*730 or *La.jo*181, and representative colon tumour images. After 1 week of antibiotic treatment, C57BL/6J AOM mice were gavaged with combined *Cori*.ST1911 and *La.mu*730 or *La.jo*181 (1 × 10^8^ CFU of each strain) every 2 days. **B** Tumour number and volume of C57BL/6J AOM mice gavaged with *Cori*.ST1911 and *Cori*.ST1911 combined with *La.mu*730 or *La.jo*181 (*n* = 6). Data are represented as mean ± SEM, analysed using one-way ANOVA. **C** Serum FITC dextran concentration was measured 4 h after intragastric glycoside-binding fluorescein isothiocyanate administration from 6 to 20 weeks (*n* = 4). Data are represented as mean ± SEM, analysed using one-way ANOVA; n.s., no significance. **D** Representative images of IHC of tissue nuclear Ki67, TJ protein (ZO-1, Occludin), and mucin-related protein (MUC2, Alcian blue) of C57BL/6J AOM mice gavaged with *Cori*.ST1911 and *Cori*.ST1911 combined with *La.mu*730 or *La.jo*181. Scale bars: 200 µm. **E** Representative western blots showing CPT1A and MAPK-related protein expression in the *Cori*.ST1911 and *Cori*.ST1911 combined with *La.mu*730 groups. Proteins were quantified densitometrically using ImageJ software and analysed using two-tailed Student’s t-test. **F** Immunofluorescence staining of CPT1A and MUC2 in mouse intestinal organoids separated from WT mice and co-cultured with PLC (75 μM), *Cori*.ST1911, *La.mu*730, and *Cori*.ST1911 combined with *La.mu*730 (MOI = 100) for 24 h. TJ, tight junctions; PLC, palmitoyl L-carnitine; ERK, extracellular regulated protein kinases; MEK, mitogen-activated protein kinase; IHC, immunohistochemistry.

The viability and adhesion of *Cori*.ST1911 was evaluated by co-incubating *Lactobacillus* and *Coriobacteriaceae* at the same MOI with CT26 and MC38 cells (Supplementary Fig. 11A). The results showed that co-incubation did not decrease *Cori*.ST1911 viability but significantly weakened the adhesion of *Cori*.ST1911, indicating the antagonistic effect of *La.mu*730 on *Cori*.ST1911 is not related to bacterial viability (Supplementary Fig. 11B). Evaluation of the effect of *La.mu*730 colonisation on the carcinogenic effect of *Cori*.ST1911 revealed significantly reduced tumour number and volume after intragastric administration of *La.mu*730 combined with *Cori*.ST1911 compared with administration of only *Cori*.ST1911 (Fig. 6A, B). To confirm the colonisation effect of *La.mu*730, the stool of mice in the combined gavage group was collected and analysed over several weeks. The results indicated that *La.mu*730 has a significant antagonistic effect on *Cori*.ST1911 colonisation, confirming *La.mu*730 as the intestinal effector strain (Supplementary Fig. 12).

Assessment of intestinal permeability using fluorescein isothiocyanate (FITC)-dextran revealed intact intestinal wall structure in mice that received combined *La.mu*730 and *Cori*.ST1911 (Fig. 6C). Alcian blue and immunohistochemical staining for MUC2, ZO-1, and occludin expression in tumour tissues revealed increased mucin layer-related and tight junction protein expression in the intestinal wall, which strengthen the intestinal barrier, in the combined group. In contrast, CPT1A, p-MEK and p-ERK expression decreased in tumour tissues (Fig. 6D, E).

In recent years, mouse intestinal organoids have been used to verify the mechanisms underlying the interaction between microorganisms and the intestine. In this study, we found that *La.mu*730 reversed the *Cori*.ST1911-induced activation of CPT1A and destruction of mucin-related proteins, such as MUC2, in intestinal organoids (Fig. 6F). Our results indicated that *La.mu*730 promotes mucus production in goblet cells to ensure intestinal mucosal barrier integrity, thereby antagonising *Cori*.ST1911 colonisation and inhibiting *Cori*.ST1911-induced activation of the CPT1A-ERK axis.

## Discussion

Numerous studies have shown that a HFD is significantly associated with the development of CRC^19^, altering bile acid profiles and driving malignant transformation in Lgr5^+^ intestinal stem cells or activating FAO to promote tumour progression^20,21^. The relationship between a HFD and the gut microbiome, as well as the associated role in multiple diseases, has been increasingly investigated in recent years^22,23^. Although a HFD can alter the intestinal *Firmicutes*/*Bacteroidetes* ratio^7^, the influence on other species or genera require further investigation. Several studies have reported that a HFD is associated with *Coriobacteriaceae* enrichment^24^, and that *Coriobacteriaceae* are positively correlated with obesity^25^. Moreover, *Coriobacteriaceae* regulate obesity and host lipid metabolism in response to a HFD^26,27^, and several *Coriobacteriaceae* strains have been isolated from patients with obesity or intestinal inflammation^28,29^. We therefore investigated whether HFD-induced *Coriobacteriaceae* enrichment is related to CRC occurrence and development.

Previous studies have reported that alterations in intestinal flora are linked to tumorigenesis and tumour progression^30^. However, whether a HFD can promote CRC occurrence by altering intestinal flora remains unclear. A recent study by Yang et al. found that a HFD can enrich the genus *Alistipes*, leading to an increase in LPA levels, and promoting colon cancer progression^31^. We therefore investigated the carcinogenic mechanism by which *Coriobacteriaceae* could promote CRC development.

Previous studies investigating bacterial diversity in CRC using 16S rRNA gene sequencing and metagenomic analysis found that *Coriobacteriaceae* are enriched in the faeces of patients with CRC, suggesting that these bacteria are related to CRC progression^11,32,33^. In this study, the abundance of *Coriobacteriaceae* after a HFD increased significantly with tumour progression (Supplementary Fig. 10B), indicating that a HFD can promote CRC progression by altering the gut microbiome and that *Coriobacteriaceae* play an important role in this mechanism.

Our data also showed enrichment of *Lachnospiraceae* and *Peptostreptococcaceae* in the HFD group, which corresponds with previous reports^34^. However, the *Coriobacteriaceae* enrichment was more significant. Bacteria such as *Fusobacterium nucleatum*^35^, *Bacteroides fragilis*^36^, *psk*^*+*^ *Escherichia coli*^37^, and *Peptostreptococcus* often referred to as “driver bacteria” in disease development, whereas *Coriobacteriaceae* are referred to as “passenger bacteria” in CRC^38^. At different stages of tumour development, driver and passenger bacteria may be interchanged to promote tumour growth^38^. To investigate the impact of *Cori*.ST911 colonisation on the ecological niche of other carcinogenic bacteria, we performed 16s rRNA sequencing on the intestinal bacteria of mice after *Cori*.ST911 gavage (Supplementary Fig. 13) and found that *Cori*.ST911 colonisation and a HFD diet induced distinct changes in intestinal bacteria. Increased proteobacteria was previously considered to be related to a HFD^39^. In contrast, we found that *Cori*.ST911 colonisation decreased short-chain fatty acid-producing bacteria, such as *Allobaculum*, *Ruminococcaceae*, and *Paraacteroides*^40,41^, while increasing the abundance of opportunistic pathogens, such as *Enterrhabdus* and *Eggerthellaceae*, thus disrupting the intestinal environment^42,43^.

Changes in intestinal bacteria are affected by multiple factors such as age, gender, host species, and diet^44^. The manner in which a HFD alters the intestinal microbiome composition and function is also affected by various factors, including the amount or type of fat consumed, the animal strain, disease model, and experimental design^45,46^. Selecting the appropriate control feed, sampling time, and experimental design are therefore essential for studying the influence of dietary interventions on intestinal microbiota^47^.

Changes in intestinal microbial composition is associated with changes in serum metabolite levels^48^. A HFD can cause acylcarnitine accumulation, which is associated with obesity-induced inflammation^49,50^. Furthermore, a HFD can down-regulate CPT2 expression in mice, leading to serum acylcarnitine accumulation, enhancing STAT3 activation, and promoting hepatocellular carcinoma occurrence of in vivo^15^. However, the role of these compounds and pathways in colorectal carcinogenesis remain unclear. Although carnitine plays a positive role in normal FAO, acylcarnitine produced by carnitine supplementation accumulates when FAO is inhibited, especially when the CPT1/CPT2 transport axis is compromised^51^. Our results demonstrated that a HFD promotes CPT1A expression through *Cori*.ST1911 enrichment, whereas CPT2 expression remains unchanged. Acylcarnitine accumulation can therefore promote CRC occurrence and development. These results offer a new perspective on CRC promotion in response to acylcarnitine accumulation after HFD administration. Although we showed that increased MAPK activation partially explains *Coriobacteriaceae* enrichment in obesity-related CRC, the mechanism underlying CPT1A upregulation in CRC remains unknown and require further study.

Dietary fat can directly enhance intestinal permeability by modulating tight junction distribution and intestinal mucus composition, stimulating the secretion of hydrophobic bile acids, and activating pro-inflammatory signalling cascades^52^. In addition, a HFD enriches the gut microflora with barrier-disrupting species that impact gut barrier functionality directly through secreted products or indirectly through immune system activation^53^. In this study, we found that *Coriobacteriaceae* destroy the intestinal wall barrier integrity by downregulating tight junction function-related molecules, thereby causing the secretion of harmful metabolites, and promoting carcinogenesis. Some microorganisms colonising the intestine, such as *Bacteroides thetaiotaomicron*, can express sulphatase, use O-glycan as a nutrient source, degrade mucin, and destroy the intestinal barrier^54^. De novo sequencing revealed that *Cori*.ST1911 carries the arylsulfatase gene TGY62484.1 and further studies to determine whether *Cori*.ST1911 can degrade intestinal mucin via sulfatase are therefore required to investigate the causal relationship between *Cori*.ST1911 colonisation and intestinal barrier destruction.

*Lactobacillus* can repair the intestinal mucosa and is widely used as a probiotic^17^. In this study, we isolated and screened two novel strains of *Lactobacillus*, but only *La.mu*730 significantly inhibited *Cori*.ST1911 colonisation and repaired the intestinal barrier. In conclusion, intestinal microecological disorders caused by a HFD are associated with CRC; however, further verification of this association is required. Studying intestinal flora enriched by a HFD may allow the identification of promising therapies for HFD-induced dysbiosis. In this study, a novel strain of *Coriobacteriaceae* was isolated from the intestine of HFD-fed mice, and further analysis showed that *Coriobacteriaceae* might play an important role in the regulatory mechanisms underlying HFD-associated CRC, thereby providing alternative intestinal bacterial markers and therapeutic targets CRC, especially for patients with obesity or consuming Western-style diets. Comprehensive metabolomic analyses identified extensive acylcarnitine accumulation following CPT1A upregulation by *Coriobacteriaceae*, which then activated the MAPK signalling pathway, ultimately causing progression of colorectal tumours. We also isolated several other novel strains of bacteria that were significantly related to a HFD and further verified their correlation with CRC occurrence and development. Our results therefore suggest that regulating intestinal microbiota composition and function is an effective approach for combating diet-induced metabolic diseases, including CRC, and that *Coriobacteriaceae* may serve as a bacterial marker for colon cancer diagnosis.

## Methods

### Bacteria

Strain *Cori*.ST1911 was isolated from fresh stool of 20-week-old C57/BL6J mice fed a HFD at the Animal Center of the West China Hospital of Sichuan University. Briefly, faecal particles were ground with a glass grinding rod and suspended in sterile phosphate-buffered saline (PBS). After gradient dilution, the suspension was coated on a Gifu anaerobic medium (GAM) supplemented with 0.15% hemin chloride, 0.15% vitamin K1, and 5% defibrillated sheep blood (Supplementary Table 2), and anaerobically cultured at 37 °C for 48 h. Thirty-three colonies with different shapes and colours were selected and sequenced using pan-bacterial primers targeting 16S rRNA genes. ANI comparison in the NCBI database combined with macrogenome sequencing indicated 97% sequence identity between *Cori*.ST1911 and other *Coriobacteriaceae* species. The *L. murinus* strain *La. mu*730 was isolated from the stool of healthy wild-type C57/BL6J mice, and anaerobically cultured on MRS basic medium at 37 °C for 48 h.

### Animals

C57BL/6J and BALB/c mice (4–5 weeks old) were purchased from Beijing HFK Bioscience. C57BL/6J-Apc^Min^ (Apc^Min/+^) mice were obtained from the Model Animal Research Center of Nanjing University (Nanjing, China). Breeding Apc^Min/+^ mice were used as experimental mice, while wild-type offspring from the same litter served as controls. All animals were kept in specific-pathogen free miniature isolation cages at The Animal Center of West China Hospital, Sichuan University. All experiments were performed in accordance with relevant guidelines and regulations, and were approved by the Institutional Animal Care and Animal Ethics Committee of Sichuan University (No. 20211262A). Mice were fed a normal diet for 1 week prior to the HFD. The mice were then randomly divided into two groups and fed either a normal control diet (CD; M10110C2, Moldiets, China) or a high-fat diet (HFD; M10160, Moldiets, China). The ingredients of the diet are listed in Supplementary Table 3. To avoid cage effects, each group of mice was placed in at least two different cages.

### Establishment of the mouse primary cancer model and subcutaneous transplanted tumour model

Five-week-old male C57BL/6J mice were kept under standard conditions and fed a standard diet for 1 week to adapt to the environment. The mice were then divided into groups: CD, HFD, HFD combined with L-carnitine, HFD combined with metronidazole, vehicle (Veh.), *Cori*.ST1911, *Cori*.ST1911 combined with *La.mu*730, and *Cori*.ST1911 combined with *La.jo*181. The bacterial-gavage experimental group and the control group received antibiotic cocktail (Neomycin 0.2 g/L, metronidazole 0.2 g/L, ampicillin 0.2 g/L, vancomycin 0.1 g/L) pretreatment for 1 week. The mice were subsequently gavaged with 1 × 10^8^ CFU per mouse every two days beginning at the age of 6 weeks and continued until the end of the experiment. GAM broth was used as vehicle control. Following the colon carcinogenesis model method^55^, the mice then received a single intraperitoneal (i.p.) injection of azomethane (AOM; 10 mg/kg; Sigma-Aldrich, Germany) and three cycles of dextran sodium sulfate (DSS; 1.5% Sigma-Aldrich, Germany) dissolved in the drinking water. Mice were euthanised before dissection.

For the subcutaneous transplanted tumour model, 1 × 10^6^ CT26 cells were inoculated into the left armpit of female BALB/c mice (4–5 weeks old), and the tumour volume was continuously measured. The mice received 1 week of antibiotic cocktail pretreatment followed by gavaged with 1 × 10^8^ CFU per mouse every 2 days beginning at 6 weeks of age and continued until the end of the experiment. Experimental mice received i.p. etomoxir (15 mg/kg, 3 times/week) injections and control mice were injected with solvent. Tumour length and diameter was measured every 3 days with a vernier calliper and the tumour volume was calculated. After 3–4 weeks, the mice were euthanised.

### Cell culture

Mouse colon cancer cell lines CT26 and MC38 were exposed to *Cori*.ST1911 (multiplicity of infection, MOI = 100) for 6 h/day for two consecutive days. After 6 h, the medium containing *Cori*.ST1911 was replaced with Dulbecco’s modified Eagle medium (DMEM) supplemented with penicillin (100 ui/mL)/streptomycin (100 μg/mL) and 100 μg/mL gentamicin. CT26 cells treated with phosphatase inhibitor (U0126-EtOH, HY-12013; SP600125, HY-12041; SB202190, HY-10295, TWS119, HY-10590; AR-A014418, HY-10512, MCE, USA)before Cori.ST1911 treatment and Cell OD was measured using the CCK8 method.

### 16S rRNA sequencing

The CTAB (cetyltrimethylammonium bromide) method was used to extract total genomic DNA. DNA concentration and purity were determined using 1% agarose gels. For amplification, environmental samples and pure water were used as negative controls to exclude faecal contamination. DNA was diluted to 1 ng/µL in sterile water. Distinct regions of the 16S rRNA genes (16S V4/16S V3/16S V3-V4) were amplified using barcoded primers (16S V4:515F-806R). All PCRs were performed using 15 µL Phusion® High-Fidelity PCR Master Mix (New England Biolabs), 2 µM forward and reverse primers, and approximately 10 ng template DNA. Thermal cycling involved initial denaturation at 98 °C for 1 min, followed by 30 cycles of denaturation at 98 °C for 10 s, annealing at 50 °C for 30 s, and elongation at 72 °C for 30 s. PCR products were mixed with an equal volume of 1× loading buffer containing SYBR green, and electrophoresis was performed on 2% agarose gel. PCR products were mixed in equidensity ratios and purified using Qiagen Gel Extraction Kits (Qiagen, Germany). Sequencing libraries were constructed using TruSeq® DNA PCR-Free Sample Preparation Kits (Illumina, USA) according to the manufacturer’s instructions, and index codes were added. The library quality was assessed using a Qubit@ 2.0 Fluorometer (Thermo Fisher Scientific, USA) and Agilent Bioanalyzer 2100 system. Finally, the library was sequenced using an Illumina Nova Seq platform, yielding 250 bp paired-end reads.

To obtain high-quality sequencing data, V3-V4 region pools were sequenced at a final concentration of 12 pM with a 10% PhiX control spike-in using an Illumina MiSeq 500 cycle V3 kit and 2 × 300 run configuration. Fastp (https://github.com/OpenGene/fastp, version 0.20.0) was used for quality control of the original sequencing sequence and FLASH (http://www.cbcb.umd.edu/software/flash, version 1.2.7) was used for splicing. Bases with a tail mass value of less than 20 were filtered and removed from the reads. Based on the overlap between paired-end reads, paired reads were merged into a sequence with a minimum overlap of 10 bp. A maximum mismatch ratio of 0.2 was allowed in the overlap region of the splicing sequence and non-matching sequences were screened and removed. Samples were distinguished based on the barcodes and primers at the beginning and end of the sequence, and the sequence direction was adjusted. Zero mismatch was allowed for barcodes, and a maximum mismatch of 2 was allowed for primers.

The UPARSE OTU algorithm, using the usesrch10.0 standard, was used for OTU clustering analysis of sequences obtained from the Clean Tags sequence of each sample with a similarity of 97% (Supplementary Table 4). The alpha diversity index, including CHAO1, goods_coverage, observed_species, and beta diversity distance, including Bray-Curtis distance and (Un) weighted unifrac, were calculated separately using qiime (Ver.1.9.1, http://qiime.org/scripts/index.html). Linear discriminant analysis (LDA) was performed according to different grouping conditions according to taxonomy composition using linear discriminant analysis effect size (LEfSe, http://huttenhower.sph.harvard.edu/LEfSe; LDA > 2, *P* < 0.05), to identify species exhibiting significant differences in sample division.

### Electron microscopy

A sputter coater was used to coat *Cori*.ST1911 samples harvested from agar plates with gold (Cressington 108, Cressington Scientific Instruments, UK). Images were captured using a scanning electron microscope (EVO 10; Carl Zeiss, Germany) at an accelerating voltage of 20 kV.

### Intestinal FITC-dextran test

D-glycoside-binding fluorescein isothiocyanate (FITC-dextran, 68059, Sigma, Germany) was dissolved in phosphate buffer to prepare a 100 mg/mL working solution that was stored in the dark until use. The mice were gavaged with 0.6 mg/g after a 12 h fast. After 4 h, blood was drawn from the orbit, left at room temperature for 60 min, and centrifuged at 2000 rpm for 15 min. Serum and phosphate buffer were mixed in a 1:1 ratio, added to each well of a 96-well plate, and absorbance detected at 520 nm using a microplate reader. Serum FITC-dextran mass concentration was calculated using a FITC-dextran standard curve.

### Metabolome analyses

Sample extracts were analysed using an LC-ESI-MS/MS system (UPLC, Shim-pack UFLC Shimadzu CBM A system, https://www.shimadzu.com/; MS, QTRAP® System, https://sciex.com/). Linear Ion Trap (LIT) and triple quadrupole (QQQ) scans were acquired on a QQQ-linear ion trap mass spectrometer QTRAP® LC-MS/MS system equipped with an electrospray ionization (ESI). The turbo ion-spray interface operates in positive and negative ion mode and is controlled by Analyst 1.6.3 software (Sciex). The ESI source operation parameters were as follows: source temperature 500 °C; ion spray voltage (IS) 5500 V (positive), −4500 V (negative); ion source gas I (GSI), gas II (GSII), and curtain gas (CUR) pressure were set at 55, 60, and 25.0 psi, respectively; and the collision gas (CAD) was high. Instrument tuning and mass calibration were performed with 10 and 100 µmol/L polypropylene glycol solutions in QQQ and LIT modes. For each period, a specific set of MRM transitions were monitored depending on which metabolites eluted within this period.

### Organoid culture and detection

C57BL/6J mouse colon tissue was cut into pieces, washed 15 times with PBS buffer free of calcium and magnesium ions, and digested in Gentle Cell Dissociation Reagent (GCDR; 07174, Stemcell, Canada) at room temperature (15–25 °C) for 20 min. To separate crypt cells from the intestinal tissue, the digestion solution was discarded and the resuspended colon tissue fragments were blown by Pasteur pipette with PBS buffer containing 0.1% bovine serum albumin (BSA). The suspension was passed through a 70-µm cell sieve and the supernatant was collected by centrifugation at 290 × *g* at 2–8 °C for 5 min to obtain colonic recess cells. Crypt cells were resuspended in DMEM F12 medium (36254, Stemcell, Canada), counted, and incubated in a recess matrix glue suspension composed of a 1:1 ratio of IntestiCult organ growth medium (06005, Stemcell, Canada) and undiluted Matrigel (356231, Corning, USA). The suspension was inoculated into a 24-well plate and incubated at 37 °C for 15 min. After the recess matrix glue suspension solidified, 700 µL IntestiCult organ growth medium was added and cultured at 37 °C in a 5% CO_2_ constant-temperature incubator. The culture medium was changed every 2 days.

Mature organoids were washed three times with PBS, fixed with 4% paraformaldehyde solution for 30 min at room temperature, and treated with 30% sucrose solution overnight at 4 °C. Finally, the colonic organoids were embedded in an OCT (optimal cutting temperature compound) embedding agent and cut into 8-µm-thick sections using a LEICA CM1950 system. The slices were soaked in pure water for 1 min to remove excess OCT embedding agent and repaired using a citrate-buffered solution in a microwave for 10 min. The sections were incubated with corresponding primary and secondary antibodies, nuclei were stained with DAPI (C1006, Beyotime, China) for 10 min, and images were captured using an upright fluorescence microscope. The primary antibodies were CPT1A (1:500, 15184-1-AP, Proteintech, China) and MUC2 (1:500, ab272692, Abcam, USA), and the secondary antibodies were goat anti-rabbit IgG H&L (Alexa Fluor 488, 1:5000, ab150077, Abcam, USA).

### LC-MS/MS

LC-MS/MS was performed using a Shimadzu ultra-fast liquid chromatography system (UFLC; Shimadzu, Japan) coupled with an AB SCIEX QTRAP 5500 mass spectrometer equipped with a turbo spray ion source. Analyst 1.6.2 software (AB SCIEX, USA) was used to collect and analyse chromatographic and mass spectrometry data. Chromatographic separation was achieved using a Waters ACQUITY UPLC BEH C18 column (2.1 mm × 100 mm ID, 1.7 µm) with a mobile phase consisting of 0.1% formic acid in water (A) and acetonitrile (B). Gradient elution was performed with 0–1.0 min, 10–90% B and 1.0–2.0 min, 90% B at a flow rate of 0.5 mL/min. Column and autosampler temperatures were maintained at 35 °C and 15 °C, respectively. The injection volume was 1 µL. MS/MS sample detection was performed in positive ionisation mode with optimised mass spectrometric parameters, namely 5500 V ion spray voltage and 100 V declustering potential at 500 °C. Multiple reaction monitoring (MRM) mode was selected for quantifying acylcarnitine and internal standard with ion pairs of 400.2–85.0 and 265.2–232.2, respectively.

For the cell line studies, cells were plated in 10 cm wells at a density of 1 × 10^6^ cells/well and cultured in DMEM supplemented with 10% serum for 24 h. The medium was then replaced by DMEM supplemented with 15 µM sertraline, incubated for an additional 12 h, and the cells harvested for metabolite measurements. Metabolite concentrations were normalised to the total cell number.

### Western blotting

Cells were lysed using a universal protein lysate extraction reagent (pp1801, Bioteke, China). Samples were shaken and lysed on ice for 30 min, mixing every 10 min. Samples were then centrifuged at 13,400 × *g* for 15 min at 4 °C. The supernatant was removed, 5× sample buffer was added, and the suspensions were incubated in boiling water for 10 min to obtain the total cell protein. Fresh protein samples were measured using a microplate reader (Model 680, BioRad), and equal amounts of lysate were run on an SDS-PAGE gel, followed by a standard western blotting protocol. All blots or gels derive from the same experiment and that they were processed in parallel. Primary antibodies included CPT1A (1:1000, 15184-1-AP, Proteintech, China), p-ERK (1:2000, ab201015, Abcam, USA), ERK (1:2000, ab184699, Abcam, USA), CPT2 (1:1000, 26555-1-AP, Proteintech, China), MEK (1:1000, ab178876, Abcam, USA), p-MEK (1:2000, 2338, Cell Signaling, USA), and GAPDH (1:2000, TA-08, ZSGB-BIO). Secondary antibodies included IRDye 800CW goat anti-rabbit IgG (H&L) (926-32211, Licor, USA), IRDye 680CW goat anti-mouse IgG (H&L) (926-68070, Licor, USA), and anti-HRP rabbit polyclonal antibody (D110011, Sangon Biotech, China). Gels and blots in this article derive from the same experiment or samples processed in parallel. Original blots are provided in Supplementary Materials.

#### Bacterial DNA extraction and qPCR analysis

Bacterial DNA was extracted from stool samples using EZNA faecal DNA Kits (Omega Biotek, USA). Extracted DNA purity was determined using a NanoDrop 2000 spectrophotometer (Thermo Fisher Scientific, USA). The cDNA was diluted with RNase/DNase-free water. Specific primers were used to evaluate *Cori*.ST1911 and *La.mu*730 abundance with qPCR and normalised to pan-bacterial 16S rRNA gene expression. The bacterial primer sequences are listed in Supplementary Table 5. Quantitative PCR was performed in triplicate and the relative abundance of bacteria was calculated using the 2^−ΔΔCT^ method^56^.

### Cellular RNA extraction and qPCR analysis

Cell Total RNA Isolation Kits (RE-03113, Foregene, China) were used to extract total RNA, and 1 µg isolated RNA was reverse-transcribed into cDNA using Primescript™ RT Reagent Kits (RR047a, Takara, Japan). The synthesised cDNA was diluted with RNase/DNase-free water. SYBR Green qPCR Master Mix (B21202, Bimake, China) was used for real-time fluorescence qPCR detection. The primer sequences used in this study are listed in Supplementary Table 5.

### Immunofluorescence and immunohistochemistry

Colon tissues were fixed with 10% neutral formalin for 24 h. Tissue wax blocks were created via gradient ethanol dehydration, clearing, and wax immersion. Tissue sections were dewaxed with gradient ethanol, soaked in sodium citrate antigen, antigen-repaired by boiling in a microwave oven for 20 min, blocked with goat serum for 20 min at room temperature, and then incubated with the primary antibodies overnight at 4 °C. The sections were subsequently incubated with the corresponding secondary immunofluorescence antibody for 20 min at room temperature and stained with DAPI for 10 min. Images were captured using fluorescence microscopy. The primary antibodies used for immunofluorescence were MUC2 (1:500, ab272692, Abcam, USA), ZO-1 (1:500, ab221547, Abcam, USA), and occludin (1:500, ab216327, Abcam, USA). The secondary antibodies were goat anti-rabbit IgG H&L (Alexa Fluor 488, 1:5000, ab150077, Abcam, USA).

The immunohistochemical procedure was the same as that described above, using Universal SP reagent kits (mouse/rabbit Streptomyces ovalbumin biotin detection system, SP9000, Zhongshan Jinqiao, China). After incubation with the secondary antibodies, DAB (ZLI-9018, Zhongshan Jinqiao, China) reagent was used for colour development, and images were captured using an upright microscope. The primary antibodies used for immunohistochemical analyses were CPT1A (1:500, 15184-1-AP, Proteintech, China), CPT2 (1:500, 26555-1-AP, Proteintech, China), and Ki67 (1:500, ab15580, Abcam, USA).The secondary antibodies were Biotin-labelled goat anti-rabbit IgG polymer (SP9000, Zhongshan Jinqiao, China).

### Alcian blue staining

Tissue sections were dewaxed and dehydrated with an ethanol gradient, incubated in acetic acid for 3 min followed by incubation with Alcian blue (pH 2.5) for 30 min, and rinsed with water. The sections were then stained with Nuclear Fast Red for 5 min. The staining process was controlled under a microscope. The sections were then rinsed with water, dehydrated, and images were captured using a microscope (Nikon, TS100-F, Japan).

### Statistical analysis

Statistical analyses were performed using GraphPad Prism software version 9.3.1 (GraphPad, La Jolla, CA, USA). Each experiment was performed at least three times. Individual values are displayed as scatter plots with histograms. All data are presented as the mean ± standard error of the mean (SEM). A two-tailed unpaired Student’s t-test was used to evaluate statistical significance between two groups. Multigroup data were compared using one-way analysis of variance (ANOVA) followed by Bonferroni or Tukey’s post-hoc tests. The inspection level was set at *α* = 0.05 and statistical significance was set at *P* < 0.05.

### Reporting summary

Further information on research design is available in the Nature Research Reporting Summary linked to this article.

### Supplementary information

Supplementary information

Supplementary Table 4

Reporting Summary

## Data Availability

Datasets of the 16S rRNA and metagenome are available in the National Center for Biotechnology Information under BioProject accession numbers PRJNA892732 and PRJNA892765, respectively. The de novo *Cori*.ST1911 genome sequences are available under BioProject accession number PRJNA850882. Raw data for serum metabolomics are presented in Supplementary Table 6. 16S rRNA sequencing OTU data are presented in Supplementary Table 4. All other data generated by or analysed in this study are available from the corresponding authors upon request.
