# Supplementary material for: Endogenous Coriobacteriaceae enriched by a high-fat diet promotes colorectal tumorigenesis through the CPT1A-ERK axis
Source: NPJ Biofilms Microbiomes. 2024 Jan 20;10:5. doi: 10.1038/s41522-023-00472-7 (PMC10799938; doi:10.1038/s41522-023-00472-7)
Supplement: Supplementary file 1 — Supplementary information [file 41522_2023_472_MOESM1_ESM.pdf]

## Supplementary materials for Endogenous *Coriobacteriaceae* Enriched by a High-Fat Diet Promotes Colorectal Tumorigenesis Through the cpt1a-erk Axis

## Supplementary Figures

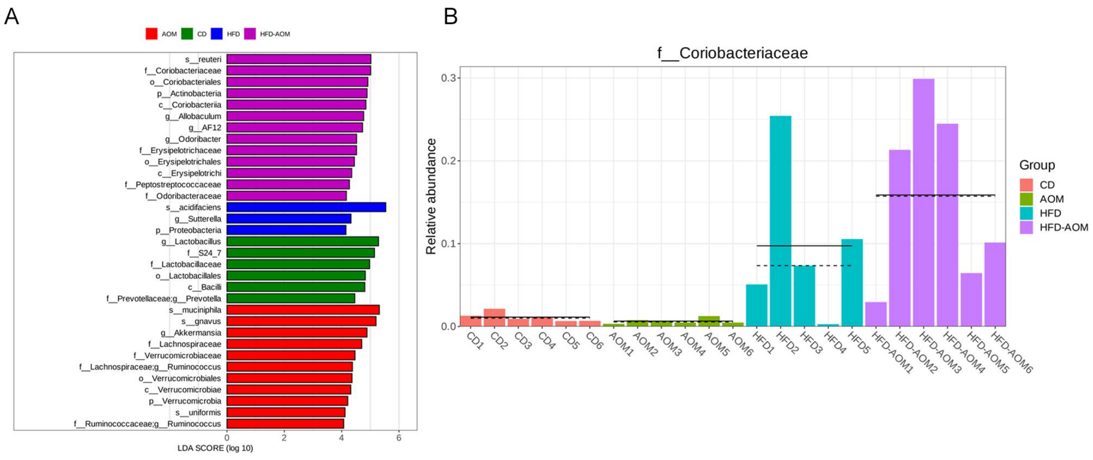


**Supplementary Fig. 1** LEfSe analysis results of faecal microbiota of CD(n = 6) , CD-AOM(n = 6) , HFD (n = 5), and HFD-AOM (n = 6) mice at 20 weeks by 16S rRNA sequencing. **A.** LDA value distribution bar chart. The LDA value distribution histogram shows species with LDA scores greater than the set value (default set to 4). **B.** Comparison of relative abundance of each biomarker in each group.

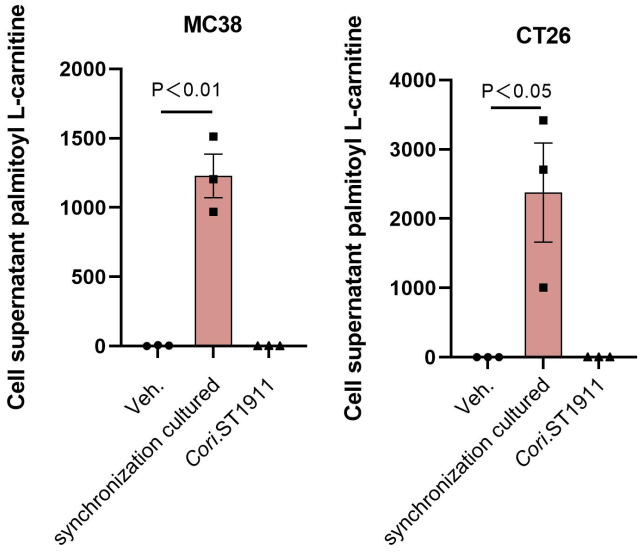


**Supplementary Fig. 2** Cell culture supernatant palmitoyl L-carnitine concentration following treatment with vehicle or *Cori.*ST1911 (n = 3). Data are represented as mean ± SEM, analysed using the two-tailed Student's t-test.

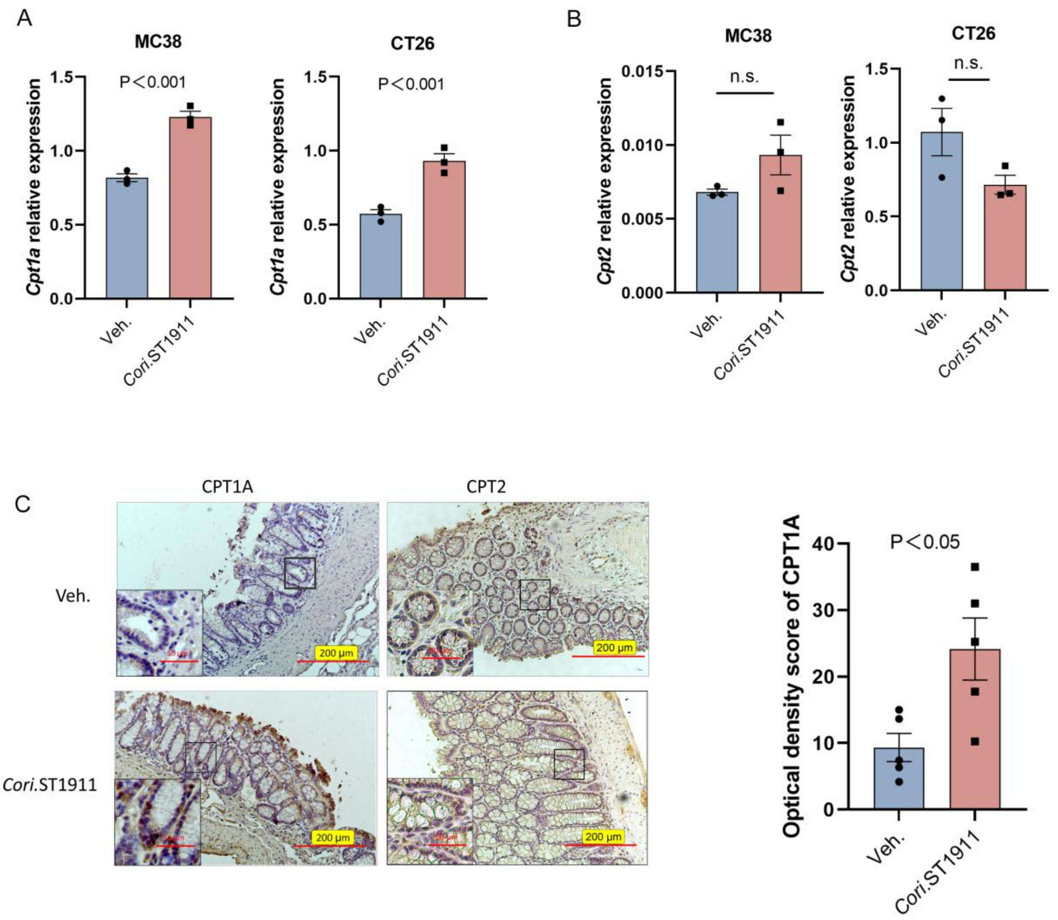


**Supplementary Fig. 3 A-B.** Quantitative PCR analysis of CPT1A and CPT2 mRNA expression in cell lines treated with vehicle or *Cori.*ST1911 (n = 3). Data are represented as mean ± SEM, analysed using the two-tailed Student's t-test; n.s., no significance. **C.** Representative images of CPT1A and CPT2 expression and quantitative analysis of CPT1A in colon tissues of *Cori.*ST1911 and GAM broth control group mice. Scale bars, 200 μm. Data are represented as mean ± SEM, analysed using two-tailed Student's t-test ; n.s., no significance.

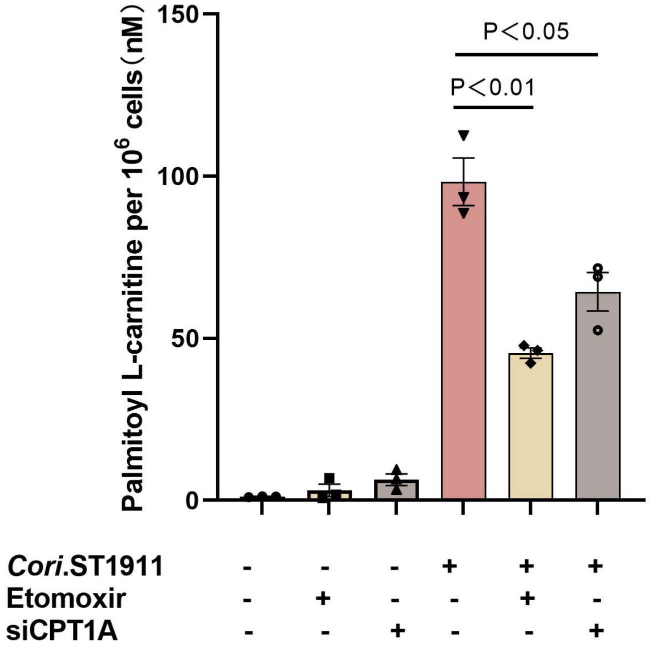


**Supplementary Fig. 4** Intracellular palmitoyl L-carnitine concentration following treatment with vehicle or *Cori.*ST1911 combined with siCPT1A (48 h) or etomoxir (100 μM) (n = 3). Data are represented as mean ± SEM; analysed using one way ANOVA; n.s., no significance.

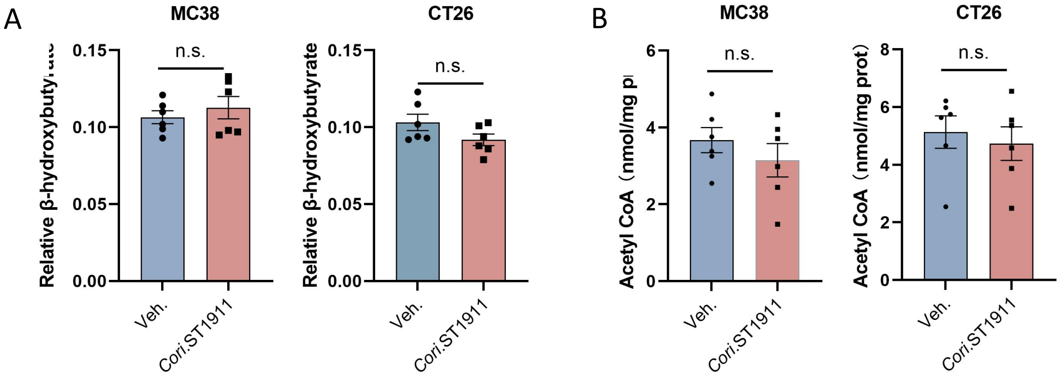


**Supplementary Fig. 5 A.** Intracellular β-hydroxybutyrate and **B.** Acetyl CoA concentration following treatment with *Cori.*ST1911 (n = 6). Data are represented as mean ± SEM; analysed using the two-tailed Student's t-test; n.s., no significance.

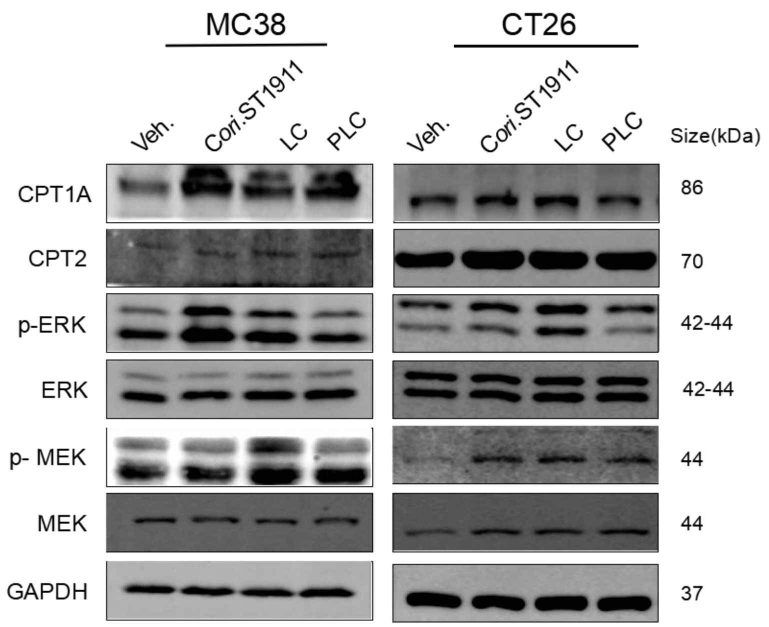


**Supplementary Fig. 6** Representative western blots showing the effect of *Cori.*ST1911 (MOI=100), LC (1 mM), and PLC (75 μM) on CPT1A and MAPK-related gene expression in cell lines MC38 and CT26.

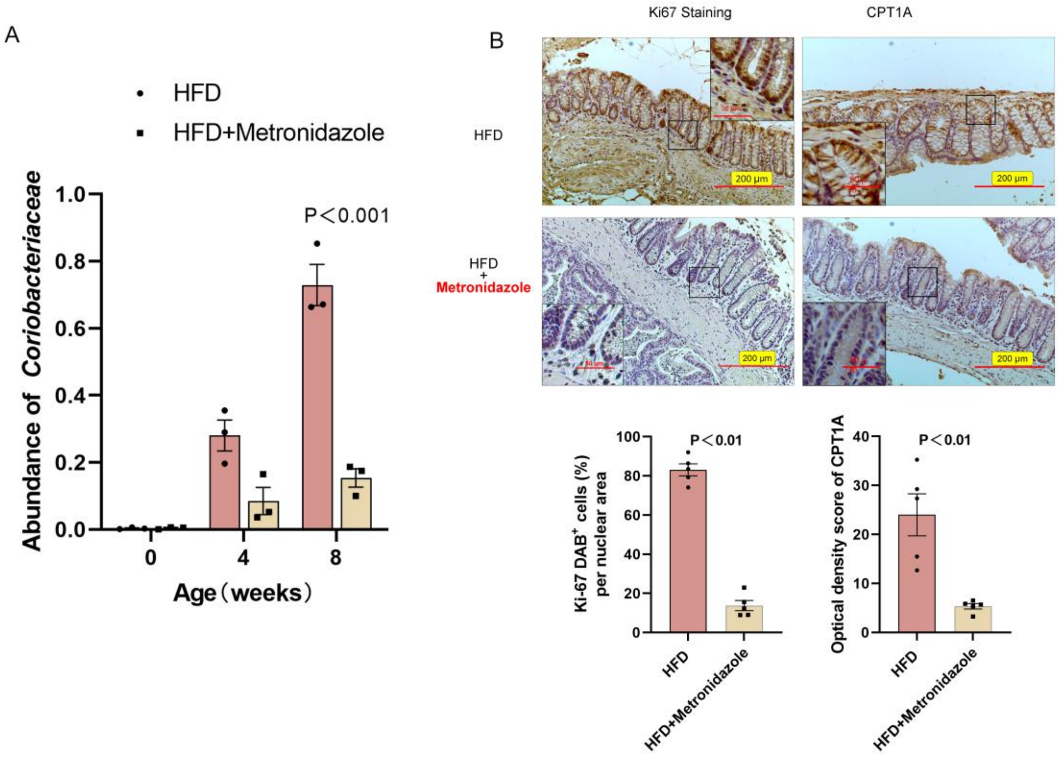


**Supplementary Fig. 7 A.** *Coriobacteriaceae* abundance in the stool of C57BL/6J AOM mice fed a HFD and a HFD combined with metronidazole, evaluated using qPCR and normalised to universal bacterial primers by targeting 16S rRNA genes (n = 3). **B.** Representative images of IHC of tissue nuclear Ki67 and CPT1A staining of the HFD group and the HFD combined with metronidazole mice with quantitative analysis of CPT1A and nuclear Ki67$^{+}$ cells. Scale bars, 200 µm. Data are represented as mean ± SEM, analysed using two-tailed Student's t-test.

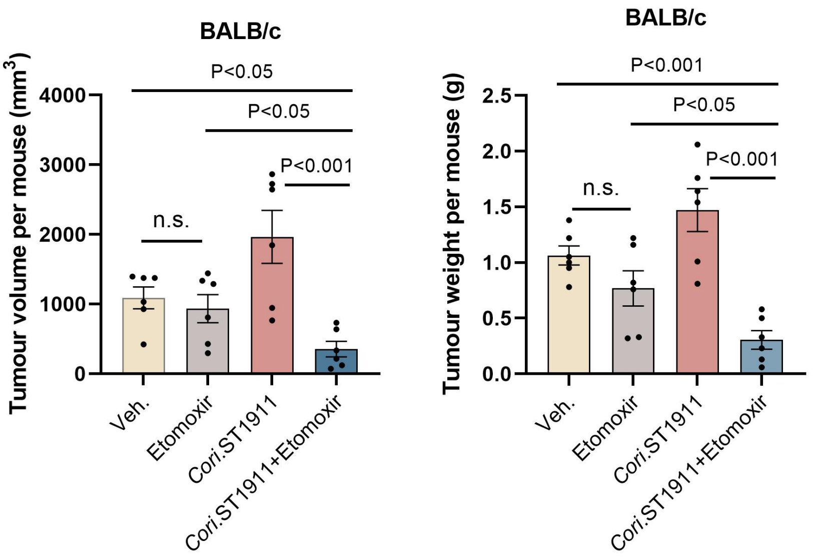


**Supplementary Fig. 8** Tumour weight and volume in subcutaneous transplanted tumour model BALB/c mice gavaged with *Cori.*ST1911 or *Cori.*ST1911+etomoxir (n = 6). Data are represented as mean ± SEM, analysed using one way ANOVA; n.s., no significance.

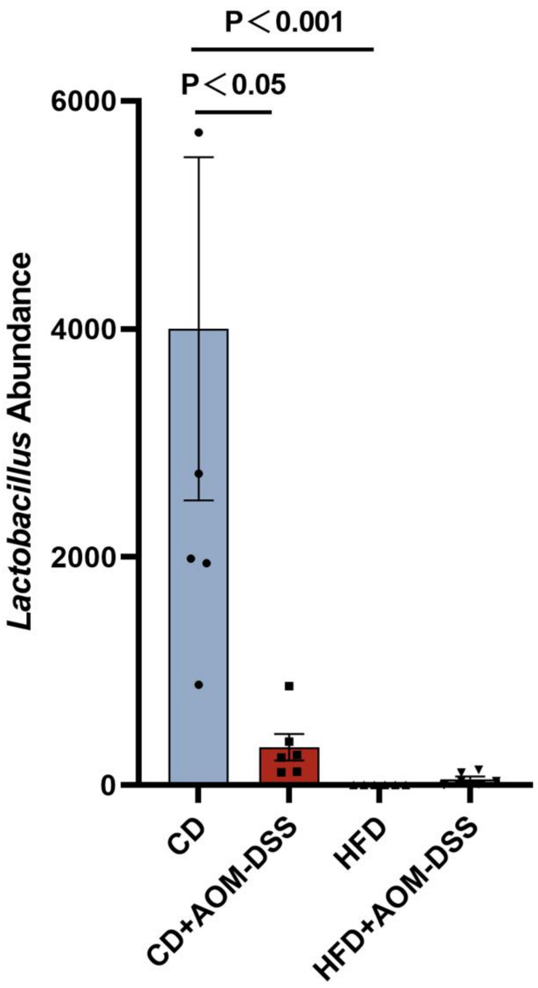


**Supplementary Fig. 9** *Lactobacillus* abundance in the stool of CD- or HFD-fed C57BL/6J AOM mice at 20 weeks, determined using qPCR, normalised to universal bacterial primers by targeting 16S rRNA genes (n = 5). Data are represented as mean ± SEM, analysed using one way ANOVA.

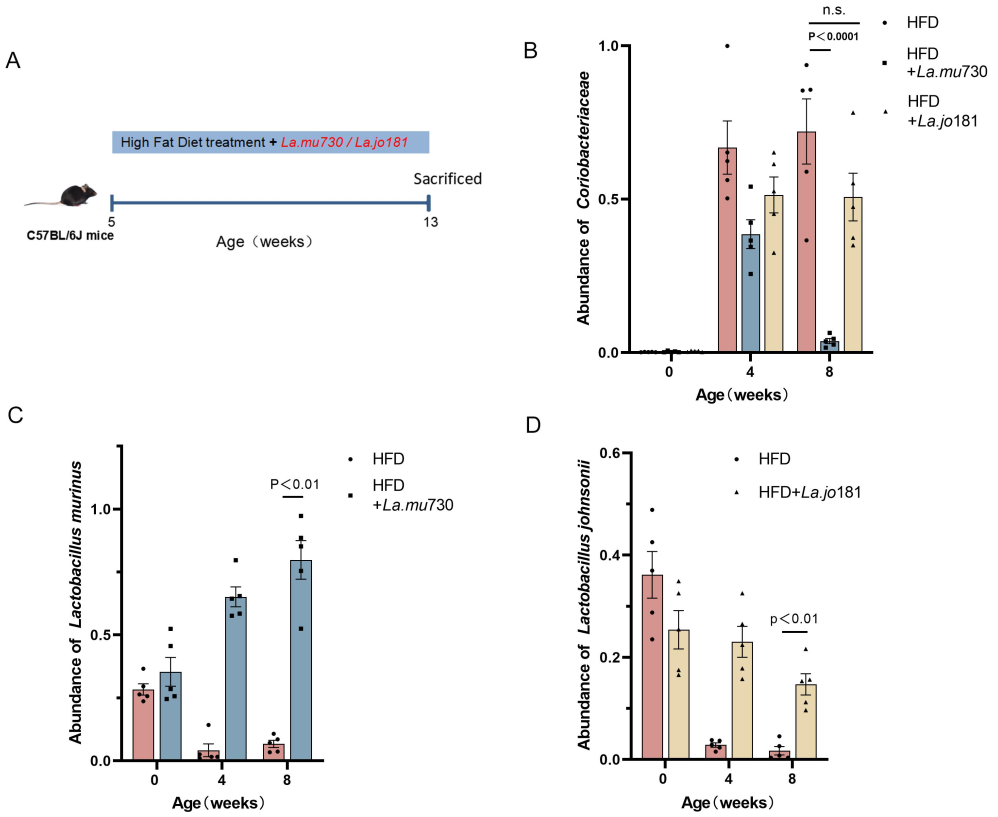


**Supplementary Fig. 10 A.** Schematic diagram of the experimental procedure of HFD-fed mice gavaged with *La.mu*730 or *La.jo*181 ($1×10^8$ CFU, every 2 days) for 8 weeks (n = 5). **B-D.** The abundance of *Coriobacteriaceae, Lactobacillus murinus* and *Lactobacillus johnsonii* in the stool of HFD-fed mice gavaged with *La.mu*730 or *La.jo*181 determined using qPCR, normalised to universal bacterial primers by targeting 16S rRNA genes (n = 5). Data are represented as mean ± SEM, analysed using one way ANOVA; n.s., no significance.

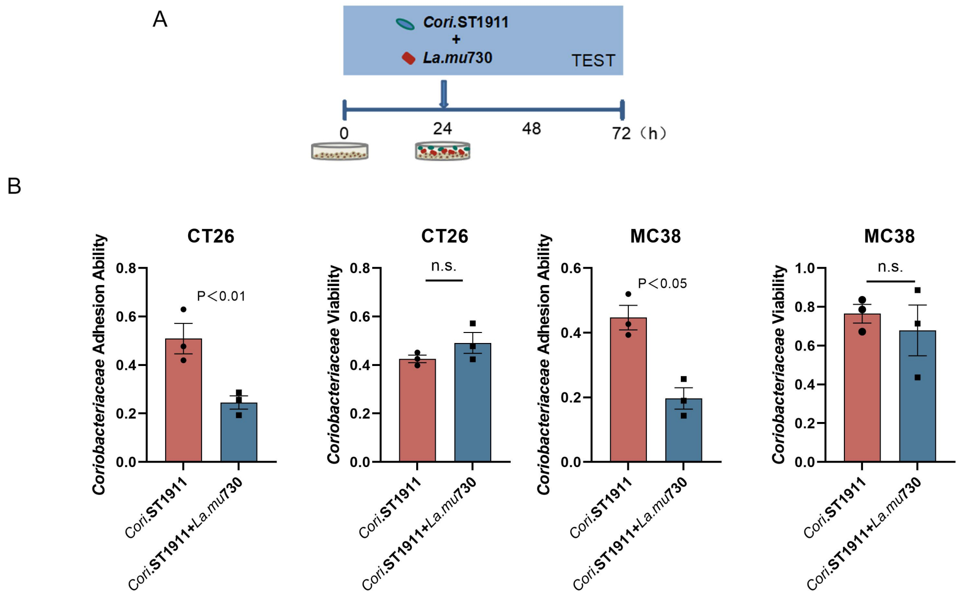


**Supplementary Fig. 11** Schematic diagram of the experimental procedure of *Lactobacillus* and *Coriobacteriaceae* co-cultured with cell lines for 48h (MOI = 100). **B.** Adhesion ability and viability of *Cori.*ST1911 in the co-culture system. Data are represented as mean ± SEM, analysed using two-tailed Student's t-test; n.s., no significance.

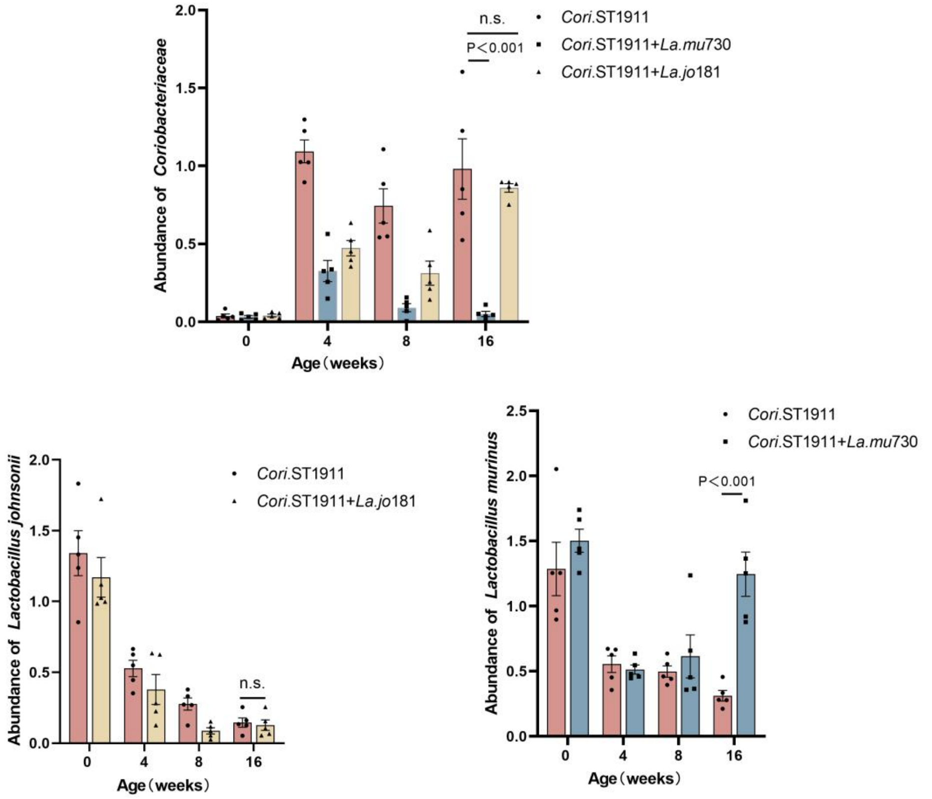


**Supplementary Fig. 12** *Coriobacteriaceae, Lactobacillus murinus* and *Lactobacillus johnsonii* abundance in the stool of C57BL/6J AOM mice gavaged with *Cori.*ST1911 combined with *La.mu*730 or *La.jo*181, determined using qPCR, normalised to universal bacterial primers by targeting 16S rRNA genes (n = 5). Data are represented as mean ± SEM, analysed using one way ANOVA; n.s., no significance.

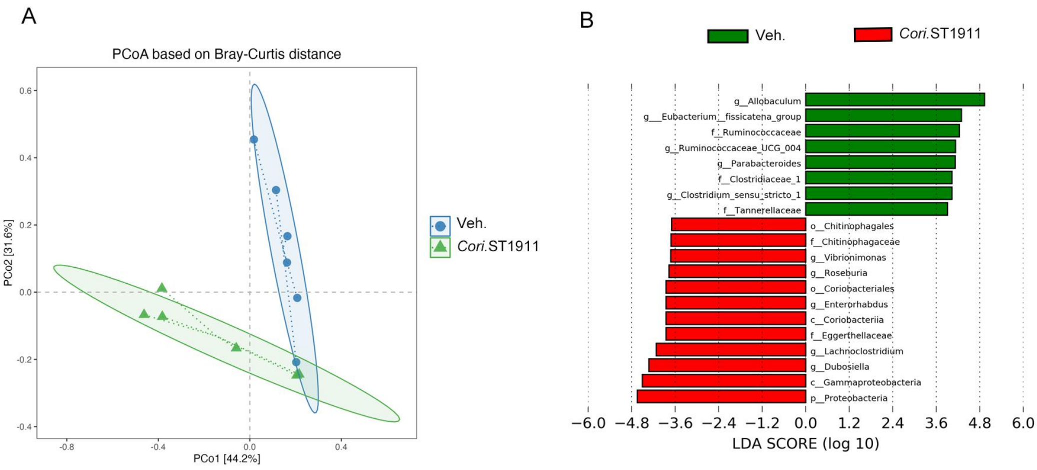


**Supplementary Fig. 13** 16S rRNA sequencing of faecal microbiota of vehicle- or *Cori.*ST1911-gavaged C57BL/6J AOM mice (n = 5) at 20 weeks. **A.** Unifrac PCoA of microbiota in the *Cori.*ST1911 and vehicle groups. **B.** Comparative analysis of species abundance in the *Cori.*ST1911 and vehicle groups using LEfSe and Wilcoxon rank sum test. The vertical axis represents the relative abundance of different species, the horizontal axis represents grouping information.

**Supplementary Table 1. Criterion of antibiotics resistance and Sensitivity of *Cori.*ST1911 to antibiotics by using Kirby-Bauer (K-B) method**

| Antibiotic | Content(μg) | The Sensitivity rang（mm） | | | The inhibition zone diameter（mm） | Antibiotic sensitivity |
|---|---|---|---|---|---|---|
| | | R | I | S | | |
| Aminoglycosides | | | | | | |
| Gentamicin | 10 | ≤12 | 13~14 | ≥15 | 16 | S |
| Neomycin | 30 | ≤12 | 13~16 | ≥17 | 18 | S |
| Glycopeptides | | | | | | |
| Vancomycin | 30 | ≤14 | 15~16 | ≥17 | 18 | S |
| β-Lactam | | | | | | |
| Ampicillin | 10 | ≤13 | 14~16 | ≥17 | 25 | S |
| Nitroimidazoles | | | | | | |
| Metronidazole | 5 | ≤12 | 13-15 | ≥16 | 34 | S |

**Supplementary Table 2. The ingredients of GAM**

| The ingredients of the culture medium of GAM (Gifu Anaerobic Medium) | |
|---|---|
| Ingredients | Contents |
| Proteose peptone | 15g/L |
| Pancreatic casein peptone | 10.0g/L |
| Soybean peptone | 3.0g/L |
| Yeast extract | 5.0g/L |
| Beef meal | 2.0g/L |
| Digest serum powder | 13.5g/L |
| Beef liver extract | 1.2g/L |
| Glucose | 3.0g/L |
| Potassium dihydrogen phosphate | 2.5g/L |
| Sodium chloride | 3.0g/L |
| Soluble starch | 0.3g/L |
| L-cysteine | 0.3g/L |
| Sodium thioglycollate | 0.15g/L |
| pH 7.2 | |

**Supplementary Table 3.** Diet Formulas

| **Diet Formulas** | **Rodent Diet with 10% Kcal% Fat(CD)** | | **Rodent Diet with 60% Kcal% Fat（HFD)** | |
|---|---|---|---|---|
| | **gm** | **kcal** | **gm** | **kcal** |
| Casein | 200 | 800 | 200 | 800 |
| L-cystine | 3 | 12 | 3 | 12 |
| Corn starch | 506.2 | 2024.8 | 0 | 0 |
| Maltodextrin | 125 | 500 | 125 | 500 |
| Sucrose | 68.8 | 275.2 | 68.8 | 275.2 |
| Cellulose | 50 | 0 | 50 | 0 |
| Soybean oil | 25 | 225 | 25 | 225 |
| Lard | 20 | 180 | 245 | 2205 |
| Mineral assemblage | 10 | 0 | 10 | 0 |
| Calcium hydrogen phosphate | 13 | 0 | 13 | 0 |
| Calcium carbonate | 5.5 | 0 | 5.5 | 0 |
| Potassium citrate | 16.5 | 0 | 16.5 | 0 |
| Compound vitamin | 10 | 40 | 10 | 40 |
| Choline Bitartrate | 2 | 0 | 2 | 0 |
| **Total** | **1055** | **4057** | **773.8** | **4057** |
| | gm% | kcal% | gm% | kcal% |
| Protein | 19.2 | 20 | 26.2 | 20 |
| Carbohydrate | 67.3 | 70 | 26.3 | 20 |
| Fat | 4.3 | 10 | 34.9 | 60 |

Supplementary Table 5 : **List of primers**

| Gene/bacterial | Forward(5-3’) | Reverse(5-3’) |
|---|---|---|
| **16S ribosomal RNA(rRNA)** | 27F-AGAGTTTGATCMTGGCTCAG | 1492R-CGGTTACCTTGTTACGACTT |
| **16S ribosomal RNA(rRNA)** | 8F-AGAGTTTGATCCTGGCTCAG | 336R-CTGCTGCCTCCCGTAGGAGT |
| Universal Bacterial | CGGCAACGAGCGCAACCC | CCATTGTAGCACGTGTGTAGCC |
| ***Coriobacteriaceae*** | AACGAGAAGCACCTCTTCGG | TCAACCCGGCTACCCATAGA |
| ***Lactobacillus sp.*** | AGCAGTAGGGAATCT TCCA | CACCGCTACACATGGAG |
| ***Lactobacillus murinus*** | CCAAGGCAATGATGCGTAGC | GTACTCCCCAGGCGGAATG |
| ***Lactobacillus johnsonii*** | ACTAGATACAAGCGAGCGGC | CCACCGCTACACATGGAGTT |
| **MUC2** | GGCTCGGAACTCCAGAAAGAA G | CTCGGCAGTCAGACGCAAAG |
| **CPT1A** | TTTGAATCGGCTCCTAATGG | CCCAAGTATCCACAGGGTCA |
| **CPT1B** | TTCAACACTACACGCATCCC | GCCCTCATAGAGCCAGACC |
| **CPT1C** | TATGCAGTCGCCCTTCCT | ACATCAATCAGGTGTGTCTGCT |
| **CPT2** | AGCAGTGCTCTAAAGGCTGG | GTTGGGATCTTCCGCTCACA |
| **CRAT** | CTGTCTCTGGTTCAGGGAGC | CTTAGAAGAACTGCCCGCCA |
| **CROT** | CGACGCAGAGGGACAAGAG | ACCACGAAGAAGTGCTCAGG |
| **CACT** | ATGTAGGTGAAAGGTCGGCG | ACAAGTTGGGGGCAATCCAA |
| **OCTN2** | TACGTGGCAGCATTTGTCCT | GAGAGATGAGCCATCGTGGG |

Supplementary Table 6 **Metabolite profiling of the serum from HFD and CD-fed mice by LC-MS/MS**

| Index | Compounds | VIP | Fold_Change | Log2FC |
|---|---|---|---|---|
| MEDN0005 | L-Threonine | 0.637956389 | 0.928033061 | -0.107751892 |
| MEDN0007 | L-Arginine | 0.068630606 | 0.996322355 | -0.0053155 |
| MEDN0010 | L-Citrulline | 1.100810759 | 0.827564635 | -0.273056099 |
| MEDN0011 | L-Glutamic Acid | 0.956852108 | 1.14339986 | 0.193330019 |
| MEDN0013 | L-Isoleucine | 1.387085023 | 0.697660857 | -0.519402202 |
| MEDN0015 | L-Phenylalanine | 1.365210226 | 0.66818626 | -0.581677777 |
| MEDN0017 | L-Pyroglutamic Acid | 1.33532406 | 1.38490226 | 0.469784161 |
| MEDN0018 | L-Serine | 1.198820798 | 0.647594071 | -0.626838318 |
| MEDN0025 | 3-Hydroxy-3-Methylpentane-1,5-Dioic Acid | 0.396776962 | 1.032010454 | 0.045457585 |
| MEDN0030 | 5-Hydroxy-L-Tryptophan | 0.21895112 | 1.035171688 | 0.049870065 |
| MEDN0032 | Allantoin | 0.769414277 | 1.128644373 | 0.174590976 |
| MEDN0041 | Hexanoyl Glycine | 1.842832597 | 2.434903991 | 1.283864888 |
| MEDN0042 | L-Asparagine Anhydrous | 1.152957926 | 0.624274537 | -0.679747472 |
| MEDN0047 | L-Homocitrulline | 1.983425212 | 0.718604161 | -0.476730807 |
| MEDN0052 | N6-Acetyl-L-Lysine | 0.26204892 | 1.03102006 | 0.044072403 |
| MEDN0053 | N-Acetylaspartate | 1.339844767 | 1.418892278 | 0.504765065 |
| MEDN0056 | N-Acetyl-L-Leucine | 0.586924349 | 0.911996995 | -0.132899024 |
| MEDN0057 | N-Acetyl-L-Tyrosine | 0.168589961 | 0.935108174 | -0.096794828 |
| MEDN0060 | N-Isovaleroylglycine | 0.419518247 | 0.918216318 | -0.123094024 |
| MEDN0061 | N-Phenylacetylglycine | 0.969258911 | 0.685747209 | -0.544251249 |
| MEDN0063 | Nα-Acetyl-L-Arginine | 1.596497668 | 1.266512694 | 0.340861537 |
| MEDN0065 | O-Phospho-L-Serine | 1.619315536 | 0.829380281 | -0.269894347 |
| MEDN0066 | Phenylacetyl-L-Glutami | 0.103688346 | 1.046303011 | 0.065300719 |

| | | | | |
|---|---|---|---|---|
| | ne | | | |
| MEDN0071 | S-Sulfo-L-Cysteine | 0.705528265 | 0.311050795 | -1.684777902 |
| MEDN0075 | N-Acetylphenylalanine | 1.168331549 | 0.618698455 | -0.692691664 |
| MEDN0085 | Benzoylformic Acid | 1.605428358 | 0.713895514 | -0.486215159 |
| MEDN0086 | Terephthalic Acid | 0.289037067 | 0.963106513 | -0.054232736 |
| MEDN0089 | 2,5-Dihydroxy Benzoic Acid | 0.743270348 | 1.320947481 | 0.401573108 |
| MEDN0090 | 2-Methoxybenzoic Acid | 0.290617178 | 0.938228267 | -0.091989128 |
| MEDN0092 | 3,4-Dihydroxybenzeneacetic Acid | 0.325047378 | 1.025437662 | 0.036239791 |
| MEDN0093 | 3-Hydroxyanthranilic Acid | 0.696741608 | 0.849688366 | -0.234994284 |
| MEDN0099 | 4-Pyridoxic Acid | 0.360458344 | 0.928441097 | -0.107117711 |
| MEDN0105 | Taurocholic Acid | 0.23625367 | 0.551259293 | -0.859197023 |
| MEDN0106 | Taurochenodesoxycholic Acid | 1.066284761 | 0.05695372 | -4.134066104 |
| MEDN0109 | Hododeoxycholic acid | 0.969642894 | 1.737809575 | 0.797270004 |
| MEDN0112 | Glycocholic Acid | 0.576146411 | 0.116604438 | -3.100305394 |
| MEDN0120 | Dulcitol | 1.760963462 | 0.693109874 | -0.528844023 |
| MEDN0132 | Biotin | 0.822646362 | 1.282355143 | 0.358795865 |
| MEDN0140 | Xanthine | 0.136351588 | 1.749674744 | 0.807086757 |
| MEDN0141 | 2'-Deoxyuridine | 1.525760945 | 1.912365622 | 0.935358376 |
| MEDN0145 | 5'-Deoxy-5'-(Methylthio) Adenosine | 0.47990702 | 1.033146906 | 0.04704541 |
| MEDN0146 | 5-Hydroxymethyluracil | 1.147958634 | 1.274063602 | 0.349437299 |
| MEDN0147 | 5-Methylcytosine | 1.032506688 | 0.9218444 | -0.11740484 |
| MEDN0159 | Flavin Adenine Dinucleotide | 0.664572355 | 1.171005937 | 0.227748391 |
| MEDN0165 | Inosine 5'-Monophosphate | 0.929081043 | 0.831596962 | -0.266043607 |

| | | | | |
|---|---|---|---|---|
| MEDN0168 | Thymidine | 1.541067903 | 1.740469665 | 0.799476669 |
| MEDN0198 | Citric Acid | 1.519620405 | 1.74736886 | 0.805184186 |
| MEDN0201 | Succinic Acid | 0.589970762 | 1.051161914 | 0.071984909 |
| MEDN0202 | A-Ketoglutaric Acid | 0.768991178 | 1.119407376 | 0.162735159 |
| MEDN0204 | Pyruvic Acid | 0.494188877 | 0.925785397 | -0.111250288 |
| MEDN0206 | Citramalic Acid | 0.747090717 | 1.10417378 | 0.142967248 |
| MEDN0211 | D-Arabitol | 1.517993835 | 1.675294006 | 0.744414304 |
| MEDN0213 | D-Sorbitol | 0.390896329 | 0.957150942 | -0.06318164 |
| MEDN0220 | D-Glucose | 0.044476019 | 1.030224596 | 0.04295889 |
| MEDN0224 | D-Trehalose | 0.4223453 | 1.01913156 | 0.027340301 |
| MEDN0227 | D-Glucose 6-Phosphate | 0.483803659 | 0.939233922 | -0.09044358 |
| MEDN0229 | Lactose | 0.4223453 | 1.01913156 | 0.027340301 |
| MEDN0230 | Lactulose | 0.4223453 | 1.01913156 | 0.027340301 |
| MEDN0231 | L-Fucose | 1.165472058 | 0.736508996 | -0.441224948 |
| MEDN0232 | L-Rhamnose | 1.165472058 | 0.736508996 | -0.441224948 |
| MEDN0233 | Maltose | 0.4223453 | 1.01913156 | 0.027340301 |
| MEDN0237 | D-Glucoronic Acid | 0.945594255 | 1.18876677 | 0.249465693 |
| MEDN0238 | D-Glyceric Acid | 1.862134601 | 0.520182115 | -0.942911299 |
| MEDN0240 | L-Gulonolactone | 0.80117634 | 0.82251124 | -0.281892701 |
| MEDN0241 | Vitamin D3 | 1.340547452 | 0.797487087 | -0.326466936 |
| MEDN0245 | Pantothenate | 0.870141891 | 0.764126105 | -0.388117347 |
| MEDN0250 | Riboflavin | 1.147587733 | 0.940684507 | -0.088217152 |
| MEDN0279 | 2-Aminoethanesulfinic Acid | 0.780694827 | 1.18571234 | 0.245754048 |
| MEDN0283 | 2-Hydroxybutanoic Acid | 1.771145132 | 0.6019903 | -0.732187854 |
| MEDN0284 | 2-Hydroxyisocaproic Acid | 0.530935108 | 0.867592584 | -0.204910373 |
| MEDN0285 | 2-Methylsuccinic Acid | 0.147045637 | 0.931774784 | -0.101946807 |

| | | | | |
|---|---|---|---|---|
| MEDN0290 | 3-Hydroxy-3-Methyl Butyric Acid | 0.726980303 | 1.118553943 | 0.161634833 |
| MEDN0292 | 3-Hydroxybutyrate | 1.274178343 | 0.702257704 | -0.509927549 |
| MEDN0293 | 3-Hydroxypropanoic Acid | 0.547005628 | 1.055573624 | 0.078027207 |
| MEDN0294 | 3-Methylcrotonyl Glycine | 0.426688242 | 1.093617567 | 0.129108323 |
| MEDN0297 | 4-Hydroxy-2-Oxoglutari c Acid | 0.627055009 | 0.902089722 | -0.148657163 |
| MEDN0298 | 4-Oxopentanoate | 0.67598996 | 1.112033777 | 0.153200609 |
| MEDN0299 | Adipic Acid | 0.007329551 | 0.983654384 | -0.023776595 |
| MEDN0300 | Azelaic Acid | 0.929223234 | 0.708646559 | -0.49686184 |
| MEDN0301 | Caffeic Acid | 0.242065997 | 0.883854467 | -0.178119256 |
| MEDN0304 | Cinnamic Acid | 1.086620001 | 0.650544987 | -0.620279268 |
| MEDN0305 | Creatine | 0.637150662 | 0.869421543 | -0.201872251 |
| MEDN0314 | Glutaric Acid | 0.147045637 | 0.931774784 | -0.101946807 |
| MEDN0316 | Guanidinoethyl Sulfonate | 0.921117782 | 1.136645114 | 0.184781883 |
| MEDN0317 | Hippuric Acid | 0.277878884 | 0.881785266 | -0.181500724 |
| MEDN0319 | 4-Hydroxy-3-methoxyp henylacetic acid | 0.136981381 | 0.985727298 | -0.020739515 |
| MEDN0320 | Hydrocinnamic Acid | 0.788479389 | 0.496155087 | -1.01113695 |
| MEDN0322 | Kinurenine | 0.045619689 | 1.009086477 | 0.013049816 |
| MEDN0325 | L-Lactic Acid | 1.420800968 | 0.938771395 | -0.091154212 |
| MEDN0327 | L-Dihydroorotic Acid | 1.269849653 | 1.109556873 | 0.149983618 |
| MEDN0328 | L-Homoserine | 0.637956389 | 0.928033061 | -0.107751892 |
| MEDN0333 | Malonicacid | 1.362969436 | 0.689487106 | -0.536404523 |
| MEDN0334 | Mandelic Acid | 0.263184082 | 1.199979094 | 0.263009272 |
| MEDN0335 | Methylmalonic Acid | 0.589970762 | 1.051161914 | 0.071984909 |
| MEDN0336 | N'-Formylkynurenine | 1.011526844 | 0.824579293 | -0.278269862 |

| | | | | |
|---|---|---|---|---|
| MEDN0338 | Phenyllactate(Pla) | 1.262931278 | 0.610681224 | -0.711508607 |
| MEDN0339 | Phenylpyruvic Acid | 0.985766457 | 0.56042636 | -0.835403279 |
| MEDN0340 | Pyrrole-2-Carboxylic Acid | 1.693678189 | 0.609842727 | -0.713490862 |
| MEDN0341 | Rosmarinic Acid | 0.161030516 | 1.010588844 | 0.015196159 |
| MEDN0342 | Sebacate | 1.361987428 | 1.965301053 | 0.974750328 |
| MEDN0343 | Shikimic Acid | 0.319062054 | 1.247250523 | 0.318751274 |
| MEDN0344 | Subericacid | 0.283850434 | 0.915848572 | -0.126819014 |
| MEDN0348 | TXB2 [9α,11,15S-trihydroxythromba-5Z,13E-dien-1-oic acid] | 0.24196111 | 1.046303545 | 0.065301455 |
| MEDN0350 | (±)15-HETE [(±)15-hydroxy-5Z,8Z,11Z,13E-eicosatetraenoic acid] | 1.302716904 | 0.675270317 | -0.566462952 |
| MEDN0360 | Lysopg 18:1 | 0.281290878 | 1.047367625 | 0.066767915 |
| MEDN0362 | Lysope 18:1 | 1.117713078 | 1.195406147 | 0.257500867 |
| MEDN0364 | Lysope 18:0 | 0.468479017 | 0.922416967 | -0.116509044 |
| MEDN0366 | Lysope 16:0 | 0.45618075 | 1.06454902 | 0.090242385 |
| MEDN0368 | Lysope 14:0 | 0.530285483 | 1.109741415 | 0.150223548 |
| MEDN0370 | Lysopa 18:0 | 0.798654513 | 0.836554665 | -0.257468279 |
| MEDN0372 | Lysopa 16:0 | 0.403011011 | 1.073288884 | 0.10203844 |
| MEDN0374 | 9-HpODE | 1.597714976 | 0.43703408 | -1.194182309 |
| MEDN0375 | 13-HOTrE [13S-hydroxy-9Z,11E,15Z-octadecatrienoic acid] | 0.541743992 | 0.808320119 | -0.307001338 |
| MEDN0376 | 9,10-DiHOME [(±)9,10-dihydroxy-12Z-octadecenoic acid] | 1.683896904 | 0.418303677 | -1.257377415 |

| | | | | |
|---|---|---|---|---|
| MEDN0378 | γ-Linolenic Acid(C18:3N6) | 0.075981219 | 0.955630217 | -0.065475623 |
| MEDN0380 | Palmitoleic Acid(C16:1) | 0.435345306 | 1.078791618 | 0.109416217 |
| MEDN0381 | Hexadecanoic Acid(C16:0) | 0.519065331 | 0.897719398 | -0.155663525 |
| MEDN0383 | Linoleic Acid(C18:2N6C) | 0.592852754 | 1.098267727 | 0.135229786 |
| MEDN0385 | Dodecanoic Acid(C12:0) | 0.497101885 | 0.945882508 | -0.080267103 |
| MEDN0388 | Elaidic Acid(C18:1N9T) | 0.648331929 | 0.86378991 | -0.211247631 |
| MEDN0390 | EPA [5Z,8Z,11Z,14Z,17Z-eicosapentaenoic acid] | 0.364382529 | 1.120287844 | 0.163869462 |
| MEDN0391 | DHA [4Z,7Z,10Z,13Z,16Z,19Z-docosahexaenoic acid] | 1.101474598 | 1.298934713 | 0.377328919 |
| MEDN0395 | Cis-11,14,17-Eicosatrienoic Acid(C20:3) | 0.618099797 | 1.026366415 | 0.037545868 |
| MEDN0398 | AA [5Z,8Z,11Z,14Z-eicosatetraenoic acid] | 0.547750119 | 1.166408184 | 0.222072747 |
| MEDN0399 | Arachidic Acid(C20:0) | 0.345259416 | 0.870302706 | -0.200410812 |
| MEDN0400 | A-Linolenic Acid(C18:3N3) | 0.075981219 | 0.955630217 | -0.065475623 |
| MEDN0412 | Biopterin | 1.176975625 | 1.362711963 | 0.446480651 |
| MEDN0413 | Ethylmalonate | 0.147045637 | 0.931774784 | -0.101946807 |
| MEDN0416 | Ureidoisobutyric Acid | 0.818585704 | 1.260797845 | 0.334336974 |
| MEDN0417 | (Rs)-Mevalonic Acid | 0.607678215 | 0.877814103 | -0.188012646 |
| MEDN0428 | N6-Succinyl Adenosine | 0.185763843 | 1.023045216 | 0.032869911 |
| MEDN0431 | 2-(Dimethylamino)Guanosine | 1.457582434 | 1.490196579 | 0.575502656 |
| MEDN0432 | 5-Hydroxyhexanoic Acid | 0.094423392 | 1.007804534 | 0.011215852 |

| | | | | |
|---|---|---|---|---|
| MEDN0434 | B-Pseudouridine | 0.895924558 | 1.176613584 | 0.234640597 |
| MEDN0439 | N-Acetylthreonine | 0.324431292 | 0.953714178 | -0.06837113 |
| MEDN0444 | Hypoxanthine-9-β-D-Arabinofuranoside | 0.03033108 | 0.672069691 | -0.573317252 |
| MEDN0452 | 2'-Deoxycytidine-5'-Monophosphate | 1.070947914 | 1.418522739 | 0.504389277 |
| MEDN0461 | D-Sedoheptuiose 7-Phosphate | 0.271642948 | 1.007682308 | 0.011040872 |
| MEDN0463 | D-Fructose 6-Phosphate-Disodium Salt | 1.255511584 | 0.64075807 | -0.642148352 |
| MEDN0470 | Oxoadipic Acid | 1.496451825 | 1.280342447 | 0.356529733 |
| MEDN0478 | Aminomalonic Acid | 0.579207577 | 1.04226497 | 0.059722094 |
| MEDN0481 | 2,4-Dihydroxy Benzoic Acid | 0.743270348 | 1.320947481 | 0.401573108 |
| MEDN0483 | 3-Methyl-2-Oxobutanoic Acid | 0.67598996 | 1.112033777 | 0.153200609 |
| MEDN0485 | D-Fructose-1,6-Biphosphate-Trisodium Salt | 0.520533528 | 0.87355099 | -0.195036177 |
| MEDN0491 | 9-HOTrE [9S-hydroxy-10E,12Z,15Z-octadecatrienoic acid] | 1.824261825 | 0.418951872 | -1.255143574 |
| MEDN0494 | 8,15-Dihete | 0.465274499 | 1.066285736 | 0.092594093 |
| MEDN0496 | N-Acetylmethionine | 0.491400111 | 0.941731293 | -0.086612624 |
| MEDN0498 | Ribulose-5-Phosphate | 0.316931963 | 0.938344734 | -0.09181005 |
| MEDN0499 | Argininosuccinic acid | 1.455487148 | 1.700225422 | 0.765726037 |
| MEDN0501 | Acetyl Tryptophan | 0.094801743 | 1.00174223 | 0.002511319 |
| MEDN0502 | 2-Deoxyribose 1-Phosphate | 0.347497849 | 0.953651714 | -0.068465623 |
| MEDN0506 | N-Acetylglucosamine 1-Phosphate | 0.564459752 | 1.072654504 | 0.101185467 |

| | | | | |
|---|---|---|---|---|
| MEDN0523 | Indolelactic acid | 0.594025065 | 1.148861517 | 0.200204907 |
| MEDN0528 | (3-Methoxy-4-hydroxyphenyl)ethylene glycol sulfate | 0.189371414 | 0.954802482 | -0.066725778 |
| MEDN0530 | Pimelic acid | 0.447632263 | 0.87187815 | -0.197801572 |
| MEDN0533 | Xanthosine | 1.24336741 | 1.319578777 | 0.40007748 |
| MEDN0536 | estrone 3-sulfate | 1.059915303 | 0.615168296 | -0.700946942 |
| MEDN0546 | 13,14-dihydro-15-keto-PGD2 | 0.878644365 | 0.387198674 | -1.368854084 |
| MEDN0551 | Indoleacrylic acid | 0.76011013 | 1.073789643 | 0.102711395 |
| MEDN0555 | Hydroxyphenyllactic acid | 0.549980306 | 0.877340579 | -0.188791096 |
| MEDN0556 | Cinnamoylglycine | 1.70730365 | 1.395258354 | 0.480532285 |
| MEDN0561 | N-Acetylaspartylglutamic acid | 1.064257848 | 1.441186611 | 0.527257154 |
| MEDN0568 | 2-Methylguanosine | 1.327630371 | 1.416663333 | 0.502496946 |
| MEDN0570 | L-Erythrulose | 0.313347144 | 1.028920851 | 0.041132008 |
| MEDN0579 | N-lactoyl-phenylalanine | 1.633582505 | 0.462422243 | -1.112717301 |
| MEDN0581 | Imidazoleacetic acid | 0.760101584 | 0.788959452 | -0.34197694 |
| MEDN0588 | Glycerol 3-phosphate | 1.131127486 | 1.363949269 | 0.447789986 |
| MEDN0593 | D-Malic acid | 1.436170333 | 1.476946436 | 0.562617506 |
| MEDN0594 | (E)-3-(3-hydroxyphenyl)prop-2-enoic acid | 1.778331726 | 0.267131292 | -1.90437911 |
| MEDN0604 | Tetradecanedioic acid | 0.711389581 | 0.741783786 | -0.430929361 |
| MEDN0606 | 5-Methoxytryptophol | 0.290554719 | 0.961344833 | -0.056874079 |
| MEDN0611 | 6β-hydroxytestosterone | 0.184601237 | 1.007939327 | 0.011408799 |
| MEDN0615 | Carbamoyl phosphate | 0.527841457 | 1.082401532 | 0.114235787 |
| MEDN0616 | O-Acetyl-L-serine | 0.632111717 | 1.116609041 | 0.159124142 |
| MEDN0621 | Indoxylsulfuric acid | 1.134366841 | 0.680102015 | -0.556176928 |

| | | | | |
|---|---|---|---|---|
| MEDN0623 | Salicyluric acid | 0.273407399 | 1.091832451 | 0.126751483 |
| MEDN0632 | N-Oleoyl Glycine | 0.38006194 | 0.957452318 | -0.062727453 |
| MEDN0651 | Succinic anhydride | 1.694625208 | 1.250421476 | 0.322414462 |
| MEDN0656 | Indoleacetaldehyde | 1.074488761 | 0.879220077 | -0.185703765 |
| MEDN0657 | D-Xylulose 5-phosphate | 0.316931963 | 0.938344734 | -0.09181005 |
| MEDN0658 | Hexadecanedioic acid | 0.381939416 | 0.800088879 | -0.321767823 |
| MEDN0661 | 2-hydroxy-2-(4-hydroxy-3-methoxyphenyl)acetic acid | 1.831090364 | 1.693242904 | 0.759788949 |
| MEDN0662 | Gamma-Glu-Leu | 1.397284051 | 1.315437934 | 0.39554318 |
| MEDN0673 | Isopropyl myristate | 0.870958032 | 1.040706329 | 0.05756302 |
| MEDN0678 | Ethylsalicylate | 0.463719281 | 0.935138189 | -0.096748522 |
| MEDN0679 | 3-Hydroxy-2-methyl-4H-pyran-4-one | 0.06868234 | 0.907482694 | -0.140057964 |
| MEDN0682 | Octadecanamide | 0.257238655 | 1.036510711 | 0.051735026 |
| MEDN0691 | Maltitol | 1.543414292 | 0.582180767 | -0.780460917 |
| MEDN0704 | Oxaloacetic acid | 0.652736131 | 0.850216975 | -0.234097032 |
| MEDN0720 | N-(2-Methylbenzoyl)glycine | 0.969258911 | 0.685747209 | -0.544251249 |
| MEDN0725 | 2,4-Hexadienoic acid | 1.930729385 | 0.598841361 | -0.739754225 |
| MEDN0729 | Phenoxyacetic acid | 0.767079764 | 0.845254309 | -0.242542628 |
| MEDN0732 | Ricinoleic acid | 1.820278806 | 0.467286692 | -1.097620144 |
| MEDN0736 | 2-(Methylthio)ethanol | 0.282614282 | 1.028504034 | 0.040547452 |
| MEDN0741 | Methanesulfonic acid | 1.041741401 | 0.804197413 | -0.3143784 |
| MEDN0745 | Propylparaben | 0.490055472 | 0.948514231 | -0.076258676 |
| MEDN0746 | Butylparaben | 0.01761938 | 0.983677119 | -0.02374325 |
| MEDN0748 | Methylparaben | 1.287877444 | 1.540182936 | 0.623101718 |
| MEDN0750 | (±)12-HEPE [(±)-12-hydroxy-5Z,8Z,10E,14Z,17Z-eicosapenta | 1.044100666 | 1.48918062 | 0.574518747 |

| | | | | |
|---|---|---|---|---|
| | enoic acid] | | | |
| MEDN0751 | (±)12-HETE [(±)12-hydroxy-5Z,8Z,10E,14Z-eicosatetraenoic acid] | 0.124176555 | 0.983444419 | -0.024084576 |
| MEDN0752 | (±)15-HEPE [(±)-15-hydroxy-5Z,8Z,11Z,13E,17Z-eicosapentaenoic acid] | 1.044100666 | 1.48918062 | 0.574518747 |
| MEDN0753 | (±)16-HETE [(±)16-hydroxy-5Z,8Z,11Z,14Z-eicosatetraenoic acid] | 0.175196137 | 0.967784966 | -0.047241568 |
| MEDN0754 | (±)17-HDHA [(±)17-hydroxy-4Z,7Z,10Z,13Z,15E,19Z-docosahexaenoic acid] | 1.115730107 | 1.563904729 | 0.645152628 |
| MEDN0755 | (±)17-HETE [(±)17-hydroxy-5Z,8Z,11Z,14Z-eicosatetraenoic acid] | 0.247162165 | 0.955981192 | -0.064945861 |
| MEDN0756 | (±)18-HEPE [(±)-18-hydroxy-5Z,8Z,11Z,14Z,16E-eicosapentaenoic acid] | 1.044100666 | 1.48918062 | 0.574518747 |
| MEDN0757 | (±)18-HETE [(±)18-hydroxy-5Z,8Z,11Z,14Z-eicosatetraenoic acid] | 0.247162165 | 0.955981192 | -0.064945861 |
| MEDN0758 | (±)4-HDHA [(±)4-hydroxy-5E,7Z,10Z,13Z,16Z,19Z-docosahexaenoic acid] | 0.494035494 | 1.133837973 | 0.181214492 |
| MEDN0759 | (±)5-HEPE [(±)-5-hydroxy-6E,8Z,11Z,14Z,17Z-eicosapentae | 1.105379522 | 0.631724448 | -0.662632689 |

| | | | | |
|---|---|---|---|---|
| | noic acid] | | | |
| MEDN0760 | (±)5-HETE [(±)5-hydroxy-6E,8Z,11Z,14Z-eicosatetraenoic acid] | 0.366879996 | 0.907203877 | -0.14050129 |
| MEDN0763 | (±)9-HETE [(±)-9-hydroxy-5Z,7E,11Z,14Z-eicosatetraenoic acid] | 0.366879996 | 0.907203877 | -0.14050129 |
| MEDN0765 | 11,12-EET [(±)11,(12)-epoxy-5Z,8Z,14Z-eicosatrienoic acid] | 0.446451017 | 0.914694214 | -0.12863857 |
| MEDN0767 | 12,13-EpOME [(±)12(13)epoxy-9Z-octadecenoic acid] | 1.786195346 | 0.228249185 | -2.131318389 |
| MEDN0768 | 13-oxoODE [13-oxo-9Z,11E-octadecadienoic acid] | 1.8572583 | 0.514797783 | -0.957922254 |
| MEDN0769 | 14(S)-HDHA [14S-hydroxy-4Z,7Z,10Z,12E,16Z,19Z-docosahexaenoic acid] | 1.277907159 | 1.612503612 | 0.689302392 |
| MEDN0777 | 5,6-DiHETrE [(±)5,6-dihydroxy-8Z,11Z,14Z-eicosatrienoic acid] | 1.309587285 | 0.663199388 | -0.59248542 |
| MEDN0782 | 8,9-EET [(±)8,9-epoxy-5Z,11Z,14Z-eicosatrienoic acid] | 0.610055241 | 0.996987298 | -0.004352971 |
| MEDN0783 | 9,10-EpOME [(±)9,10-epoxy-12Z-octadecenoic acid] | 1.786195346 | 0.228249185 | -2.131318389 |
| MEDN0784 | 9-oxoODE [9-oxo-10E,12Z-octadecadienoic acid] | 2.042364449 | 0.368457454 | -1.440430053 |

| | | | | |
|---|---|---|---|---|
| MEDN0793 | Prostaglandin E2 | 0.616032934 | 1.098753611 | 0.135867906 |
| MEDN0795 | PGF2α [9α,11α,15S-trihydroxy-prosta-5Z,13E-dien-1-oic acid] | 0.449549376 | 1.10710172 | 0.146787782 |
| MEDN0806 | Nα-Acetyl-L-glutamine | 0.140626922 | 1.060777762 | 0.085122437 |
| MEDN0807 | Myristic acid | 0.872897324 | 0.808961724 | -0.305856652 |
| MEDN0808 | Myoinositol | 0.575459368 | 0.949253513 | -0.075134662 |
| MEDN0816 | 4-Hydroxyhippurate | 0.227422427 | 1.067228195 | 0.093868687 |
| MEDN0818 | N-acetyl-beta-alanine | 0.377355951 | 1.036506333 | 0.051728931 |
| MEDN0827 | N-acetylornithine | 0.183928242 | 0.971905533 | -0.041112001 |
| MEDN0837 | N-Methyl-L-Glutamate | 1.84755095 | 1.353657065 | 0.436862294 |
| MEDN0841 | 3-Ureidopropionate | 0.747933537 | 0.868229971 | -0.20385087 |
| MEDN0843 | N-Amidino-L-Aspartate | 0.47246599 | 1.052037196 | 0.073185714 |
| MEDN0846 | Lumichrome | 0.196309239 | 1.001342464 | 0.001935467 |
| MEDN0849 | Cortisol 21-Acetate | 0.203518517 | 0.994816576 | -0.007497548 |
| MEDN0851 | Indole-3-Pyruvic Acid | 0.822029664 | 0.794010706 | -0.332769634 |
| MEDN0854 | Mono-Methyl Glutarate | 0.347647863 | 1.041982485 | 0.059331027 |
| MEDN1003 | Inosine | 0.03033108 | 0.672069691 | -0.573317252 |
| MEDN1005 | Phosphoenolpyruvate | 0.152567948 | 0.996354408 | -0.005269088 |
| MEDN1009 | 3-hydroxyphenylacetic acid | 1.525330025 | 0.62682937 | -0.673855316 |
| MEDN1024 | D-piperidine acid | 1.376672032 | 1.380184 | 0.464860613 |
| MEDN1035 | 13(R)-HODE | 1.298486703 | 0.657971496 | -0.603903009 |
| MEDN1055 | D-Calcium Pantothenate | 0.736338805 | 0.54107218 | -0.88610703 |
| MEDN1056 | Iminodiacetic acid | 0.860281664 | 1.143187182 | 0.193061646 |
| MEDN1059 | Taurodeoxycholic acid | 0.949289412 | 0.024953866 | -5.324592829 |
| MEDN1063 | Homo-Gamma-Linolenic Acid | 0.082773446 | 0.968033475 | -0.046871157 |

| | | | | |
|---|---|---|---|---|
| MEDN1069 | 9,12-octadecadienoic acid | 0.938808693 | 0.656054744 | -0.608111891 |
| MEDN1077 | Alpha-Mercholic Acid | 1.44354359 | 2.352449359 | 1.234163666 |
| MEDN1078 | Cis-9,10-epoxystearic acid | 1.513749497 | 0.401858449 | -1.31524068 |
| MEDN1079 | (4E,7E,10Z,13E,16E,19E)-docosa-4,7,10,13,16,19-hexanoic acid | 1.101474598 | 1.298934713 | 0.377328919 |
| MEDN1081 | 12,13-DiHOME | 1.683896904 | 0.418303677 | -1.257377415 |
| MEDN1085 | Glutathione | 1.39694435 | 1.342119264 | 0.424512879 |
| MEDN1104 | 7-ketolithocholic acid | 0.132047233 | 0.477030494 | -1.067846602 |
| MEDN1108 | 1,6-anhydro-β-D-glucose | 0.269435347 | 0.972472033 | -0.040271334 |
| MEDN1112 | 16-Hydroxyhexadecanoic acid | 0.746349771 | 0.910970161 | -0.134524295 |
| MEDN1113 | 12-ketolithocholic acid | 0.132047233 | 0.477030494 | -1.067846602 |
| MEDN1126 | Gamma-Mercholic Acid | 1.38164736 | 1.707093385 | 0.771541982 |
| MEDN1129 | Erythrose | 0.313347144 | 1.028920851 | 0.041132008 |
| MEDN1141 | Orthocholic acid | 0.133929435 | 0.468449635 | -1.094034149 |
| MEDN1156 | 8,11-icosadienoic acid | 0.612577594 | 0.839115862 | -0.253058068 |
| MEDN1159 | cis-1-Pentadecenoic Acid(C15: 1) | 0.966655227 | 1.069418911 | 0.096827093 |
| MEDN1167 | 4-hydroxybenzoic acid | 0.492855237 | 0.876521789 | -0.19013814 |
| MEDN1264 | PysoPE 22:4(2n isomer1) | 0.615623733 | 0.80152913 | -0.319173142 |
| MEDN1265 | PysoPE 22:4 | 0.615623733 | 0.80152913 | -0.319173142 |
| MEDN1266 | PysoPE 22:5(2n isomer3) | 0.444659515 | 0.804603971 | -0.313649236 |
| MEDN1267 | PysoPE 22:5(2n isomer2) | 0.444659515 | 0.804603971 | -0.313649236 |

| | | | | |
|---|---|---|---|---|
| MEDN1268 | PysoPE 22:5(2n isomer1) | 0.444659515 | 0.804603971 | -0.313649236 |
| MEDN1269 | PysoPE 22:6(2n isomer1) | 0.418795541 | 1.073966759 | 0.10294934 |
| MEDN1270 | PysoPE 22:6 | 0.418795541 | 1.073966759 | 0.10294934 |
| MEDN1271 | PysoPE 20:2(2n isomer1) | 1.905032238 | 1.78112713 | 0.832790494 |
| MEDN1272 | PysoPE 20:2 | 1.905032238 | 1.78112713 | 0.832790494 |
| MEDN1273 | PysoPE 20:3(2n isomer1) | 1.139832374 | 1.395628735 | 0.480915207 |
| MEDN1274 | PysoPE 20:3 | 1.139832374 | 1.395628735 | 0.480915207 |
| MEDN1276 | PysoPE 20:4(2n isomer1) | 0.243640786 | 1.030742011 | 0.043683279 |
| MEDN1277 | PysoPE 20:5(2n isomer1) | 1.185529478 | 1.282649245 | 0.359126703 |
| MEDN1278 | PysoPE 20:5 | 1.185529478 | 1.282649245 | 0.359126703 |
| MEDN1279 | PysoPE 18:0(2n isomer) | 0.468479017 | 0.922416967 | -0.116509044 |
| MEDN1282 | PysoPE 18:2 | 0.025361799 | 1.003972991 | 0.005720459 |
| MEDN1283 | PysoPE 18:3(2n isomer) | 0.047337254 | 1.007790354 | 0.011195553 |
| MEDN1284 | PysoPE 18:3 | 0.12269638 | 0.976473424 | -0.034347316 |
| MEDN1285 | PysoPE 16:0(2n isomer) | 0.45618075 | 1.06454902 | 0.090242385 |
| MEDN1287 | PysoPE 16:1 | 1.58454665 | 1.621581195 | 0.697401263 |
| MEDN1295 | 15-methyl palmitic acid | 1.650633596 | 0.531884885 | -0.910814056 |
| MEDP0002 | D-Homocysteine | 0.489443046 | 0.840556298 | -0.250583644 |
| MEDP0006 | Glycine | 0.52838423 | 1.042441356 | 0.059966225 |
| MEDP0007 | L-Cystine | 0.751561946 | 1.069914473 | 0.097495475 |
| MEDP0009 | L-Tyrosine | 0.959043161 | 0.910249439 | -0.135666149 |
| MEDP0011 | L-Lysine | 0.963821403 | 1.095465579 | 0.131544153 |
| MEDP0012 | L-Ornithine | 0.356567892 | 0.932710153 | -0.100499273 |
| MEDP0013 | L-Alanine | 0.489154431 | 0.726201866 | -0.461557457 |

| | | | | |
|---|---|---|---|---|
| MEDP0014 | L-Aspartic Acid | 0.071310782 | 1.011609083 | 0.016651896 |
| MEDP0017 | L-Histidine | 1.394786134 | 0.626159767 | -0.675397281 |
| MEDP0020 | L-Methionine | 1.770728726 | 0.773186289 | -0.371112041 |
| MEDP0022 | L-Proline | 0.856136112 | 0.856436721 | -0.223581441 |
| MEDP0025 | L-Tryptophan | 1.114474952 | 0.856278345 | -0.223848253 |
| MEDP0026 | L-Valine | 1.214843137 | 0.817212071 | -0.29121758 |
| MEDP0028 | (5-L-Glutamyl)-L-Amino Acid | 0.649713312 | 1.095371299 | 0.131419985 |
| MEDP0034 | 3-N-Methyl-L-Histidine | 0.348141101 | 0.958743223 | -0.06078362 |
| MEDP0036 | 5-Oxoproline | 1.882002252 | 1.817353552 | 0.861839112 |
| MEDP0037 | Asp-phe | 1.696903283 | 0.50706693 | -0.979751908 |
| MEDP0039 | Betaine | 1.112177567 | 1.228531543 | 0.2969349 |
| MEDP0043 | Glutathione Oxidized | 1.320020886 | 1.268495167 | 0.343118023 |
| MEDP0044 | Glutathione Reducedform | 0.501045993 | 0.744914503 | -0.424853244 |
| MEDP0047 | Histamine | 1.529017432 | 0.681866931 | -0.552437875 |
| MEDP0050 | L-Carnosine | 0.025985582 | 1.035930341 | 0.050926996 |
| MEDP0051 | L-Cystathionine | 0.72255471 | 0.912370687 | -0.132308 |
| MEDP0053 | L-Dopa | 1.652502019 | 0.594673418 | -0.749830507 |
| MEDP0054 | L-Glutamine | 0.562177816 | 1.048152607 | 0.067848783 |
| MEDP0058 | L-Saccharopine | 1.769538456 | 1.175210853 | 0.232919624 |
| MEDP0060 | Methionine Sulfoxide | 1.398654915 | 1.201610837 | 0.264969729 |
| MEDP0061 | N,N-Dimethylglycine | 0.495642718 | 1.044844616 | 0.063288409 |
| MEDP0065 | N-Acetyl-L-Glutamic Acid | 0.910908225 | 0.934885831 | -0.097137902 |
| MEDP0067 | N-Acetylmannosamine | 1.515746211 | 1.269093659 | 0.343798544 |
| MEDP0071 | N-Glycyl-L-Leucine | 1.423527199 | 1.346361311 | 0.429065625 |
| MEDP0074 | N-Propionylglycine | 1.1103983 | 1.471539527 | 0.557326295 |
| MEDP0079 | S-(5-Adenosy)-L-Homoc | 1.482432531 | 0.700718558 | -0.513092989 |

| | | | | |
|---|---|---|---|---|
| | ysteine | | | |
| MEDP0081 | Serotonin | 0.788477343 | 0.887294114 | -0.172515697 |
| MEDP0083 | Trans-4-Hydroxy-L-Proline | 0.464164993 | 0.955750427 | -0.065294156 |
| MEDP0084 | Trimethylamine N-Oxide | 1.528978303 | 1.563861528 | 0.645112775 |
| MEDP0086 | Urea | 1.2566715 | 0.881369078 | -0.182181813 |
| MEDP0087 | L-Alanyl-L-Lysine | 1.556304259 | 1.463774225 | 0.549693047 |
| MEDP0089 | N-Acetylhistamine | 0.043244245 | 1.017108383 | 0.024473421 |
| MEDP0101 | P-Coumaric Acid | 1.01960673 | 0.811641941 | -0.301084677 |
| MEDP0111 | 3-(4-Hydroxyphenyl)-Propionic Acid | 1.197350282 | 0.779382007 | -0.359597469 |
| MEDP0115 | 1,4-Dihydro-1-Methyl-4-Oxo-3-Pyridinecarboxamide | 0.401608199 | 1.085159061 | 0.117906526 |
| MEDP0120 | Theobromine | 0.615314488 | 1.476995046 | 0.562664988 |
| MEDP0125 | Choline | 0.173143746 | 1.01365306 | 0.01956395 |
| MEDP0145 | 1,7-Dimethylxanthine | 1.211592182 | 0.736235338 | -0.441761097 |
| MEDP0147 | 1-Methylhistidine | 0.913167417 | 0.767152037 | -0.382415571 |
| MEDP0151 | 2-Hydroxy-6-Aminopurine | 1.294719374 | 0.77464895 | -0.368385427 |
| MEDP0152 | 3'-Aenylic Acid | 0.392777455 | 0.925378529 | -0.111884469 |
| MEDP0156 | 5-Methyluridine | 0.505085354 | 0.946409565 | -0.079463441 |
| MEDP0157 | 7-Methylxanthine | 0.246198476 | 1.023061099 | 0.032892308 |
| MEDP0159 | Adenine | 0.730204797 | 0.861887753 | -0.214428101 |
| MEDP0160 | Adenosine | 0.576783429 | 0.906620439 | -0.141429409 |
| MEDP0161 | Adenosine 5'-Diphosphate | 1.505945429 | 0.534337329 | -0.904177286 |
| MEDP0163 | Cytidine | 0.754691026 | 1.26791441 | 0.34245736 |
| MEDP0164 | Cytidine-5-Monophosp | 1.008460639 | 1.072993742 | 0.101641662 |

| | | | | |
|---|---|---|---|---|
| | hate | | | |
| MEDP0165 | Cytosine | 0.372045533 | 1.050056527 | 0.070466994 |
| MEDP0167 | Guanosine | 0.804860493 | 0.326155225 | -1.616369355 |
| MEDP0174 | Purine | 1.046353162 | 0.900613623 | -0.151019794 |
| MEDP0177 | Thymine | 1.416469098 | 1.675304869 | 0.744423658 |
| MEDP0178 | Uracil | 1.489357253 | 1.538454282 | 0.621481572 |
| MEDP0179 | Uridine | 1.518942754 | 1.29367594 | 0.371476274 |
| MEDP0184 | 3,3',5-Triiodo-L-Thyronine | 0.861541071 | 1.294258926 | 0.372126268 |
| MEDP0186 | L-Thyroxine | 1.644284608 | 1.425916902 | 0.511889909 |
| MEDP0188 | Norepinephrine | 1.123824711 | 0.780736171 | -0.357092985 |
| MEDP0214 | Tryptamine | 1.417900674 | 0.825105741 | -0.277349076 |
| MEDP0218 | D-Mannitol | 1.459136038 | 0.687571008 | -0.540419381 |
| MEDP0224 | D-Fructose | 0.484592036 | 0.94502129 | -0.081581263 |
| MEDP0227 | D-Mannose | 0.738060444 | 0.896365263 | -0.157841355 |
| MEDP0228 | D-Melezitose | 0.426561184 | 1.09318687 | 0.128540038 |
| MEDP0232 | N-Acetyl-D-Glucosamine | 1.515746211 | 1.269093659 | 0.343798544 |
| MEDP0241 | Orotic Acid | 1.561871051 | 1.738557487 | 0.797890772 |
| MEDP0242 | Nicotinamide | 1.567434503 | 0.736460101 | -0.441320729 |
| MEDP0243 | 4-Oxoretinol | 0.448157031 | 1.063901007 | 0.089363918 |
| MEDP0244 | All-Trans-13,14-Dihydroretinol | 1.307247593 | 0.602073455 | -0.731988584 |
| MEDP0245 | Nicotinamide-N-Oxide | 0.280121586 | 0.925322168 | -0.111972342 |
| MEDP0246 | Nicotinic Acid | 0.877306936 | 0.841247819 | -0.249397235 |
| MEDP0251 | Trigonelline | 1.720245399 | 0.709240828 | -0.495652506 |
| MEDP0271 | 3-Indolepropionic Acid | 1.567573294 | 0.516400051 | -0.953438952 |
| MEDP0272 | 5-Hydroxyindole-3-Acetic Acid | 1.635258588 | 0.328152817 | -1.607560276 |

| | | | | |
|---|---|---|---|---|
| MEDP0275 | Indole-3-Acetic Acid | 1.0967152 | 0.851963007 | -0.231137306 |
| MEDP0287 | 2-Aminoethanesulfonic Acid | 0.263735512 | 0.960845056 | -0.057624291 |
| MEDP0289 | 3,4,5-Trimethoxycinnamic Acid | 0.285204354 | 0.769594743 | -0.377829151 |
| MEDP0296 | 4-Guanidinobutyric Acid | 1.051861309 | 0.820017445 | -0.286273493 |
| MEDP0297 | 5-Aminovaleric Acid | 0.230260032 | 1.257260178 | 0.330283233 |
| MEDP0298 | 6-Aminocaproic Acid | 1.152271508 | 0.900140662 | -0.151777631 |
| MEDP0299 | 7-Methyluric Acid | 0.466811173 | 0.973078391 | -0.039372061 |
| MEDP0305 | Creatinine | 0.270424615 | 0.944724418 | -0.082034548 |
| MEDP0307 | Dl-2-Aminooctanoic Acid | 0.06830848 | 0.913024917 | -0.131273862 |
| MEDP0308 | Dodecanedioic Aicd | 1.03467844 | 0.83778436 | -0.255349142 |
| MEDP0313 | Guanidineacetic Acid | 0.566593961 | 1.439953066 | 0.526021789 |
| MEDP0319 | Kynurenic Acid | 0.024279255 | 0.943563424 | -0.0838086 |
| MEDP0325 | Maleic Acid | 0.943240792 | 0.819704074 | -0.286824927 |
| MEDP0333 | Uric acid | 1.007401477 | 0.89855896 | -0.154314923 |
| MEDP0336 | Lysopc 14:0 | 0.390451975 | 1.047406287 | 0.066821169 |
| MEDP0338 | Lysopc 16:0 | 0.319396506 | 1.023744737 | 0.033856036 |
| MEDP0340 | Lysopc 16:1 | 1.113427946 | 1.183757938 | 0.2433741 |
| MEDP0342 | Lysopc 18:0 | 0.196310474 | 0.976858665 | -0.033778251 |
| MEDP0344 | Lysopc 18:1 | 0.69281853 | 1.10656715 | 0.146091001 |
| MEDP0346 | Lysopc 18:2 | 0.414139416 | 1.017107478 | 0.024472138 |
| MEDP0350 | Lysopc 20:1 | 1.260869637 | 1.220098882 | 0.286998075 |
| MEDP0352 | Lysopc 20:2 | 1.573593526 | 1.337845325 | 0.419911328 |
| MEDP0357 | Urocanic Acid | 0.876868609 | 0.798674432 | -0.324320565 |
| MEDP0365 | Cotinine N-Oxide | 1.54816384 | 1.322251968 | 0.402997123 |
| MEDP0368 | Taurocholic Acid | 0.614800239 | 0.973076355 | -0.039375081 |

| | | | | |
|---|---|---|---|---|
| | Sodium Salt Hydrate | | | |
| MEDP0370 | 2-(Formylamino)Benzoic Acid | 1.31866991 | 0.605646801 | -0.723451402 |
| MEDP0372 | Uridine 5-Monophosphate | 0.989206004 | 1.32001313 | 0.40055228 |
| MEDP0373 | N-Acetylglycine | 0.712823759 | 1.108055712 | 0.148030421 |
| MEDP0379 | 3-Hydroxyhippuric Acid | 0.280166179 | 1.088556984 | 0.122416932 |
| MEDP0381 | 7-Methylguanine | 0.569576992 | 0.855078051 | -0.225871981 |
| MEDP0387 | L-Homoarginine | 0.222558865 | 1.019388051 | 0.027703348 |
| MEDP0389 | Pantetheine | 0.805720888 | 0.929898066 | -0.104855516 |
| MEDP0395 | DL-Pipecolic Acid | 1.888181598 | 1.789679343 | 0.839701122 |
| MEDP0403 | Deoxycytidine | 0.650934935 | 0.93003329 | -0.104645737 |
| MEDP0408 | 11-Cis-Retinol | 1.316535876 | 1.35822163 | 0.441718913 |
| MEDP0429 | Punicic Acid | 1.491867811 | 0.631429946 | -0.663305413 |
| MEDP0430 | 2-Aminoadipic Acid | 1.752465769 | 1.200566151 | 0.263714898 |
| MEDP0434 | Lysopc 17:0 | 0.067740848 | 1.002959714 | 0.004263658 |
| MEDP0435 | 6-Methylmercaptopurine | 1.404029651 | 0.957641599 | -0.062442271 |
| MEDP0438 | N-Formylmethionine | 0.644668835 | 0.895704664 | -0.158904977 |
| MEDP0441 | N-Caffeoyl Putrescine | 0.156048862 | 0.996445055 | -0.005137839 |
| MEDP0442 | Sn-Glycero-3-Phosphocholine | 0.908260449 | 1.20344068 | 0.26716503 |
| MEDP0453 | Indole | 1.182805385 | 0.847840645 | -0.238134965 |
| MEDP0455 | Dihydrojasmone | 1.215153827 | 0.781546454 | -0.355596467 |
| MEDP0457 | 5-Aminolevulinate | 0.787765386 | 0.901792616 | -0.149132398 |
| MEDP0494 | Lysopc 15:0 | 1.228800965 | 1.322836339 | 0.403634582 |
| MEDP0495 | Lysope 18:2 (2N Isomer) | 0.414139416 | 1.017107478 | 0.024472138 |
| MEDP0498 | Lysopc 18:3 | 0.317680261 | 1.014494839 | 0.020761526 |

| | | | | |
|---|---|---|---|---|
| MEDP0507 | 2-Hydroxycinnamic acid | 1.01960673 | 0.811641941 | -0.301084677 |
| MEDP0509 | trans-3-Indoleacrylic acid | 1.102323556 | 0.774923614 | -0.367873987 |
| MEDP0510 | Acetyl-L-carnitine | 0.185017373 | 0.996082135 | -0.005663387 |
| MEDP0511 | Jasmonic acid | 0.347363065 | 1.058822905 | 0.082461309 |
| MEDP0514 | Thiamine | 0.981477546 | 0.726647288 | -0.460672839 |
| MEDP0518 | DL-Stachydrine | 1.780155192 | 1.372087984 | 0.456372996 |
| MEDP0519 | L-Norleucine | 1.062773156 | 0.844768308 | -0.243372384 |
| MEDP0523 | DL-Carnitine | 0.68285406 | 0.92291693 | -0.115727295 |
| MEDP0525 | N-Acetyl-L-alanine | 1.457621909 | 0.813448711 | -0.29787671 |
| MEDP0528 | 5-Aminosalicylic Acid | 0.08029871 | 0.985184829 | -0.021533683 |
| MEDP0529 | Indole-3-acetamide | 0.16914488 | 0.934027688 | -0.098462777 |
| MEDP0530 | 15-deoxy-δ-12,14-PGJ2 | 1.157779493 | 0.84689355 | -0.239747453 |
| MEDP0535 | Triethyl phosphate | 0.278352727 | 1.00425249 | 0.006122038 |
| MEDP0540 | 7,8-dihydro-L-Biopterin | 1.760834206 | 1.549153024 | 0.631479659 |
| MEDP0550 | 18-Hydroxycorticosterone | 0.693698334 | 1.154143213 | 0.206822253 |
| MEDP0560 | Dihydrouracil | 0.247955002 | 0.814409748 | -0.296173265 |
| MEDP0575 | 5-amino-1-[3,4-dihydroxy-5-(hydroxymethyl)oxolan-2-yl]imidazole-4-carboxamide | 0.324899304 | 1.170291712 | 0.226868187 |
| MEDP0577 | Isobutyryl carnitine | 0.232432698 | 0.835287247 | -0.259655684 |
| MEDP0584 | Pterine | 0.625461324 | 0.844054031 | -0.244592741 |
| MEDP0585 | Stearidonic Acid | 1.222860627 | 1.144559315 | 0.19479223 |
| MEDP0587 | N-Alpha-acetyllysine | 0.695504812 | 0.872193768 | -0.197279412 |
| MEDP0611 | N-Acetyl-L-Histidine | 0.748707819 | 1.286681926 | 0.363655457 |
| MEDP0618 | 2-Methylbutyroylcarnitine | 0.044019079 | 0.896654767 | -0.157375474 |
| MEDP0627 | Isonicotinic acid | 1.480684903 | 0.735042367 | -0.444100687 |

| | | | | |
|---|---|---|---|---|
| MEDP0637 | L-phenylalanyl-L-proline | 1.168592118 | 1.343167154 | 0.425638856 |
| MEDP0638 | LysoPE(16:1(9Z)/0:0) | 1.385222001 | 1.520932815 | 0.604956426 |
| MEDP0654 | 2-Phenylacetamide | 0.378343292 | 0.95582781 | -0.065177351 |
| MEDP0658 | Ethyl dodecanoate | 0.240322598 | 1.049815697 | 0.070136074 |
| MEDP0677 | Butylamine | 0.110470598 | 0.946171967 | -0.079825677 |
| MEDP0685 | Methylcysteine | 0.863449948 | 0.820121061 | -0.286091209 |
| MEDP0692 | Triethylamine | 0.752687359 | 0.868992163 | -0.202584928 |
| MEDP0695 | Methyl tetradecanoate | 0.292683691 | 0.943824798 | -0.083409018 |
| MEDP0729 | Barbituric acid | 0.070866692 | 0.953644567 | -0.068476436 |
| MEDP0765 | 3-(Methylthio)-1-propanol | 0.331701032 | 1.153880175 | 0.206493415 |
| MEDP0777 | Diethyl malonate | 0.537621258 | 0.951156913 | -0.072244732 |
| MEDP0789 | 2-Pentyl-3-phenyl-2-propenal | 1.275620889 | 0.816860982 | -0.291837522 |
| MEDP0792 | Pyrrolidine | 1.385466424 | 0.766751017 | -0.38316992 |
| MEDP0821 | 6-Methylnicotinamide | 0.068554 | 0.818396179 | -0.289128686 |
| MEDP0831 | 1-Aminopropan-2-ol | 1.528978303 | 1.563861528 | 0.645112775 |
| MEDP0859 | Glycylphenylalanine | 1.131695603 | 1.394086855 | 0.479320448 |
| MEDP0868 | N-Methylalanine | 0.495642718 | 1.044844616 | 0.063288409 |
| MEDP0879 | N-Methyl-D-Aspartic Acid | 0.294453745 | 0.976281638 | -0.034630699 |
| MEDP0881 | Phosphocholine | 1.767601314 | 1.199212087 | 0.26208683 |
| MEDP0885 | Mesoxalate | 0.443413934 | 0.962887575 | -0.054560733 |
| MEDP0888 | 2'-Deoxycytidine 5'-Diphosphate | 0.617873601 | 1.167864966 | 0.223873473 |
| MEDP0889 | Cortisol | 1.24846236 | 0.396187768 | -1.335743758 |
| MEDP0891 | L-Tryptophanamide | 0.809394737 | 0.954545455 | -0.067114196 |
| MEDP0894 | 3-Methoxytyramine | 0.481104508 | 1.056715862 | 0.079587506 |

| | | | | |
|---|---|---|---|---|
| MEDP0898 | Pyridoxal | 1.116185128 | 0.75958844 | -0.396710146 |
| MEDP1001 | Delta-Hexalactone | 0.871885502 | 1.118080885 | 0.161024561 |
| MEDP1005 | β-Alanine | 0.489154431 | 0.726201866 | -0.461557457 |
| MEDP1015 | 5,6-Dihydroxyindole-2-Carboxylic Acid | 1.310008192 | 1.309421993 | 0.388930115 |
| MEDP1040 | N-(2-hydroxyethyl)stearamide | 0.301454554 | 0.900363823 | -0.151420005 |
| MEDP1045 | Biliverdin | 1.507551169 | 1.451183702 | 0.537230158 |
| MEDP1063 | Indole-5-carboxylic acid | 0.121616335 | 0.955428571 | -0.065780075 |
| MEDP1068 | 1,2-dioctanoyl PC | 1.35704996 | 1.473745145 | 0.559487061 |
| MEDP1070 | Glycyl-L-valine | 0.863814838 | 1.252562189 | 0.324882234 |
| MEDP1084 | Dodecylcarnitine | 2.02021453 | 1.358243302 | 0.441741933 |
| MEDP1096 | 1,2-dodecanoyl PC | 1.536352275 | 1.347230752 | 0.429996975 |
| MEDP1102 | Decanoyl L-Carnitine | 1.471225399 | 1.257668313 | 0.330751488 |
| MEDP1106 | Lithocholic acid | 0.974593034 | 0.317281806 | -1.6561633 |
| MEDP1117 | 7,12-diketocholic acid | 1.149174644 | 1.065098604 | 0.090986998 |
| MEDP1121 | 3-Methacrylic acid | 0.293326241 | 0.945657755 | -0.080609945 |
| MEDP1131 | Arachidyl glycine | 0.35336722 | 0.912253715 | -0.132492973 |
| MEDP1146 | Hemolytic PAF C-18 | 1.306041464 | 0.757274644 | -0.401111472 |
| MEDP1148 | Leukotriene E4 | 0.218278579 | 0.840413099 | -0.250829446 |
| MEDP1160 | PE (18: 1 (9Z) / 0: 0) | 0.958959558 | 1.109316748 | 0.149671364 |
| MEDP1161 | Creatine phosphate | 1.439118442 | 1.209171661 | 0.274019073 |
| MEDP1163 | Palmitoyl-EA | 1.857663666 | 1.561975254 | 0.643371597 |
| MEDP1165 | 2-O-ethyl PAF C-16 | 1.306041464 | 0.757274644 | -0.401111472 |
| MEDP1166 | 1-O-palmitoyl-2-O-acetyl-sn-glycerol-3-phosphate choline | 1.665739322 | 1.483104745 | 0.568620493 |
| MEDP1179 | (±) -Myristylcarnitine | 1.960367549 | 1.63667297 | 0.71076608 |
| MEDP1221 | 2-O-methyl PAF C-16 | 0.574701658 | 1.078412206 | 0.108908731 |

| | | | | |
|---|---|---|---|---|
| MEDP1274 | (4Z, 7Z, 10Z, 13Z, 16Z, 19Z) -Eicosahexaenoate | 0.993048122 | 0.252700309 | -1.984500666 |
| MEDP1289 | Hexanoylcarnitine | 0.803408722 | 1.129347186 | 0.175489071 |
| MEDP1316 | MAG(16:1)isomer | 1.289836216 | 0.81632211 | -0.292789561 |
| MEDP1317 | MAG(16:1) | 1.289836216 | 0.81632211 | -0.292789561 |
| MEDP1318 | LysoPC 22:4 (2n isomer1) | 0.279838849 | 1.033571698 | 0.04763847 |
| MEDP1319 | LysoPC 22:4 | 0.279838849 | 1.033571698 | 0.04763847 |
| MEDP1320 | LysoPC 22:5 (2n isomer3) | 0.318957778 | 1.035054107 | 0.049706186 |
| MEDP1321 | LysoPC 22:5 (2n isomer2) | 0.318957778 | 1.035054107 | 0.049706186 |
| MEDP1322 | LysoPC 22:5 (2n isomer1) | 0.450439758 | 1.066242992 | 0.09253626 |
| MEDP1325 | LysoPC 22:6 | 0.43057323 | 1.02486022 | 0.035427156 |
| MEDP1326 | LysoPC 20:1(2n isomer) | 1.375575143 | 1.241917207 | 0.312568999 |
| MEDP1328 | LysoPC 20:2(2n isomer2) | 1.6155532 | 1.357308911 | 0.440749103 |
| MEDP1330 | LysoPC 20:2(2n isomer1) | 1.6155532 | 1.357308911 | 0.440749103 |
| MEDP1331 | LysoPC 20:3(2n isomer) | 1.361029885 | 1.13503635 | 0.182738501 |
| MEDP1332 | LysoPC 20:3 | 1.361029885 | 1.13503635 | 0.182738501 |
| MEDP1333 | LysoPC 20:4(2n isomer) | 1.46298938 | 0.855598054 | -0.224994894 |
| MEDP1334 | LysoPC 20:4 | 1.46298938 | 0.855598054 | -0.224994894 |
| MEDP1335 | LysoPC 20:5 | 0.758327849 | 1.146813501 | 0.197630794 |
| MEDP1336 | LysoPC 20:5(2n isomer) | 0.694484968 | 1.132718405 | 0.17978925 |
| MEDP1337 | LysoPC 18:0(2n isomer) | 0.038533166 | 1.001636235 | 0.002358659 |
| MEDP1339 | LysoPC 18:1(2n isomer) | 0.522494023 | 1.070350488 | 0.098083287 |
| MEDP1341 | LysoPC 18:2(2n isomer1) | 0.629663178 | 1.082891691 | 0.114888954 |

| | | | | |
|---|---|---|---|---|
| MEDP1345 | LysoPC 18:3(2n isomer2) | 0.101393571 | 1.013154422 | 0.018854083 |
| MEDP1346 | LysoPC 16:0(2n isomer) | 0.079089913 | 1.007069914 | 0.010163843 |
| MEDP1348 | LysoPC 16:1(2n isomer) | 1.810050141 | 1.609006476 | 0.686170132 |
| MEDP1369 | Carnitine C15:0 | 0.598127569 | 1.064312458 | 0.089921755 |
| MEDP1373 | Carnitine C22:2 | 1.015855194 | 1.138827281 | 0.187548959 |
| MEDP1377 | Carnitine C20:1-OH | 1.559216017 | 1.788632673 | 0.838857135 |
| MEDP1380 | Carnitine C20:1 | 1.425884151 | 1.43855451 | 0.524619889 |
| MEDP1381 | Carnitine C20:2 | 1.683374294 | 1.496053884 | 0.581162138 |
| MEDP1382 | Carnitine ph-C14 | 0.292748348 | 1.064368349 | 0.089997514 |
| MEDP1384 | Carnitine C19:0 | 0.150461466 | 1.007517888 | 0.010805453 |
| MEDP1385 | Carnitine C17:1:DC | 1.918963277 | 2.141746896 | 1.098787998 |
| MEDP1386 | Carnitine C18:2-OH | 1.852357825 | 1.629835904 | 0.704726718 |
| MEDP1389 | Carnitine C18:0 | 0.603464544 | 1.092579271 | 0.127737956 |
| MEDP1392 | Carnitine C18:3 | 1.664169171 | 1.397063369 | 0.482397461 |
| MEDP1394 | Carnitine C15:DC | 1.785136704 | 2.048731264 | 1.034730756 |
| MEDP1395 | Carnitine C17:0 | 1.745431635 | 1.318762915 | 0.399185223 |
| MEDP1399 | Carnitine C16:2 | 1.984329574 | 1.754446241 | 0.811015742 |
| MEDP1400 | Carnitine C16:3 | 1.97070823 | 1.551343141 | 0.633517831 |
| MEDP1401 | Carnitine C14-OH | 1.85423044 | 1.771877586 | 0.825278936 |
| MEDP1403 | Carnitine C15:1 | 1.756141481 | 1.669428733 | 0.739354506 |
| MEDP1404 | Carnitine C14:2-OH | 2.090462542 | 1.715461382 | 0.778596649 |
| MEDP1405 | Carnitine C14:0 | 1.895122609 | 1.5669968 | 0.648002233 |
| MEDP1406 | Carnitine C14:1 | 1.778965737 | 1.512543951 | 0.596977065 |
| MEDP1407 | Carnitine C14:2 | 1.787757126 | 1.565963843 | 0.647050902 |
| MEDP1409 | Carnitine C12-OH | 1.932004565 | 1.707580713 | 0.771953773 |
| MEDP1410 | Carnitine C11:DC | 1.901097428 | 1.699941884 | 0.765485426 |
| MEDP1411 | Carnitine C13:0 | 2.008245543 | 1.619053939 | 0.69515105 |

| | | | | |
|---|---|---|---|---|
| MEDP1414 | Carnitine C12:1 | 1.11874605 | 1.23740037 | 0.30731237 |
| MEDP1415 | Carnitine C9:DC | 0.398566638 | 1.031448815 | 0.04467223 |
| MEDP1418 | Carnitine C11:1 | 1.16205544 | 1.467540017 | 0.553399843 |
| MEDP1419 | Carnitine C10:0 | 1.455041578 | 1.268395761 | 0.343004961 |
| MEDP1422 | Carnitine C8-OH | 1.745241891 | 1.481665351 | 0.567219637 |
| MEDP1427 | Carnitine C8:0 | 0.950174395 | 1.192592339 | 0.254100975 |
| MEDP1428 | Carnitine C8:1 | 1.255913342 | 1.217196486 | 0.283562073 |
| MEDP1429 | Carnitine C5:DC | 0.215282626 | 1.019197208 | 0.02743323 |
| MEDP1432 | Carnitine C7:1 | 1.336868922 | 1.361647952 | 0.445353749 |
| MEDP1433 | Carnitine ph-C1 | 0.438402452 | 0.950974564 | -0.072521342 |
| MEDP1434 | Carnitine C4:DC | 1.740769621 | 1.464947377 | 0.550848842 |
| MEDP1435 | Carnitine C6:0 Isomer 2 | 0.803408722 | 1.129347186 | 0.175489071 |
| MEDP1436 | Carnitine C6:0 Isomer 1 | 0.744129334 | 1.108726535 | 0.148903571 |
| MEDP1437 | Carnitine C6:0 | 0.803408722 | 1.129347186 | 0.175489071 |
| MEDP1439 | Carnitine C5:0 Isomer | 0.143330975 | 0.860029248 | -0.217542371 |
| MEDP1440 | Carnitine C5:0 | 0.096071428 | 0.869886017 | -0.201101721 |
| MEDP1441 | Carnitine C5:1 | 0.492586864 | 0.810369158 | -0.303348828 |
| MEDP1442 | Carnitine C4:0 | 0.289750476 | 0.820474075 | -0.285470346 |
| MEDP1444 | Octapentaenoic acid | 1.188412188 | 0.903478695 | -0.146437514 |
| MEDP1457 | Tetracosaenoic acid | 0.98606355 | 1.446370248 | 0.532436907 |
| MEDP1458 | Docosaenoic acid | 1.314554975 | 1.596009775 | 0.674469488 |
| MEDP1460 | Hydroxyeicosapentaenoic acid | 1.10890122 | 1.962239692 | 0.972501281 |
| MEDP1463 | Eicosahexaenoic acid | 0.558643803 | 1.099108529 | 0.136333849 |
| LIPID-N-0001 | 13-oxoODE | 0.74356909 | 0.853961951 | -0.227756304 |
| LIPID-N-0005 | 12,13-EpOME | 0.62692417 | 0.883545751 | -0.178623255 |
| LIPID-N-0006 | 9,10-EpOME | 1.500772356 | 0.559130716 | -0.838742494 |
| LIPID-N-0008 | FFA(20:4) | 0.686026392 | 0.862959614 | -0.212635051 |

| | | | | |
|---|---|---|---|---|
| LIPID-N-0011 | (±)12-HEPE | 1.266773552 | 1.687279329 | 0.754698832 |
| LIPID-N-0013 | (±)18-HEPE | 1.22106813 | 1.668285126 | 0.738365881 |
| LIPID-N-0015 | 15-oxoETE | 0.069875465 | 1.049128325 | 0.069191154 |
| LIPID-N-0017 | 11,12-EET | 0.109354774 | 1.028932553 | 0.041148416 |
| LIPID-N-0021 | (±)12-HETE | 0.59948491 | 1.135791428 | 0.183697928 |
| LIPID-N-0027 | (±)8-HETE | 0.282425664 | 1.362082389 | 0.445813971 |
| LIPID-N-0028 | (±)9-HETE | 0.505555461 | 1.188207819 | 0.248787188 |
| LIPID-N-0036 | 5(S),6(R)-DiHETE | 0.611411571 | 0.782732831 | -0.353408136 |
| LIPID-N-0041 | 16(17)-EpDPE | 1.370165201 | 1.89515832 | 0.922318375 |
| LIPID-N-0045 | 14(S)-HDHA | 1.542614121 | 2.036178181 | 1.025863814 |
| LIPID-N-0048 | PGE3 | 0.923838911 | 0.691722717 | -0.531734257 |
| LIPID-N-0053 | PGE2 | 1.069553021 | 0.597360211 | -0.743326949 |
| LIPID-N-0064 | TxB3 | 0.539349005 | 1.125382216 | 0.17041507 |
| LIPID-N-0065 | TXB2 | 0.51115964 | 1.102885689 | 0.141283267 |
| LIPID-N-0072 | FFA(4:0) | 1.036772031 | 0.839023535 | -0.253216815 |
| LIPID-N-0073 | FFA(5:0) | 0.216248935 | 0.977247761 | -0.033203721 |
| LIPID-N-0074 | FFA(6:0) | 0.483226504 | 0.926814751 | -0.109647088 |
| LIPID-N-0075 | FFA(8:0) | 0.725311645 | 0.926565484 | -0.110035154 |
| LIPID-N-0076 | FFA(10:0) | 1.085634154 | 0.901490196 | -0.149616293 |
| LIPID-N-0077 | FFA(11:0) | 0.282607262 | 1.067342741 | 0.094023523 |
| LIPID-N-0078 | FFA(12:0) | 0.046895143 | 0.992195698 | -0.011303393 |
| LIPID-N-0079 | FFA(13:0) | 1.24604867 | 0.786071347 | -0.347267831 |
| LIPID-N-0080 | FFA(14:0) | 0.469698307 | 0.957251694 | -0.063029787 |
| LIPID-N-0081 | FFA(15:0) | 0.305121537 | 0.959669787 | -0.059390021 |
| LIPID-N-0082 | FFA(16:0) | 0.2753209 | 0.974624869 | -0.037081059 |
| LIPID-N-0083 | FFA(17:0) | 0.294756299 | 1.061488194 | 0.086088325 |
| LIPID-N-0084 | FFA(18:0) | 0.762915072 | 0.913727249 | -0.130164515 |
| LIPID-N-0091 | FFA(16:1) | 0.247257257 | 1.042592205 | 0.060174978 |

| | | | | |
|---|---|---|---|---|
| LIPID-N-0093 | FFA(18:1) | 0.557802831 | 1.084125231 | 0.116531417 |
| LIPID-N-0095 | FFA(20:1) | 0.513614738 | 0.886930755 | -0.17310662 |
| LIPID-N-0098 | FFA(16:2) | 0.136862388 | 1.013538357 | 0.019400688 |
| LIPID-N-0099 | FFA(18:2) | 0.062141266 | 1.014173937 | 0.020305105 |
| LIPID-N-0100 | FFA(20:2) | 1.02552049 | 1.343623467 | 0.426128898 |
| LIPID-N-0103 | FFA(18:3) | 0.458000793 | 0.910360205 | -0.135490601 |
| LIPID-N-0104 | FFA(20:3) | 0.810011147 | 1.273367929 | 0.348649335 |
| LIPID-N-0108 | FFA(22:4) | 0.379484436 | 0.855936404 | -0.224424486 |
| LIPID-N-0109 | FFA(24:4) | 0.439828124 | 1.047879716 | 0.067473123 |
| LIPID-N-0110 | FFA(20:5) | 0.601853715 | 1.138589515 | 0.187247719 |
| LIPID-N-0111 | FFA(22:5) | 0.848455595 | 1.290348319 | 0.367760562 |
| LIPID-N-0112 | FFA(24:5) | 0.905295845 | 1.295502194 | 0.373511458 |
| LIPID-N-0113 | FFA(22:6) | 1.469597959 | 1.581774926 | 0.66154433 |
| LIPID-N-0114 | FFA(24:6) | 0.434374668 | 0.934072994 | -0.0983928 |
| LIPID-N-0115 | LPA(0:0/16:0) | 0.386404516 | 1.030909783 | 0.043918085 |
| LIPID-N-0116 | LPA(0:0/18:0) | 0.450338919 | 1.077060712 | 0.107099575 |
| LIPID-N-0124 | LPC(14:1/0:0) | 1.434209352 | 2.526181269 | 1.336958165 |
| LIPID-N-0126 | LPC(18:1/0:0) | 1.57660289 | 1.466238504 | 0.552119797 |
| LIPID-N-0129 | LPC(18:2/0:0) | 1.174447697 | 1.176969355 | 0.235076758 |
| LIPID-N-0146 | LPE(0:0/18:1) | 1.235299453 | 1.255217827 | 0.327937747 |
| LIPID-N-0149 | LPE(0:0/20:2) | 1.803127635 | 1.684264609 | 0.752118813 |
| LIPID-N-0151 | LPE(0:0/20:3) | 1.524195166 | 1.561626272 | 0.64304923 |
| LIPID-N-0153 | LPE(0:0/22:4) | 0.230125187 | 0.89834151 | -0.154664097 |
| LIPID-N-0155 | LPE(0:0/22:5) | 0.843125843 | 1.449778539 | 0.535832538 |
| LIPID-N-0158 | LPI(14:1/0:0) | 0.97045643 | 1.183531449 | 0.243098042 |
| LIPID-N-0171 | PA(18:0/18:1) | 0.1564301 | 1.000102392 | 0.000147713 |
| LIPID-N-0178 | PC(16:0/14:0) | 0.522991238 | 0.752385921 | -0.410455243 |
| LIPID-N-0188 | PC(16:0/16:1) | 0.677604089 | 1.194704235 | 0.256653504 |

| | | | | |
|---|---|---|---|---|
| LIPID-N-0191 | PC(14:0/18:1) | 0.370868411 | 1.077759628 | 0.108035451 |
| LIPID-N-0193 | PC(16:1/18:0) | 0.864128287 | 1.367825499 | 0.451884189 |
| LIPID-N-0201 | PC(16:0/20:1) | 1.43988011 | 1.912212157 | 0.935242597 |
| LIPID-N-0216 | PC(16:1/18:1) | 2.037339246 | 1.992901197 | 0.994870187 |
| LIPID-N-0224 | PC(18:0/18:2) | 0.880092516 | 0.917876414 | -0.123628178 |
| LIPID-N-0225 | PC(16:0/20:2) | 1.941128157 | 1.886699198 | 0.915864428 |
| LIPID-N-0228 | PC(18:0/20:2) | 0.420454646 | 1.116548187 | 0.159045515 |
| LIPID-N-0229 | PC(18:1/20:1) | 1.624626911 | 1.525516504 | 0.609297788 |
| LIPID-N-0232 | PC(18:2/20:0) | 0.689626748 | 1.276806054 | 0.352539397 |
| LIPID-N-0243 | PC(16:0/18:3) | 1.031318472 | 0.684772081 | -0.546304211 |
| LIPID-N-0244 | PC(16:1/18:2) | 1.729351346 | 1.944145878 | 0.959136475 |
| LIPID-N-0250 | PC(16:0/20:3) | 1.309432419 | 1.464780139 | 0.550684135 |
| LIPID-N-0252 | PC(18:1/18:2) | 1.974239759 | 1.279340573 | 0.355400375 |
| LIPID-N-0255 | PC(18:0/18:3) | 1.087662638 | 0.666649927 | -0.584998726 |
| LIPID-N-0258 | PC(18:0/20:3) | 0.772936802 | 1.164947074 | 0.220264412 |
| LIPID-N-0259 | PC(20:1/18:2) | 0.322862399 | 1.039458285 | 0.055831861 |
| LIPID-N-0271 | PC(16:1/18:3) | 0.209193389 | 2.116552255 | 1.081716107 |
| LIPID-N-0280 | PC(16:0/20:4) | 1.015328522 | 0.86727136 | -0.205444627 |
| LIPID-N-0283 | PC(16:1/20:3) | 0.684705774 | 1.211386554 | 0.276659303 |
| LIPID-N-0285 | PC(18:2/20:2) | 0.872150677 | 1.423717557 | 0.509662967 |
| LIPID-N-0286 | PC(20:3/18:1) | 1.774867153 | 2.042035838 | 1.030008186 |
| LIPID-N-0304 | PC(20:4/16:1) | 0.779021328 | 1.252315326 | 0.324597871 |
| LIPID-N-0309 | PC(18:2/18:3) | 0.272417987 | 1.065593239 | 0.091656833 |
| LIPID-N-0317 | PC(18:2/20:3) | 1.630701018 | 1.839984943 | 0.879693961 |
| LIPID-N-0334 | PC(16:0/22:6) | 0.60076128 | 1.101158945 | 0.139022728 |
| LIPID-N-0341 | PC(18:1/20:5) | 0.488376465 | 1.457231056 | 0.543229647 |
| LIPID-N-0377 | PE(18:1/16:0) | 0.585465675 | 1.267968316 | 0.342518696 |
| LIPID-N-0405 | PE(18:1/16:1) | 0.202492525 | 0.848925626 | -0.23628993 |

| | | | | |
|---|---|---|---|---|
| LIPID-N-0407 | PE(18:2/16:0) | 0.443435152 | 1.179673316 | 0.238387393 |
| LIPID-N-0411 | PE(18:0/18:2) | 0.847969963 | 0.783209701 | -0.35252946 |
| LIPID-N-0448 | PE(20:2/16:1) | 0.539008747 | 0.8434077 | -0.245697903 |
| LIPID-N-0477 | PE(20:4/18:0) | 1.657046862 | 0.662833063 | -0.593282528 |
| LIPID-N-0524 | PE(22:6/16:0) | 0.03994071 | 0.986621211 | -0.019431791 |
| LIPID-N-0531 | PE(22:6/18:0) | 0.504048782 | 0.750281419 | -0.414496265 |
| LIPID-N-0553 | PG(16:0/18:1) | 1.048074294 | 0.528372132 | -0.920373719 |
| LIPID-N-0559 | PG(18:0/18:1) | 0.782446676 | 0.700038758 | -0.514493296 |
| LIPID-N-0562 | PG(18:2/16:0) | 0.633552513 | 0.744961626 | -0.424761982 |
| LIPID-N-0568 | PG(18:2/18:0) | 1.397027635 | 0.419026364 | -1.254887076 |
| LIPID-N-0638 | PI(16:0/20:2) | 0.75206469 | 2.145959228 | 1.101622666 |
| LIPID-N-0640 | PI(18:2/18:0) | 0.922148488 | 1.198986096 | 0.261814929 |
| LIPID-N-0644 | PI(20:3/18:0) | 2.023753077 | 2.210411071 | 1.144314693 |
| LIPID-N-0648 | PI(20:4/16:0) | 0.591076984 | 1.153973763 | 0.206610423 |
| LIPID-N-0653 | PI(18:0/20:4) | 0.504315957 | 1.080890955 | 0.112220985 |
| LIPID-N-0663 | PI(18:1/20:4) | 1.287294845 | 1.240864162 | 0.311345192 |
| LIPID-N-0667 | PI(16:0/22:6) | 0.153124148 | 1.079506333 | 0.110371707 |
| LIPID-N-0686 | PS(18:0/18:2) | 1.391186913 | 1.75131147 | 0.808435689 |
| LIPID-N-0692 | PS(12:0/20:3) | 0.050841137 | 0.978050038 | -0.032019819 |
| LIPID-N-0699 | PS(18:2/18:2) | 0.081334931 | 1.009401225 | 0.013499742 |
| LIPID-N-0701 | PS(16:0/20:4) | 0.878713646 | 0.808355984 | -0.306937328 |
| LIPID-N-0702 | PS(18:0/20:4) | 0.753659242 | 0.846083988 | -0.241127214 |
| LIPID-N-0706 | PS(18:1/20:4) | 0.797955046 | 1.810795525 | 0.856623647 |
| LIPID-N-0711 | PS(20:4/18:2) | 0.118339952 | 1.037633197 | 0.053296541 |
| LIPID-N-0712 | PS(22:6/16:0) | 0.912258142 | 1.547789518 | 0.630209294 |
| LIPID-P-0001 | Free carnitine | 1.49311583 | 1.202154377 | 0.265622175 |
| LIPID-P-0002 | Acetyl-carnitine | 0.891714142 | 1.115327831 | 0.157467828 |
| LIPID-P-0003 | Propionyl-carnitine | 0.480630943 | 1.067303094 | 0.093969933 |

| | | | | |
|---|---|---|---|---|
| LIPID-P-0004 | Butyryl-carnitine | 0.189005826 | 0.845746536 | -0.241702731 |
| LIPID-P-0005 | Isovaleryl-carnitine | 0.323634 | 0.988243984 | -0.017060827 |
| LIPID-P-0006 | hydroxybutyryl-carnitine | 0.371558152 | 1.010279717 | 0.014754788 |
| LIPID-P-0007 | Hexanoyl-carnitine | 0.560265351 | 1.04974271 | 0.070035769 |
| LIPID-P-0008 | hydroxyisovaleroyl-carnitine | 1.454626877 | 1.559267293 | 0.640868259 |
| LIPID-P-0009 | Hydroxyhexanoyl-carnitine | 0.0519472 | 1.004096447 | 0.005897851 |
| LIPID-P-0010 | Octanoyl-carnitine | 1.096680012 | 1.236354287 | 0.306092218 |
| LIPID-P-0011 | 3-Methylglutaryl-carnitine | 1.201164858 | 1.048355181 | 0.068127583 |
| LIPID-P-0013 | Decanoyl-carnitine | 1.532367748 | 1.302226565 | 0.380980475 |
| LIPID-P-0014 | 3-Hydroxy-decanoyl-carnitine | 2.147710564 | 1.961620334 | 0.972045839 |
| LIPID-P-0015 | Lauroyl-carnitine | 2.153481776 | 1.591687507 | 0.670557122 |
| LIPID-P-0016 | 3-Hydroxy-dodecanoyl-carnitine | 1.759283428 | 1.633004425 | 0.7075287 |
| LIPID-P-0017 | Myristoyl-carnitine | 2.03173619 | 1.765577482 | 0.820140135 |
| LIPID-P-0018 | 11-carboxyundecanoyl-carnitine | 0.814110692 | 1.045789334 | 0.064592262 |
| LIPID-P-0019 | 3-Hydroxy-tetradecanoyl- carnitine | 1.702726524 | 1.954681208 | 0.966933335 |
| LIPID-P-0020 | Palmitoyl-carnitine | 1.732890304 | 1.43666251 | 0.522721194 |
| LIPID-P-0022 | Stearoyl-carnitine | 1.510458376 | 1.245312686 | 0.316508035 |
| LIPID-P-0023 | 3-Hydroxy-octadecanoyl-carnitine | 1.123898959 | 1.287901332 | 0.365022071 |
| LIPID-P-0024 | Tiglyl-carnitine | 0.530429702 | 1.205141807 | 0.269202916 |
| LIPID-P-0026 | glutaconyl-carnitine | 0.815905562 | 1.025393035 | 0.036177003 |
| LIPID-P-0029 | Decenoyl-carnitine | 2.027998062 | 1.514612882 | 0.598949104 |

| | | | | |
|---|---|---|---|---|
| LIPID-P-0031 | Dodecenoyl-carnitine | 1.579511491 | 1.611283687 | 0.688210522 |
| LIPID-P-0032 | 3-Hydroxy-dodecenoyl-carnitine | 1.730407389 | 1.410147359 | 0.495845931 |
| LIPID-P-0033 | Tetradecenoyl-carnitine | 1.953975635 | 1.856345356 | 0.892465135 |
| LIPID-P-0034 | 3-Hydroxy-tetradecenoyl- carnitine | 1.86200132 | 2.294049881 | 1.197896761 |
| LIPID-P-0035 | Palmitoleoyl- carnitine | 1.972711668 | 2.092159376 | 1.064992757 |
| LIPID-P-0036 | 3-Hydroxy-palmitoleoyl- carnitine | 1.941695342 | 2.062625125 | 1.04448164 |
| LIPID-P-0037 | Oleyl-carnitine | 1.994233194 | 1.823950389 | 0.867066489 |
| LIPID-P-0038 | 3-Hydroxy-octadecenoyl-carnitine | 2.072449537 | 2.593959173 | 1.375155773 |
| LIPID-P-0039 | Decadienoyl-carnitine | 0.349310489 | 0.984814825 | -0.022075615 |
| LIPID-P-0041 | Palmitodileoyl-carnitine | 2.103161074 | 2.203766917 | 1.139971644 |
| LIPID-P-0042 | Linoleyl-carnitine | 1.68290005 | 1.495218878 | 0.580356689 |
| LIPID-P-0043 | 3-Hydroxy-linoleyl-carnitine | 1.875420965 | 2.012942074 | 1.009305656 |
| LIPID-P-0044 | CE(14:0) | 0.085921923 | 0.997938715 | -0.002976874 |
| LIPID-P-0045 | CE(16:0) | 0.882991596 | 0.900033983 | -0.15194862 |
| LIPID-P-0046 | CE(18:0) | 1.740355696 | 0.607545258 | -0.718936211 |
| LIPID-P-0049 | CE(16:1) | 1.38524535 | 1.357520615 | 0.440974107 |
| LIPID-P-0050 | CE(18:1) | 0.648153712 | 0.932350039 | -0.101056396 |
| LIPID-P-0053 | CE(18:2) | 1.047505133 | 1.201445223 | 0.264770873 |
| LIPID-P-0054 | CE(20:2) | 1.189207577 | 1.164306603 | 0.219471021 |
| LIPID-P-0055 | CE(18:3) | 0.12475337 | 1.017567668 | 0.025124736 |
| LIPID-P-0056 | CE(20:3) | 1.323286017 | 1.413845109 | 0.499624078 |
| LIPID-P-0058 | CE(20:4) | 0.059670004 | 0.933194281 | -0.099750628 |
| LIPID-P-0059 | CE(22:4) | 0.373721375 | 1.063793358 | 0.089217935 |

| | | | | |
|---|---|---|---|---|
| LIPID-P-0060 | CE(20:5) | 1.510367976 | 1.888226062 | 0.917031497 |
| LIPID-P-0062 | CE(22:6) | 0.540525267 | 1.095513673 | 0.131607491 |
| LIPID-P-0068 | Cer(d18:1/16:0) | 1.324165811 | 0.73544074 | -0.443318998 |
| LIPID-P-0069 | Cer(d18:1/18:0) | 1.00937822 | 0.773159492 | -0.371162043 |
| LIPID-P-0070 | Cer(d18:1/20:0) | 0.163958847 | 1.027356018 | 0.038936218 |
| LIPID-P-0071 | Cer(d18:1/22:0) | 0.794446184 | 1.2315479 | 0.300472741 |
| LIPID-P-0072 | Cer(d18:1/24:0) | 1.303695493 | 1.369873516 | 0.454042692 |
| LIPID-P-0073 | Cer(d18:1/16:1) | 1.112063624 | 0.739980911 | -0.434440041 |
| LIPID-P-0077 | Cer(d18:1/24:1) | 1.100653462 | 1.291360058 | 0.36889131 |
| LIPID-P-0089 | Cer(m18:1/20:0) | 0.417779054 | 0.711956031 | -0.490139949 |
| LIPID-P-0094 | Cer(m18:1/22:1) | 0.44035081 | 1.088690928 | 0.122594441 |
| LIPID-P-0096 | CerP(d18:1/12:0) | 0.116008611 | 1.008794707 | 0.012632612 |
| LIPID-P-0097 | CerP(d18:1/14:0) | 1.323185949 | 0.827772069 | -0.272694525 |
| LIPID-P-0098 | CerP(d18:1/16:0) | 0.593498237 | 1.093894524 | 0.129473637 |
| LIPID-P-0099 | CerP(d18:1/18:0) | 1.336573555 | 1.39184321 | 0.476996702 |
| LIPID-P-0104 | CerP(d18:1/16:1) | 2.050630151 | 2.705207544 | 1.435739282 |
| LIPID-P-0105 | CerP(d18:1/18:1) | 1.245246242 | 1.435553732 | 0.521607331 |
| LIPID-P-0111 | Cer(t18:0/22:0) | 0.465242966 | 0.935512467 | -0.096171216 |
| LIPID-P-0112 | Cer(t18:0/24:0) | 0.007749588 | 1.059436623 | 0.083297287 |
| LIPID-P-0114 | Cer(t18:0/20:2) | 0.962166531 | 0.879041119 | -0.185997443 |
| LIPID-P-0117 | DG(14:0/16:0/0:0) | 0.001856449 | 1.020004707 | 0.02857581 |
| LIPID-P-0119 | DG(12:0/20:0/0:0) | 0.323930323 | 0.963535478 | -0.053590306 |
| LIPID-P-0120 | DG(16:0/16:0/0:0) | 0.640928992 | 1.041078587 | 0.058078976 |
| LIPID-P-0121 | DG(14:0/18:0/0:0) | 1.08600532 | 1.114129459 | 0.15591688 |
| LIPID-P-0125 | DG(16:0/18:0/0:0) | 0.065961004 | 1.00538018 | 0.007741153 |
| LIPID-P-0128 | DG(14:0/22:0/0:0) | 0.433960331 | 1.072972406 | 0.101612975 |
| LIPID-P-0129 | DG(18:0/18:0/0:0) | 0.05564608 | 1.017677912 | 0.02528103 |
| LIPID-P-0130 | DG(16:0/20:0/0:0) | 0.390623174 | 1.049177045 | 0.069258148 |

| | | | | |
|---|---|---|---|---|
| LIPID-P-0131 | DG(14:0/16:1/0:0) | 0.790643885 | 1.183078028 | 0.242545227 |
| LIPID-P-0132 | DG(14:1/16:0/0:0) | 0.015261344 | 1.000840347 | 0.001211855 |
| LIPID-P-0133 | DG(14:0/18:1/0:0) | 0.977900458 | 0.392610438 | -1.348829565 |
| LIPID-P-0141 | DG(16:0/18:1/0:0) | 0.561652251 | 0.566172556 | -0.820686275 |
| LIPID-P-0144 | DG(16:0/20:1/0:0) | 1.40223661 | 0.384417413 | -1.379254406 |
| LIPID-P-0145 | DG(16:1/20:0/0:0) | 0.957900442 | 1.061921209 | 0.086676727 |
| LIPID-P-0146 | DG(18:0/18:1/0:0) | 0.8965337 | 0.52675638 | -0.924792212 |
| LIPID-P-0148 | DG(16:1/22:0/0:0) | 0.062355919 | 0.98040482 | -0.028550517 |
| LIPID-P-0152 | DG(14:0/18:2/0:0) | 0.92533866 | 0.519543592 | -0.944683292 |
| LIPID-P-0154 | DG(16:1/18:1/0:0) | 0.129532678 | 0.742517462 | -0.42950314 |
| LIPID-P-0156 | DG(16:0/18:2/0:0) | 0.747458258 | 0.648695698 | -0.624386223 |
| LIPID-P-0157 | DG(18:1/18:1/0:0) | 0.096038156 | 0.853905059 | -0.227852421 |
| LIPID-P-0159 | DG(18:0/18:2/0:0) | 1.077167801 | 0.574591819 | -0.799390642 |
| LIPID-P-0161 | DG(18:1/20:1/0:0) | 0.001333046 | 0.857607498 | -0.221610576 |
| LIPID-P-0162 | DG(18:1/20:0/0:0) | 0.121311351 | 0.930141432 | -0.104477994 |
| LIPID-P-0163 | DG(20:0/18:2/0:0) | 1.78984256 | 0.292942428 | -1.771310937 |
| LIPID-P-0166 | DG(16:1/18:2/0:0) | 0.465865002 | 1.051075274 | 0.071865993 |
| LIPID-P-0176 | DG(18:1/18:2/0:0) | 0.116717452 | 0.988990757 | -0.015971058 |
| LIPID-P-0183 | DG(18:2/20:1/0:0) | 0.805696574 | 0.613191093 | -0.705591355 |
| LIPID-P-0185 | DG(16:1/18:3/0:0) | 0.142272267 | 1.003965395 | 0.005709543 |
| LIPID-P-0187 | DG(14:0/20:4/0:0) | 0.138202924 | 0.98079691 | -0.027973661 |
| LIPID-P-0189 | DG(18:2/18:2/0:0) | 0.306821196 | 0.862022647 | -0.214202323 |
| LIPID-P-0192 | DG(16:0/20:4/0:0) | 1.270107166 | 0.453542498 | -1.140690353 |
| LIPID-P-0203 | DG(16:1/20:4/0:0) | 1.192830323 | 0.439905439 | -1.184734656 |
| LIPID-P-0207 | DG(18:1/20:4/0:0) | 1.11155166 | 0.563219489 | -0.828230839 |
| LIPID-P-0209 | DG(18:1/22:4/0:0) | 0.531551647 | 0.66153226 | -0.596116584 |
| LIPID-P-0213 | DG(18:2/20:4/0:0) | 0.858608661 | 0.691053344 | -0.533131015 |
| LIPID-P-0214 | DG(18:1/20:5/0:0) | 0.618490833 | 1.187192924 | 0.247554398 |

| | | | | |
|---|---|---|---|---|
| LIPID-P-0215 | DG(16:0/22:6/0:0) | 0.381080905 | 0.77388904 | -0.369801367 |
| LIPID-P-0217 | DG(18:2/22:4/0:0) | 0.396125173 | 1.097217603 | 0.133849674 |
| LIPID-P-0223 | LPC(14:0/0:0) | 1.056978942 | 1.170111088 | 0.226645503 |
| LIPID-P-0224 | LPC(16:0/0:0) | 0.902272658 | 1.14350042 | 0.193456896 |
| LIPID-P-0225 | LPC(18:0/0:0) | 0.00030399 | 0.986522581 | -0.019576021 |
| LIPID-P-0226 | LPC(20:0/0:0) | 1.16489234 | 1.394445474 | 0.479691522 |
| LIPID-P-0227 | LPC(22:0/0:0) | 0.886734745 | 1.159687821 | 0.213736496 |
| LIPID-P-0228 | LPC(24:0/0:0) | 0.775184836 | 1.172686116 | 0.22981691 |
| LIPID-P-0230 | LPC(16:1/0:0) | 1.678782883 | 1.599597713 | 0.677709123 |
| LIPID-P-0232 | LPC(20:1/0:0) | 1.419536474 | 1.372784813 | 0.457105497 |
| LIPID-P-0233 | LPC(22:1/0:0) | 1.569972804 | 1.525435526 | 0.609221204 |
| LIPID-P-0235 | LPC(20:2/0:0) | 1.791637086 | 1.536346192 | 0.619503342 |
| LIPID-P-0236 | LPC(22:2/0:0) | 1.727319545 | 1.630461966 | 0.705280787 |
| LIPID-P-0237 | LPC(18:3/0:0) | 0.879206726 | 1.190907711 | 0.252061616 |
| LIPID-P-0238 | LPC(20:3/0:0) | 1.668097963 | 1.556163027 | 0.637993208 |
| LIPID-P-0240 | LPC(20:4/0:0) | 0.286347357 | 1.019165025 | 0.027387674 |
| LIPID-P-0241 | LPC(22:4/0:0) | 0.361827611 | 1.064025602 | 0.089532865 |
| LIPID-P-0242 | LPC(20:5/0:0) | 1.304739647 | 1.400808408 | 0.486259648 |
| LIPID-P-0243 | LPC(22:6/0:0) | 1.104791807 | 1.18210225 | 0.241354832 |
| LIPID-P-0245 | LPC(O-16:0/0:0) | 1.35528227 | 1.567957462 | 0.64888642 |
| LIPID-P-0246 | LPC(O-18:0/0:0) | 0.513186552 | 1.092242697 | 0.127293459 |
| LIPID-P-0247 | LPC(O-20:0/0:0) | 0.67950419 | 0.910631161 | -0.135061266 |
| LIPID-P-0248 | LPC(O-22:0/0:0) | 0.752518689 | 1.109181951 | 0.149496045 |
| LIPID-P-0251 | LPC(O-18:1/0:0) | 1.565608053 | 1.619556595 | 0.695598883 |
| LIPID-P-0252 | LPC(O-20:1/0:0) | 1.774399131 | 1.628998065 | 0.70398489 |
| LIPID-P-0253 | LPC(O-22:1/0:0) | 1.607272492 | 1.673960474 | 0.743265463 |
| LIPID-P-0254 | LPC(O-18:2/0:0) | 0.393070804 | 1.067112283 | 0.093711986 |
| LIPID-P-0259 | LPC(O-20:4/0:0) | 0.774396141 | 1.165090883 | 0.220442496 |

| | | | | |
|---|---|---|---|---|
| LIPID-P-0263 | LPE(0:0/16:0) | 1.155646314 | 1.214041532 | 0.279817776 |
| LIPID-P-0264 | LPE(0:0/18:0) | 0.096152872 | 1.016020282 | 0.022929202 |
| LIPID-P-0265 | LPE(0:0/20:0) | 1.236632301 | 1.409944089 | 0.495637954 |
| LIPID-P-0266 | LPE(0:0/22:0) | 1.393327631 | 1.420894043 | 0.506798976 |
| LIPID-P-0269 | LPE(0:0/16:1) | 1.626519372 | 1.763886839 | 0.818758008 |
| LIPID-P-0271 | LPE(0:0/20:1) | 1.588487059 | 1.529311698 | 0.612882481 |
| LIPID-P-0272 | LPE(0:0/22:1) | 1.918076029 | 2.13935995 | 1.097179238 |
| LIPID-P-0273 | LPE(0:0/24:1) | 1.65478346 | 1.564900393 | 0.646070831 |
| LIPID-P-0274 | LPE(0:0/18:2) | 0.624779888 | 1.120052177 | 0.163565941 |
| LIPID-P-0276 | LPE(0:0/18:3) | 0.528990277 | 0.933687725 | -0.098987978 |
| LIPID-P-0279 | LPE(0:0/20:4) | 0.614979325 | 1.122111327 | 0.166215816 |
| LIPID-P-0281 | LPE(0:0/20:5) | 1.717758282 | 1.455772666 | 0.541785081 |
| LIPID-P-0283 | LPE(0:0/22:6) | 0.79672966 | 1.171423519 | 0.228262765 |
| LIPID-P-0284 | LPE(0:0/24:6) | 0.801889048 | 1.199298819 | 0.262191167 |
| LIPID-P-0287 | LPG(16:0/0:0) | 1.373124831 | 0.683415781 | -0.549164534 |
| LIPID-P-0288 | LPS(16:0/0:0) | 0.779156103 | 1.133318488 | 0.180553348 |
| LIPID-P-0292 | MG(16:0/0:0/0:0) | 1.150764385 | 1.031237864 | 0.044377141 |
| LIPID-P-0293 | MG(18:0/0:0/0:0) | 0.709381919 | 0.984264848 | -0.022881524 |
| LIPID-P-0305 | PC(16:0/16:0) | 0.94491162 | 0.893776922 | -0.162013301 |
| LIPID-P-0308 | PC(16:0/18:0) | 0.554451766 | 0.898067808 | -0.155103715 |
| LIPID-P-0311 | PC(18:0/18:0) | 0.757512831 | 0.80810787 | -0.307380212 |
| LIPID-P-0312 | PC(16:0/22:0) | 0.584721087 | 1.172828868 | 0.229992519 |
| LIPID-P-0323 | PC(16:0/18:1) | 1.560959693 | 1.634061316 | 0.70846212 |
| LIPID-P-0329 | PC(18:0/18:1) | 0.849410673 | 1.186928739 | 0.247233321 |
| LIPID-P-0340 | PC(14:0/18:2) | 0.725587618 | 1.279836529 | 0.35595955 |
| LIPID-P-0346 | PC(12:0/22:2) | 0.277046641 | 1.045098435 | 0.063638832 |
| LIPID-P-0348 | PC(16:0/18:2) | 0.339275226 | 1.0459608 | 0.064828784 |
| LIPID-P-0349 | PC(18:1/18:1) | 1.868673246 | 1.831401706 | 0.872948271 |

| | | | | |
|---|---|---|---|---|
| LIPID-P-0362 | PC(18:2/22:0) | 0.409599389 | 1.183965034 | 0.243626474 |
| LIPID-P-0363 | PC(20:1/22:1) | 0.665448962 | 1.359534283 | 0.443112532 |
| LIPID-P-0371 | PC(14:0/20:3) | 1.686223106 | 1.687795771 | 0.755140344 |
| LIPID-P-0387 | PC(16:1/22:2) | 0.46244043 | 1.085248191 | 0.118025018 |
| LIPID-P-0398 | PC(14:0/20:4) | 0.264743716 | 0.968456707 | -0.046240538 |
| LIPID-P-0402 | PC(18:2/18:2) | 0.177486342 | 1.009246344 | 0.01327836 |
| LIPID-P-0407 | PC(18:3/18:1) | 0.601827392 | 0.941339241 | -0.087213358 |
| LIPID-P-0417 | PC(22:4/16:0) | 1.598737232 | 0.740806791 | -0.432830771 |
| LIPID-P-0418 | PC(18:0/20:4) | 1.577148303 | 0.676908936 | -0.562966334 |
| LIPID-P-0422 | PC(18:0/22:4) | 0.708611562 | 0.814471026 | -0.296064717 |
| LIPID-P-0436 | PC(22:4/14:1) | 0.650031768 | 1.226018736 | 0.293981027 |
| LIPID-P-0440 | PC(16:0/20:5) | 0.949743714 | 1.313820694 | 0.393768395 |
| LIPID-P-0445 | PC(16:0/22:5) | 0.86617847 | 1.103525597 | 0.142120094 |
| LIPID-P-0447 | PC(18:1/20:4) | 0.681738558 | 1.148071781 | 0.199212846 |
| LIPID-P-0449 | PC(20:5/18:0) | 0.564826803 | 0.860714245 | -0.216393749 |
| LIPID-P-0454 | PC(18:0/22:5) | 0.576780491 | 1.104657566 | 0.143599216 |
| LIPID-P-0465 | PC(14:0/22:6) | 0.743959954 | 1.142994476 | 0.192818431 |
| LIPID-P-0469 | PC(18:2/20:4) | 0.15638589 | 0.979174095 | -0.030362705 |
| LIPID-P-0479 | PC(18:0/22:6) | 0.298610848 | 0.883935382 | -0.177987187 |
| LIPID-P-0480 | PC(20:1/20:5) | 0.217013165 | 0.886029628 | -0.174573153 |
| LIPID-P-0489 | PC(16:1/22:6) | 1.457744181 | 1.627819758 | 0.702940965 |
| LIPID-P-0496 | PC(18:1/22:6) | 1.126403448 | 1.24947995 | 0.321327751 |
| LIPID-P-0500 | PC(20:1/22:6) | 1.505469311 | 1.494959457 | 0.58010636 |
| LIPID-P-0504 | PC(18:2/22:6) | 0.768031366 | 1.158099822 | 0.211759612 |
| LIPID-P-0507 | PC(20:2/22:6) | 0.807138774 | 1.226560511 | 0.29461841 |
| LIPID-P-0513 | PC(O-16:0/14:0) | 0.204331019 | 0.939074507 | -0.090688467 |
| LIPID-P-0514 | PC(O-16:0/16:0) | 1.439436488 | 0.770014664 | -0.377042174 |
| LIPID-P-0515 | PC(O-16:0/18:0) | 1.468479591 | 0.775785756 | -0.366269807 |

| | | | | |
|---|---|---|---|---|
| LIPID-P-0516 | PC(O-18:0/18:0) | 0.335508292 | 0.962298538 | -0.055443557 |
| LIPID-P-0520 | PC(O-16:0/14:1) | 1.662097313 | 0.839354684 | -0.25264752 |
| LIPID-P-0522 | PC(O-16:0/18:1) | 0.768784845 | 0.903591975 | -0.146256638 |
| LIPID-P-0524 | PC(O-18:1/20:0) | 0.477662535 | 1.043686041 | 0.061687789 |
| LIPID-P-0530 | PC(O-16:0/18:2) | 0.132053093 | 1.018339133 | 0.026218096 |
| LIPID-P-0531 | PC(O-18:0/18:2) | 1.024411502 | 0.824507001 | -0.278396351 |
| LIPID-P-0532 | PC(O-18:1/20:1) | 0.655383416 | 0.932716909 | -0.100488823 |
| LIPID-P-0535 | PC(O-16:2/18:1) | 0.459110842 | 1.183884805 | 0.24352871 |
| LIPID-P-0540 | PC(O-16:2/18:2) | 0.263377309 | 0.996677198 | -0.004801772 |
| LIPID-P-0541 | PC(O-18:2/18:2) | 1.273905062 | 0.773686893 | -0.370178261 |
| LIPID-P-0542 | PC(O-18:2/20:2) | 1.393262484 | 0.716498456 | -0.480964499 |
| LIPID-P-0547 | PC(O-18:3/18:2) | 0.767487139 | 0.851474565 | -0.23196466 |
| LIPID-P-0548 | PC(O-18:3/20:2) | 1.377895385 | 0.776385737 | -0.36515448 |
| LIPID-P-0551 | PC(O-20:2/24:3) | 0.660169754 | 0.851606351 | -0.231741386 |
| LIPID-P-0552 | PC(O-18:3/18:3) | 0.010913649 | 1.148716815 | 0.200023184 |
| LIPID-P-0553 | PC(O-18:3/20:3) | 1.155497093 | 0.831754376 | -0.265770544 |
| LIPID-P-0558 | PC(O-18:3/20:4) | 0.02541149 | 1.014772516 | 0.021156351 |
| LIPID-P-0559 | PC(O-20:3/20:4) | 0.927999045 | 0.847241644 | -0.239154591 |
| LIPID-P-0560 | PC(O-20:4/22:3) | 0.328742154 | 0.946657838 | -0.079085026 |
| LIPID-P-0562 | PC(O-20:4/20:4) | 0.079693324 | 0.970699445 | -0.042903428 |
| LIPID-P-0565 | PC(O-20:5/22:4) | 0.661691442 | 1.155403623 | 0.208396923 |
| LIPID-P-0568 | PE(16:0/16:0) | 0.171446115 | 0.949021622 | -0.075487137 |
| LIPID-P-0569 | PE(16:0/18:0) | 0.078690138 | 1.021104836 | 0.030130994 |
| LIPID-P-0573 | PE(16:1/14:0) | 1.169765165 | 0.824055634 | -0.279186354 |
| LIPID-P-0580 | PE(18:1/18:0) | 0.142563334 | 0.984486973 | -0.022555979 |
| LIPID-P-0588 | PE(22:2/12:0) | 0.396040462 | 1.004278238 | 0.006159027 |
| LIPID-P-0589 | PE(16:0/18:2) | 0.392581156 | 1.067086306 | 0.093676866 |
| LIPID-P-0594 | PE(18:1/18:1) | 0.775497747 | 0.815475029 | -0.294287395 |

| | | | | |
|---|---|---|---|---|
| LIPID-P-0600 | PE(18:3/16:0) | 1.895950407 | 1.854650061 | 0.891147002 |
| LIPID-P-0603 | PE(18:1/18:2) | 0.853648252 | 1.145505354 | 0.195984201 |
| LIPID-P-0612 | PE(16:0/20:4) | 0.298735501 | 1.055179475 | 0.077488407 |
| LIPID-P-0616 | PE(18:0/20:4) | 0.74824076 | 0.876229166 | -0.190619858 |
| LIPID-P-0617 | PE(18:0/22:4) | 1.216027832 | 0.695351193 | -0.524186287 |
| LIPID-P-0620 | PE(16:1/20:4) | 0.999633151 | 1.453646267 | 0.539676244 |
| LIPID-P-0621 | PE(18:1/20:4) | 0.289670492 | 1.066693509 | 0.093145709 |
| LIPID-P-0622 | PE(20:1/20:4) | 1.532321242 | 0.628744826 | -0.669453472 |
| LIPID-P-0628 | PE(16:0/22:6) | 0.454567282 | 1.105555298 | 0.144771188 |
| LIPID-P-0631 | PE(18:0/22:6) | 0.390853175 | 0.951227131 | -0.072138231 |
| LIPID-P-0635 | PE(22:6/18:1) | 0.492698426 | 1.092308907 | 0.127380911 |
| LIPID-P-0636 | PE(22:6/20:1) | 0.662022096 | 1.269686 | 0.344471755 |
| LIPID-P-0637 | PE(20:3/20:5) | 0.199496731 | 0.95762212 | -0.062471617 |
| LIPID-P-0639 | PE(P-18:0/18:0) | 0.062545007 | 1.030447585 | 0.043271122 |
| LIPID-P-0640 | PE(P-18:0/20:0) | 0.483749725 | 1.138552336 | 0.187200609 |
| LIPID-P-0641 | PE(P-20:0/20:0) | 0.441763027 | 0.866496297 | -0.20673451 |
| LIPID-P-0642 | PE(P-18:1/16:0) | 0.202341382 | 1.043994219 | 0.062113723 |
| LIPID-P-0643 | PE(P-18:1/18:0) | 0.018222827 | 1.056385373 | 0.079136231 |
| LIPID-P-0644 | PE(P-18:1/20:0) | 0.514666286 | 1.143734923 | 0.193752725 |
| LIPID-P-0645 | PE(P-20:1/20:0) | 0.681700865 | 1.181380063 | 0.240473171 |
| LIPID-P-0646 | PE(P-18:2/16:0) | 0.559384749 | 1.217986445 | 0.284498078 |
| LIPID-P-0647 | PE(P-18:2/18:0) | 0.028199252 | 1.030061113 | 0.042729934 |
| LIPID-P-0649 | PE(P-20:2/20:0) | 0.485707679 | 0.898746524 | -0.15401381 |
| LIPID-P-0652 | PE(P-18:2/18:1) | 0.785763249 | 0.867764146 | -0.204625117 |
| LIPID-P-0653 | PE(P-18:2/20:1) | 1.059229986 | 0.78305212 | -0.352819759 |
| LIPID-P-0654 | PE(P-20:2/20:1) | 0.127276587 | 0.862443624 | -0.213497941 |
| LIPID-P-0655 | PE(P-20:2/22:1) | 0.347980128 | 0.791311891 | -0.337681658 |
| LIPID-P-0657 | PE(P-18:2/18:2) | 0.411206958 | 0.936192948 | -0.095122198 |

| | | | | |
|---|---|---|---|---|
| LIPID-P-0658 | PE(P-18:2/20:2) | 1.231436158 | 0.752450255 | -0.410331888 |
| LIPID-P-0659 | PE(P-20:2/20:2) | 0.565066363 | 1.196529801 | 0.25885633 |
| LIPID-P-0662 | PE(P-18:2/20:3) | 0.296283463 | 0.974558837 | -0.037178806 |
| LIPID-P-0663 | PE(P-20:2/20:3) | 0.70916294 | 0.862547846 | -0.213323608 |
| LIPID-P-0664 | PE(P-20:2/22:3) | 0.530708047 | 0.869471459 | -0.201789424 |
| LIPID-P-0665 | PE(P-20:2/24:3) | 0.099082336 | 0.911170031 | -0.134207798 |
| LIPID-P-0666 | PE(P-18:2/20:4) | 0.069512251 | 1.015074505 | 0.021585623 |
| LIPID-P-0667 | PE(P-20:2/20:4) | 0.618088399 | 0.871043182 | -0.199183852 |
| LIPID-P-0668 | PE(P-20:2/22:4) | 0.18142741 | 1.077790111 | 0.108076254 |
| LIPID-P-0669 | PE(P-20:2/24:4) | 0.682100274 | 0.861134475 | -0.215689548 |
| LIPID-P-0670 | PE(P-20:2/20:5) | 0.285242779 | 0.954441997 | -0.067270569 |
| LIPID-P-0673 | PE(P-20:2/20:6) | 0.091946549 | 0.959193631 | -0.060106016 |
| LIPID-P-0712 | PS(18:0/22:6) | 0.451498725 | 0.789551891 | -0.340894009 |
| LIPID-P-0716 | SM(d18:0/12:0) | 0.628664602 | 0.942300677 | -0.085740615 |
| LIPID-P-0722 | SM(d18:0/24:0) | 1.046871186 | 1.261495862 | 0.335135474 |
| LIPID-P-0724 | SM(d18:1/12:0) | 0.147906038 | 0.987178428 | -0.018617226 |
| LIPID-P-0725 | SM(d18:1/14:0) | 0.87437397 | 0.916183542 | -0.126291448 |
| LIPID-P-0726 | SM(d18:1/16:0) | 0.858483279 | 0.932280702 | -0.10116369 |
| LIPID-P-0728 | SM(d18:1/18:0) | 1.15656703 | 0.889885381 | -0.168308569 |
| LIPID-P-0729 | SM(d18:1/20:0) | 0.566831272 | 0.891579521 | -0.165564616 |
| LIPID-P-0730 | SM(d18:1/22:0) | 0.464750353 | 1.102268174 | 0.140475264 |
| LIPID-P-0731 | SM(d18:1/24:0) | 1.357728344 | 1.341999377 | 0.424384002 |
| LIPID-P-0734 | SM(d18:2/14:0) | 0.500746137 | 1.069608923 | 0.097083406 |
| LIPID-P-0735 | SM(d18:1/16:1) | 0.131267765 | 1.023128577 | 0.03298746 |
| LIPID-P-0736 | SM(d18:1/18:1) | 1.024312723 | 0.859401479 | -0.218595834 |
| LIPID-P-0737 | SM(d18:1/20:1) | 1.250096616 | 0.842744878 | -0.246832142 |
| LIPID-P-0738 | SM(d18:1/22:1) | 0.096475875 | 1.000235436 | 0.000339622 |
| LIPID-P-0739 | SM(d18:1/24:1) | 0.730087328 | 1.084022751 | 0.116395035 |

| | | | | |
|---|---|---|---|---|
| LIPID-P-0742 | SM(d18:2/18:1) | 0.469946228 | 1.099192699 | 0.136444327 |
| LIPID-P-0744 | SM(d18:2/22:1) | 1.070181317 | 1.301272868 | 0.379923517 |
| LIPID-P-0745 | SM(d18:2/24:1) | 0.316500794 | 1.026161255 | 0.037257459 |
| LIPID-P-0749 | TG(14:0/16:0/16:0) | 1.476513014 | 0.282505301 | -1.823650156 |
| LIPID-P-0751 | TG(14:0/16:0/18:0) | 1.435814477 | 0.393345654 | -1.346130452 |
| LIPID-P-0754 | TG(16:0/16:0/18:0) | 0.721839848 | 0.844214938 | -0.244317738 |
| LIPID-P-0755 | TG(14:0/18:0/18:0) | 0.656397537 | 0.863644577 | -0.211490384 |
| LIPID-P-0757 | TG(14:0/16:0/22:0) | 0.601082035 | 0.930620703 | -0.103734813 |
| LIPID-P-0760 | TG(14:0/18:0/20:0) | 0.035895705 | 1.005975931 | 0.008595788 |
| LIPID-P-0764 | TG(16:0/16:0/22:0) | 1.366361945 | 0.682909708 | -0.550233252 |
| LIPID-P-0765 | TG(18:0/18:0/18:0) | 0.034431788 | 1.005842265 | 0.008404082 |
| LIPID-P-0783 | TG(14:0/16:0/16:1) | 0.976011573 | 0.271955476 | -1.878557621 |
| LIPID-P-0785 | TG(14:0/14:0/18:1) | 1.48235296 | 0.307931546 | -1.699318426 |
| LIPID-P-0786 | TG(14:0/14:0/20:1) | 1.159934161 | 0.273367272 | -1.871087564 |
| LIPID-P-0787 | TG(14:0/16:0/18:1) | 1.061922928 | 0.293704509 | -1.767562678 |
| LIPID-P-0788 | TG(16:0/16:0/16:1) | 1.055419537 | 0.275659415 | -1.859041219 |
| LIPID-P-0789 | TG(16:0/16:0/18:1) | 1.057369624 | 0.347412573 | -1.52527813 |
| LIPID-P-0790 | TG(14:0/18:0/18:1) | 1.213259784 | 0.34372118 | -1.540689344 |
| LIPID-P-0791 | TG(14:0/16:1/20:0) | 1.48253719 | 0.291564714 | -1.778111961 |
| LIPID-P-0794 | TG(14:0/18:0/20:1) | 1.49239547 | 0.396801714 | -1.333509836 |
| LIPID-P-0795 | TG(16:0/16:1/20:0) | 1.425909869 | 0.314770776 | -1.66762649 |
| LIPID-P-0796 | TG(14:0/18:1/20:0) | 1.285484119 | 0.317724402 | -1.654152198 |
| LIPID-P-0803 | TG(16:0/16:1/22:0) | 1.390799701 | 0.326259335 | -1.615908912 |
| LIPID-P-0805 | TG(18:0/18:0/18:1) | 1.408616333 | 0.370385392 | -1.432900894 |
| LIPID-P-0807 | TG(14:0/20:0/20:1) | 1.267114146 | 0.335781862 | -1.574403794 |
| LIPID-P-0838 | TG(14:0/14:0/18:2) | 0.715039932 | 0.61093893 | -0.710899922 |
| LIPID-P-0839 | TG(14:1/16:0/16:1) | 1.081072688 | 0.487905336 | -1.035326835 |
| LIPID-P-0840 | TG(14:0/16:1/18:1) | 0.453709127 | 0.471406834 | -1.084955421 |

| | | | | |
|---|---|---|---|---|
| LIPID-P-0842 | TG(16:0/16:1/16:1) | 0.806916584 | 0.412765957 | -1.276604104 |
| LIPID-P-0844 | TG(14:0/16:0/18:2) | 1.217723161 | 0.382840197 | -1.385185779 |
| LIPID-P-0845 | TG(14:0/18:0/18:2) | 0.460973632 | 0.61441937 | -0.702704396 |
| LIPID-P-0846 | TG(14:0/18:1/18:1) | 0.480014528 | 0.528803587 | -0.919196132 |
| LIPID-P-0847 | TG(16:0/16:1/18:1) | 0.50513597 | 0.519177049 | -0.945701488 |
| LIPID-P-0852 | TG(16:0/16:1/20:1) | 0.564930913 | 0.578885059 | -0.788651173 |
| LIPID-P-0853 | TG(14:0/18:1/20:1) | 0.346013528 | 0.695788003 | -0.523280292 |
| LIPID-P-0856 | TG(14:0/18:0/20:2) | 1.255163681 | 0.421714036 | -1.245663055 |
| LIPID-P-0858 | TG(16:0/16:1/22:1) | 0.133556941 | 0.795372668 | -0.330297108 |
| LIPID-P-0859 | TG(14:0/20:1/20:1) | 0.310176132 | 0.742114082 | -0.430287111 |
| LIPID-P-0860 | TG(16:1/18:1/20:0) | 0.575864373 | 0.695605868 | -0.523657993 |
| LIPID-P-0861 | TG(18:0/18:1/18:1) | 0.812971768 | 0.68773038 | -0.540085017 |
| LIPID-P-0863 | TG(14:0/18:2/22:0) | 1.206318177 | 0.459400699 | -1.122175044 |
| LIPID-P-0864 | TG(14:0/20:0/20:2) | 1.545060377 | 0.314943025 | -1.666837233 |
| LIPID-P-0865 | TG(14:0/20:1/22:1) | 1.586090859 | 0.372015794 | -1.426564224 |
| LIPID-P-0869 | TG(18:1/18:1/20:0) | 1.031911492 | 0.624520806 | -0.67917846 |
| LIPID-P-0874 | TG(16:0/18:2/22:0) | 1.824743112 | 0.300131806 | -1.736331881 |
| LIPID-P-0897 | TG(12:0/18:0/18:3) | 0.347576847 | 0.791389543 | -0.337540092 |
| LIPID-P-0898 | TG(14:1/16:1/18:1) | 0.101704187 | 0.719348799 | -0.475236619 |
| LIPID-P-0900 | TG(14:0/16:1/18:2) | 0.277176319 | 0.680696155 | -0.554917135 |
| LIPID-P-0902 | TG(14:0/16:0/18:3) | 0.77147495 | 0.489440942 | -1.030793307 |
| LIPID-P-0906 | TG(14:0/18:1/18:2) | 0.395969534 | 0.728631258 | -0.456739207 |
| LIPID-P-0907 | TG(16:0/16:1/18:2) | 0.079471904 | 0.731235728 | -0.451591532 |
| LIPID-P-0910 | TG(14:0/18:0/18:3) | 1.30670375 | 0.336724965 | -1.570357406 |
| LIPID-P-0913 | TG(14:0/18:1/20:2) | 0.04078336 | 0.908931034 | -0.137757263 |
| LIPID-P-0915 | TG(14:0/18:2/20:1) | 0.288568519 | 0.770376305 | -0.376364766 |
| LIPID-P-0917 | TG(16:0/16:1/20:2) | 0.260162163 | 0.770996625 | -0.37520355 |
| LIPID-P-0918 | TG(14:0/18:0/20:3) | 0.568945979 | 0.569359588 | -0.812587997 |

| | | | | |
|---|---|---|---|---|
| LIPID-P-0921 | TG(16:0/16:0/22:3) | 0.351527819 | 0.772625402 | -0.372158984 |
| LIPID-P-0922 | TG(16:1/18:2/20:0) | 0.698847781 | 1.264391506 | 0.338443248 |
| LIPID-P-0923 | TG(14:0/20:1/20:2) | 0.175244503 | 0.768123374 | -0.380590044 |
| LIPID-P-0928 | TG(18:0/18:1/18:2) | 0.955771593 | 0.663347492 | -0.592163275 |
| LIPID-P-0931 | TG(18:1/18:2/20:0) | 1.0324823 | 1.463713771 | 0.549633462 |
| LIPID-P-0932 | TG(14:0/20:1/22:2) | 0.67847783 | 1.20948614 | 0.274394236 |
| LIPID-P-0936 | TG(14:0/20:2/22:1) | 0.449709867 | 0.757702721 | -0.400296166 |
| LIPID-P-0937 | TG(16:0/18:0/22:3) | 0.874895877 | 0.591727325 | -0.756995576 |
| LIPID-P-0940 | TG(14:0/20:0/22:3) | 1.209634193 | 0.57733347 | -0.79252323 |
| LIPID-P-0971 | TG(12:0/18:2/18:2) | 0.344379144 | 0.811840525 | -0.300731737 |
| LIPID-P-0977 | TG(16:1/16:1/18:2) | 0.409172417 | 0.979050405 | -0.030544957 |
| LIPID-P-0978 | TG(14:0/16:0/20:4) | 1.415532548 | 0.264926764 | -1.916334499 |
| LIPID-P-0980 | TG(14:0/18:1/18:3) | 0.321558714 | 0.858806961 | -0.21959421 |
| LIPID-P-0983 | TG(16:0/16:1/18:3) | 0.355207408 | 0.657505231 | -0.604925723 |
| LIPID-P-0985 | TG(14:0/18:1/20:3) | 0.536126094 | 1.116264639 | 0.158679095 |
| LIPID-P-0987 | TG(14:0/18:2/20:2) | 0.011326665 | 0.969192984 | -0.045144133 |
| LIPID-P-0989 | TG(14:0/16:0/22:4) | 0.575339112 | 0.790645754 | -0.338896651 |
| LIPID-P-0991 | TG(14:0/18:0/20:4) | 1.107108339 | 0.305174671 | -1.71229287 |
| LIPID-P-0993 | TG(18:1/18:1/18:2) | 0.561387949 | 1.177295132 | 0.23547603 |
| LIPID-P-0996 | TG(14:0/20:2/20:2) | 0.174410776 | 0.907793517 | -0.139563909 |
| LIPID-P-0997 | TG(16:0/16:1/22:3) | 0.156600216 | 0.808390277 | -0.306876126 |
| LIPID-P-0998 | TG(14:0/18:0/22:4) | 0.926642258 | 0.610897528 | -0.710997693 |
| LIPID-P-0999 | TG(14:0/20:1/20:3) | 0.306646717 | 0.657243283 | -0.605500603 |
| LIPID-P-1002 | TG(14:0/20:0/20:4) | 1.101743859 | 0.420382695 | -1.250224812 |
| LIPID-P-1003 | TG(18:1/18:3/20:0) | 0.155335048 | 1.006498154 | 0.009344526 |
| LIPID-P-1004 | TG(14:0/20:2/22:2) | 1.100534259 | 1.505862015 | 0.590589579 |
| LIPID-P-1005 | TG(16:1/20:1/20:2) | 0.651068067 | 0.763882544 | -0.388577271 |
| LIPID-P-1006 | TG(18:2/18:2/20:0) | 0.402688911 | 0.816483738 | -0.292503943 |

| | | | | |
|---|---|---|---|---|
| LIPID-P-1012 | TG(18:0/18:3/20:1) | 0.036258504 | 0.920820719 | -0.1190078 |
| LIPID-P-1018 | TG(18:1/20:1/20:2) | 0.229917649 | 1.031364238 | 0.044553927 |
| LIPID-P-1019 | TG(14:0/22:1/22:3) | 0.044601034 | 0.976848842 | -0.033792758 |
| LIPID-P-1020 | TG(18:2/18:2/22:0) | 0.750166941 | 0.670514753 | -0.576659019 |
| LIPID-P-1032 | TG(16:0/16:1/18:4) | 0.545903336 | 1.116005357 | 0.158343952 |
| LIPID-P-1036 | TG(14:0/18:2/18:3) | 0.078501547 | 0.880134215 | -0.184204553 |
| LIPID-P-1040 | TG(16:1/16:1/20:3) | 0.720798563 | 1.336541631 | 0.418504776 |
| LIPID-P-1043 | TG(16:0/16:1/20:4) | 0.482445644 | 0.750114227 | -0.41481779 |
| LIPID-P-1044 | TG(14:0/18:3/20:2) | 0.365406716 | 0.773190187 | -0.371104768 |
| LIPID-P-1048 | TG(14:0/18:1/20:4) | 0.988472573 | 0.347766968 | -1.523807186 |
| LIPID-P-1050 | TG(18:1/18:2/18:2) | 0.138767692 | 1.013176686 | 0.018885785 |
| LIPID-P-1052 | TG(18:0/18:2/18:3) | 0.076847703 | 0.974841806 | -0.036759973 |
| LIPID-P-1057 | TG(16:0/16:1/22:4) | 1.16875863 | 0.399594569 | -1.323391119 |
| LIPID-P-1058 | TG(14:0/20:1/20:4) | 1.114049092 | 0.427744996 | -1.225177116 |
| LIPID-P-1059 | TG(18:2/18:3/20:0) | 1.197327777 | 0.489085561 | -1.03184122 |
| LIPID-P-1061 | TG(18:1/18:3/20:1) | 1.256586754 | 0.472464969 | -1.081720732 |
| LIPID-P-1064 | TG(16:0/18:2/22:3) | 1.008062831 | 0.449829812 | -1.152548818 |
| LIPID-P-1065 | TG(14:0/20:1/22:4) | 0.961189988 | 0.409434684 | -1.288294773 |
| LIPID-P-1066 | TG(14:0/20:4/22:1) | 0.978665492 | 0.575285177 | -0.797650798 |
| LIPID-P-1067 | TG(18:0/18:3/20:2) | 0.488990646 | 0.680897717 | -0.554489999 |
| LIPID-P-1070 | TG(18:2/18:3/22:0) | 1.358860089 | 0.544159077 | -0.877899631 |
| LIPID-P-1071 | TG(14:0/22:1/22:4) | 1.282320274 | 0.491151389 | -1.025760315 |
| LIPID-P-1078 | TG(16:0/20:4/22:1) | 1.402974464 | 0.46992346 | -1.089502302 |
| LIPID-P-1083 | TG(14:0/18:2/20:4) | 0.398318507 | 1.166630164 | 0.222347282 |
| LIPID-P-1084 | TG(16:1/18:1/18:4) | 0.361360251 | 1.09464783 | 0.1304668 |
| LIPID-P-1085 | TG(14:0/18:3/20:3) | 0.007616368 | 1.012471096 | 0.017880723 |
| LIPID-P-1090 | TG(16:0/16:1/20:5) | 0.106124998 | 0.822787077 | -0.28140896 |
| LIPID-P-1092 | TG(16:0/16:0/22:6) | 0.574677278 | 0.6251306 | -0.677770472 |

| | | | | |
|---|---|---|---|---|
| LIPID-P-1093 | TG(18:2/18:2/18:2) | 1.13584531 | 0.531518722 | -0.911807586 |
| LIPID-P-1094 | TG(18:1/18:2/18:3) | 0.317190833 | 0.833899379 | -0.262054782 |
| LIPID-P-1095 | TG(14:0/18:3/22:3) | 0.107230845 | 0.926125154 | -0.110720927 |
| LIPID-P-1099 | TG(14:0/20:2/20:4) | 0.69842365 | 0.636996529 | -0.650642583 |
| LIPID-P-1100 | TG(16:0/16:1/22:5) | 0.873435019 | 0.578126178 | -0.790543693 |
| LIPID-P-1103 | TG(14:0/20:1/22:5) | 1.31128399 | 0.438090659 | -1.190698641 |
| LIPID-P-1105 | TG(14:0/20:2/22:4) | 0.786656145 | 0.56512227 | -0.823365053 |
| LIPID-P-1106 | TG(14:0/20:4/22:2) | 0.468600454 | 1.111716499 | 0.15278893 |
| LIPID-P-1107 | TG(18:1/18:3/20:2) | 0.383860338 | 0.745327217 | -0.424054151 |
| LIPID-P-1108 | TG(16:0/18:3/22:3) | 0.531351169 | 0.633134027 | -0.659417162 |
| LIPID-P-1110 | TG(18:0/18:3/20:3) | 1.275149075 | 0.461630162 | -1.115190606 |
| LIPID-P-1115 | TG(16:0/20:4/22:2) | 0.088564942 | 0.996036148 | -0.005729994 |
| LIPID-P-1125 | TG(18:2/18:2/18:3) | 1.062768088 | 0.558668714 | -0.839935064 |
| LIPID-P-1126 | TG(18:1/18:3/18:3) | 0.987587932 | 0.602659559 | -0.730584837 |
| LIPID-P-1129 | TG(14:0/20:3/20:4) | 0.297660529 | 0.855867828 | -0.224540077 |
| LIPID-P-1131 | TG(14:0/20:2/20:5) | 0.511916496 | 1.282392155 | 0.358837505 |
| LIPID-P-1133 | TG(16:0/16:1/22:6) | 0.659335451 | 1.207898819 | 0.272499611 |
| LIPID-P-1136 | TG(14:0/20:4/22:3) | 0.249409497 | 0.956220328 | -0.06458502 |
| LIPID-P-1139 | TG(18:2/18:3/20:2) | 0.035296685 | 1.036019763 | 0.051051524 |
| LIPID-P-1140 | TG(14:0/20:2/22:5) | 0.445801231 | 0.818105883 | -0.289640519 |
| LIPID-P-1141 | TG(16:0/18:1/22:6) | 0.822632368 | 1.358463781 | 0.441976102 |
| LIPID-P-1142 | TG(14:0/22:3/22:4) | 0.451112061 | 1.330759702 | 0.412250084 |
| LIPID-P-1147 | TG(18:0/18:1/22:6) | 0.14798526 | 0.89054009 | -0.167247535 |
| LIPID-P-1155 | TG(14:0/20:3/20:5) | 1.341043764 | 2.135247208 | 1.094403107 |
| LIPID-P-1156 | TG(16:1/16:1/22:6) | 0.790678543 | 1.634610194 | 0.708946637 |
| LIPID-P-1157 | TG(14:0/20:4/20:4) | 1.341066958 | 2.041481815 | 1.029616716 |
| LIPID-P-1158 | TG(14:0/18:2/22:6) | 1.148632516 | 2.061261043 | 1.043527223 |
| LIPID-P-1160 | TG(14:0/20:4/22:4) | 0.239800819 | 0.960047361 | -0.058822516 |

| | | | | |
|---|---|---|---|---|
| LIPID-P-1161 | TG(14:0/20:5/22:3) | 1.042159092 | 1.610186303 | 0.687227622 |
| LIPID-P-1163 | TG(18:1/18:3/20:4) | 1.446064449 | 1.967141563 | 0.976100783 |
| LIPID-P-1164 | TG(18:2/18:3/20:3) | 0.877684586 | 1.898329381 | 0.924730337 |
| LIPID-P-1165 | TG(16:0/18:3/22:5) | 0.935284339 | 1.780152804 | 0.832001084 |
| LIPID-P-1166 | TG(14:0/20:2/22:6) | 1.055529722 | 1.915907198 | 0.938027682 |
| LIPID-P-1170 | TG(14:0/22:3/22:5) | 0.816287767 | 1.543502178 | 0.626207518 |
| LIPID-P-1171 | TG(16:0/20:5/22:3) | 0.634504893 | 1.236884589 | 0.306710891 |
| LIPID-P-1172 | TG(18:1/18:3/22:4) | 1.599273373 | 2.441159939 | 1.28756682 |
| LIPID-P-1173 | TG(14:0/22:2/22:6) | 1.597785318 | 2.30779137 | 1.206512807 |
| LIPID-P-1178 | TG(14:0/20:5/22:4) | 0.434773047 | 1.343157576 | 0.425628568 |
| LIPID-P-1179 | TG(18:2/18:3/20:4) | 1.055087965 | 1.836132848 | 0.876670445 |
| LIPID-P-1180 | TG(16:1/20:4/20:4) | 1.766244038 | 3.282039676 | 1.71459268 |
| LIPID-P-1182 | TG(14:0/20:3/22:6) | 1.45216261 | 2.526690308 | 1.337248846 |
| LIPID-P-1184 | TG(18:2/18:3/22:4) | 1.322455703 | 2.804793542 | 1.487894579 |
| LIPID-P-1185 | TG(14:0/22:3/22:6) | 1.477717897 | 2.806816084 | 1.488934534 |
| LIPID-P-1186 | TG(18:1/18:3/22:5) | 1.368620696 | 2.231708381 | 1.158148522 |
| LIPID-P-1187 | TG(16:0/20:4/22:5) | 1.496658245 | 0.492659564 | -1.021337031 |
| LIPID-P-1189 | TG(18:0/20:3/22:6) | 1.137278341 | 1.63419888 | 0.708583568 |
| LIPID-P-1195 | TG(18:2/18:3/22:5) | 0.857216405 | 1.692589792 | 0.759232371 |
| LIPID-P-1196 | TG(16:0/20:4/22:6) | 0.89322378 | 1.909246701 | 0.933003531 |

Western Blot image

Fig4G-CPT1A

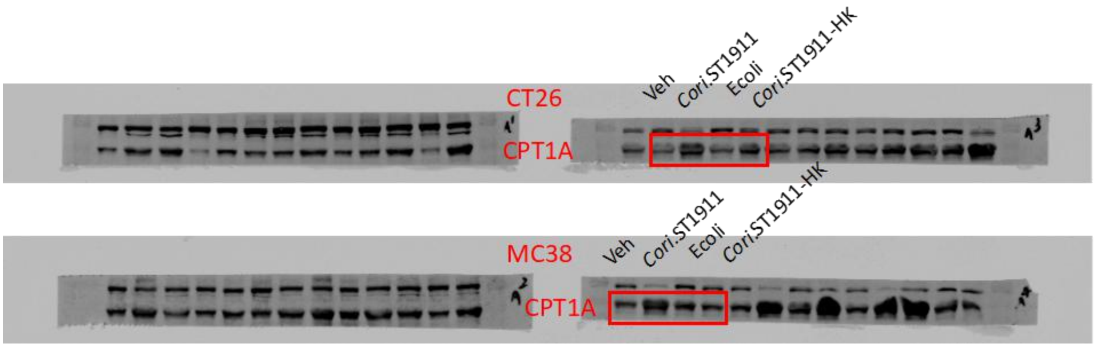


Fig4G-CPT2

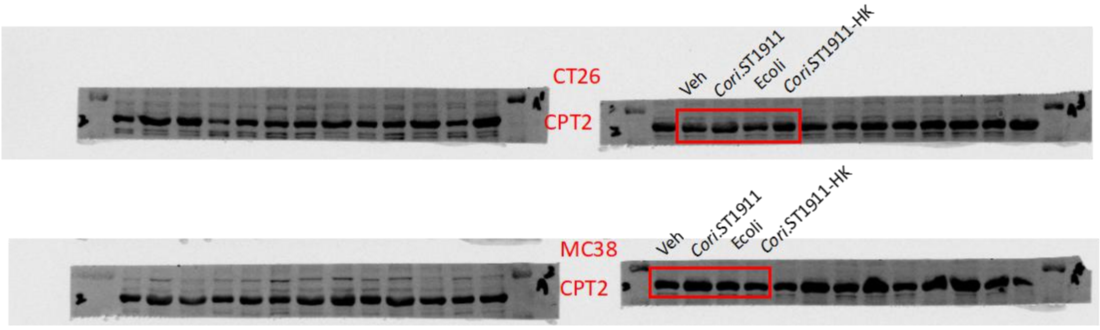


Fig4G-GAPDH

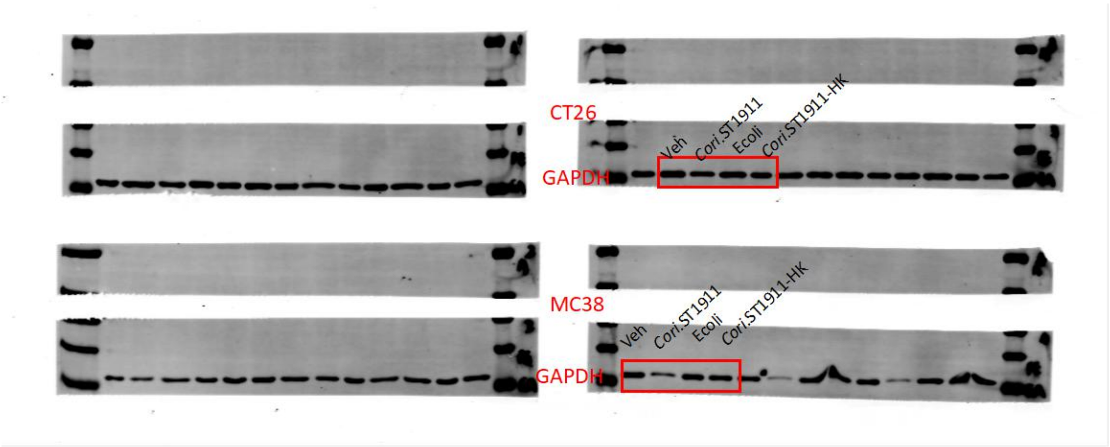

Fig5B

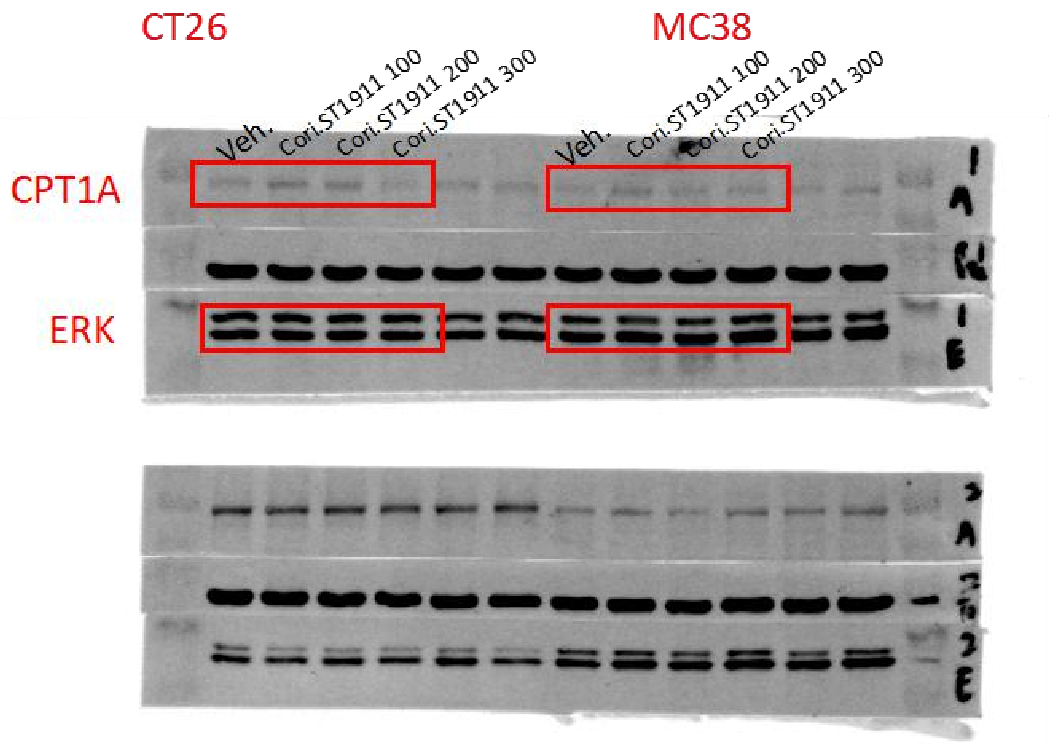
CT26
MC38
Veh.
Cori.ST1911 100
Cori.ST1911 200
Cori.ST1911 300
Veh.
Cori.ST1911 100
Cori.ST1911 200
Cori.ST1911 300
CPT1A
ERK

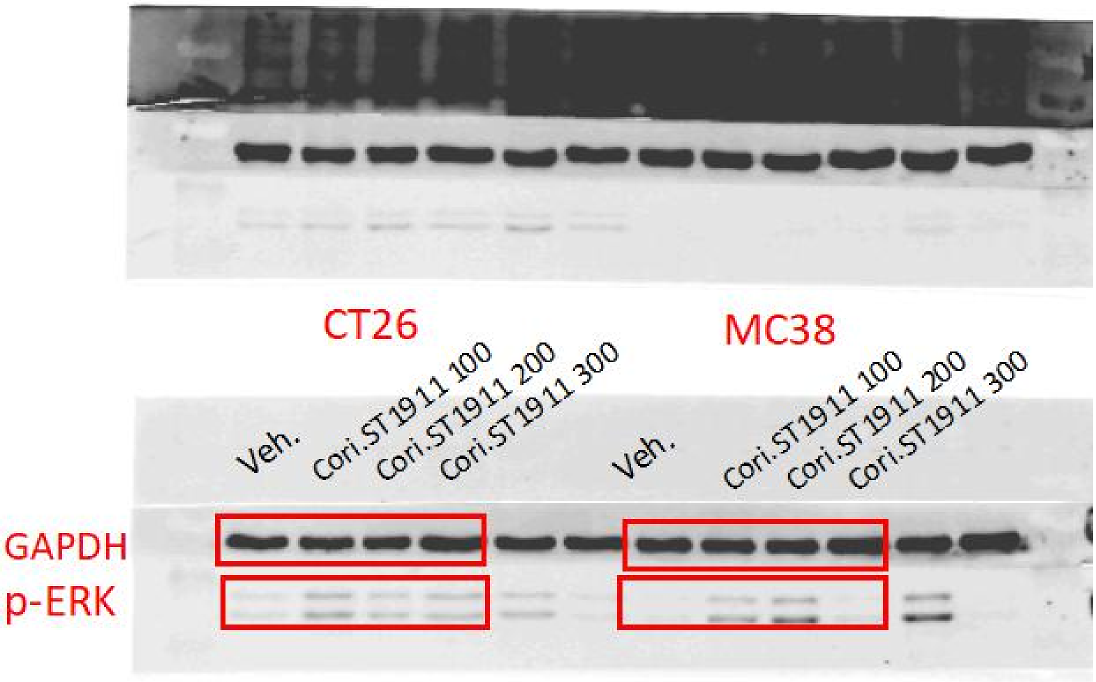
CT26
MC38
Veh.
Cori.ST1911 100
Cori.ST1911 200
Cori.ST19 11 300
Veh.
Cori.ST1911 100
Cori.ST 1911 200
Cori.ST1911 300
GAPDH
p-ERK

Fig5C

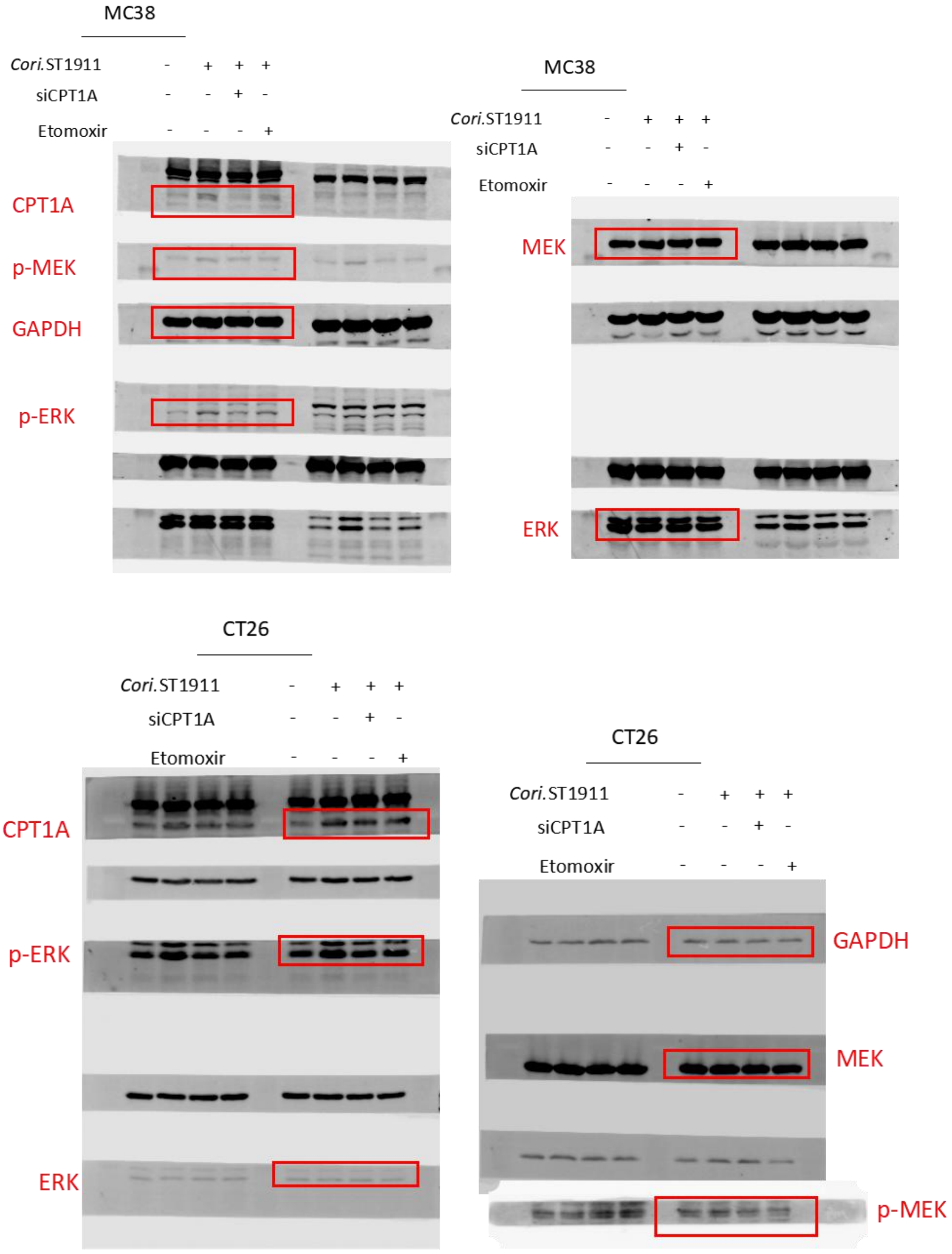
MC38
Cori.ST1911 - + + +
siCPT1A - - + -
Etomoxir - - - +
CPT1A
p-MEK
GAPDH
p-ERK
MC38
Cori.ST1911 - + + +
siCPT1A - - + -
Etomoxir - - - +
MEK
ERK
CT26
Cori.ST1911 - + + +
siCPT1A - - + -
Etomoxir - - - +
CPT1A
p-ERK
ERK
CT26
Cori.ST1911 - + + +
siCPT1A - - + -
Etomoxir - - - +
GAPDH
MEK
p-MEK

Fig5I

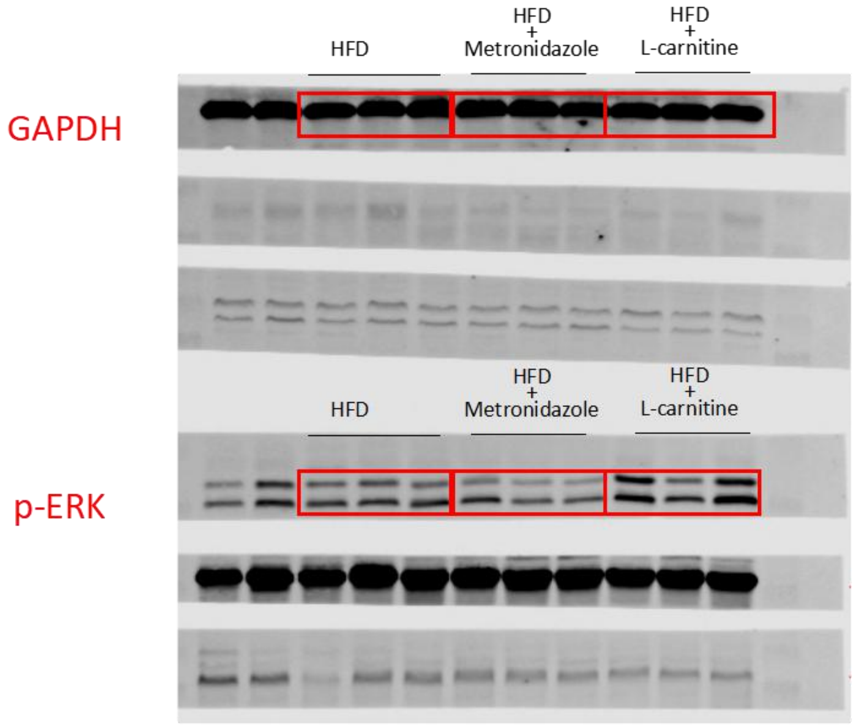
HFD
HFD + Metronidazole
HFD + L-carnitine
GAPDH
HFD
HFD + Metronidazole
HFD + L-carnitine
p-ERK

Fig6E

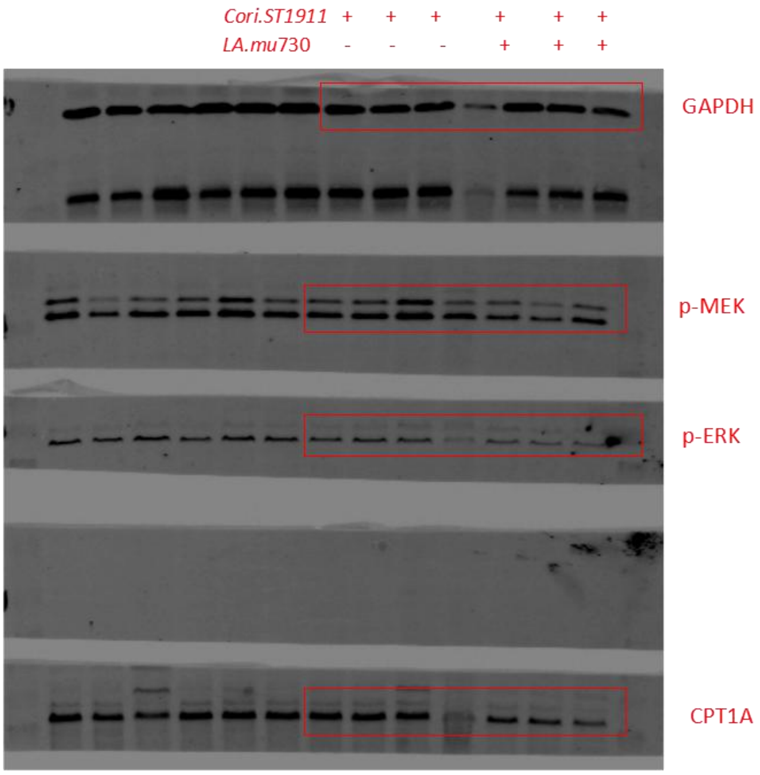
*Cori.ST1911* + + + + + +
*LA.mu730* - - - + + +
GAPDH
p-MEK
p-ERK
CPT1A
