# Supplementary material for: Endogenous Coriobacteriaceae enriched by a high-fat diet promotes colorectal tumorigenesis through the CPT1A-ERK axis
Source: NPJ Biofilms Microbiomes. 2024 Jan 20;10:5. doi: 10.1038/s41522-023-00472-7 (PMC10799938; doi:10.1038/s41522-023-00472-7)
Supplement: Supplementary file 3 — Reporting Summary [file 41522_2023_472_MOESM3_ESM.pdf]

nature portfolio

Corresponding author(s): Feng Bi

Last updated by author(s): Jul 3, 2023

Reporting Summary

Nature Portfolio wishes to improve the reproducibility of the work that we publish. This form provides structure for consistency and transparency in reporting. For further information on Nature Portfolio policies, see our Editorial Policies and the Editorial Policy Checklist.

Statistics

For all statistical analyses, confirm that the following items are present in the figure legend, table legend, main text, or Methods section.

n/a	Confirmed	
☐	☒	The exact sample size (*n*) for each experimental group/condition, given as a discrete number and unit of measurement
☐	☒	A statement on whether measurements were taken from distinct samples or whether the same sample was measured repeatedly
☐	☒	The statistical test(s) used AND whether they are one- or two-sided<br>*Only common tests should be described solely by name; describe more complex techniques in the Methods section.*
☒	☐	A description of all covariates tested
☒	☐	A description of any assumptions or corrections, such as tests of normality and adjustment for multiple comparisons
☐	☒	A full description of the statistical parameters including central tendency (e.g. means) or other basic estimates (e.g. regression coefficient) AND variation (e.g. standard deviation) or associated estimates of uncertainty (e.g. confidence intervals)
☒	☐	For null hypothesis testing, the test statistic (e.g. *F*, *t*, *r*) with confidence intervals, effect sizes, degrees of freedom and *P* value noted<br>*Give P values as exact values whenever suitable.*
☒	☐	For Bayesian analysis, information on the choice of priors and Markov chain Monte Carlo settings
☒	☐	For hierarchical and complex designs, identification of the appropriate level for tests and full reporting of outcomes
☒	☐	Estimates of effect sizes (e.g. Cohen's *d*, Pearson's *r*), indicating how they were calculated

Our web collection on statistics for biologists contains articles on many of the points above.

Software and code

Policy information about availability of computer code

Data collection	National Center for Biotechnology Information(NCBI)
Data analysis	Bio-Rad CFX Maestro 1.0(Version: 4.0.2325.0418)<br>GraphPad Prism 9.0(Version: 9.3.1)<br>MEGA 7(Version: 7.0.26)<br>Image J<br>Analyst (Version: 1.6.2)<br>Usearch(Version: 10.0)<br>Fastp (version 0.20.0)<br>usesrch10.0<br>FLASH (version 1.2.7)<br>Qiime(version 1.9.1)<br>Wtdbg2<br>SPSS Stastistic(Version: 17.0)

For manuscripts utilizing custom algorithms or software that are central to the research but not yet described in published literature, software must be made available to editors and reviewers. We strongly encourage code deposition in a community repository (e.g. GitHub). See the Nature Portfolio guidelines for submitting code & software for further information.

Data

Policy information about availability of data

All manuscripts must include a data availability statement. This statement should provide the following information, where applicable:

- Accession codes, unique identifiers, or web links for publicly available datasets
- A description of any restrictions on data availability
- For clinical datasets or third party data, please ensure that the statement adheres to our policy

All data generated in this manuscript are available within the paper and its Supplementary Methods.

Research involving human participants, their data, or biological material

Policy information about studies with human participants or human data. See also policy information about sex, gender (identity/presentation), and sexual orientation and race, ethnicity and racism.

Reporting on sex and gender	Not applicable
Reporting on race, ethnicity, or other socially relevant groupings	Not applicable
Population characteristics	Not applicable
Recruitment	Not applicable
Ethics oversight	Not applicable

Note that full information on the approval of the study protocol must also be provided in the manuscript.

Field-specific reporting

Please select the one below that is the best fit for your research. If you are not sure, read the appropriate sections before making your selection.

☒ Life sciences ☐ Behavioural & social sciences ☐ Ecological, evolutionary & environmental sciences

For a reference copy of the document with all sections, see nature.com/documents/nr-reporting-summary-flat.pdf

Life sciences study design

All studies must disclose on these points even when the disclosure is negative.

Sample size	Sample size was based on our previous publication(doi: 10.1186/s13046-021-01971-1) and according to the minimum experimental requirements.
Data exclusions	No data were excluded.
Replication	For animal experiments, 6-8 mice were included for each group; for LC-MS test and western blotting, sample replications were never less than 3.
Randomization	All the mice were randomly allocated into different groups.
Blinding	Blinding were not necessary because experimental conditions were well-controlled and results were quantitative.

Reporting for specific materials, systems and methods

We require information from authors about some types of materials, experimental systems and methods used in many studies. Here, indicate whether each material, system or method listed is relevant to your study. If you are not sure if a list item applies to your research, read the appropriate section before selecting a response.

Materials & experimental systems

n/a	Involved in the study
☐	☒ Antibodies
☐	☐ Eukaryotic cell lines
☒	☐ Palaeontology and archaeology
☐	☒ Animals and other organisms
☒	☐ Clinical data
☒	☐ Dual use research of concern
☒	☐ Plants

Methods

n/a	Involved in the study
☒	☐ ChIP-seq
☒	☐ Flow cytometry
☒	☐ MRI-based neuroimaging

Antibodies

Antibodies used: CPT1A (1:1,000, 15184-1-AP, Proteintech, China), p-ERK (1:2000, 201015, Abcam, USA), ERK (1:2000, ab184699, Abcam, USA), CPT2 (1:1000, 26555-1-AP, Proteintech, China), MEK (1:1000, ab178876, Abcam, USA), p-MEK (1:2000, 2338, Cell Signaling, USA), and GAPDH (1:2000, TA-08, ZSGB-BIO).;MUC2 (1:500, ab272692, Abcam, USA), ZO-1(1:500, ab221547, Abcam, USA), and occludin (1:500, ab216327, Abcam, USA),Ki67 (1:500, ab15580, Abcam, USA). Secondary antibodies included IRDye 800CW goat anti-rabbit IgG (H&L) (926-32211, Licor, USA), IRDye 680CW goat anti-mouse IgG (H&L) (926-68070, Licor, USA), and anti-HRP rabbit polyclonal antibody (D110011, Sangon Biotech, China), goat anti-rabbit IgG H&L (Alexa Fluor 488, 1:5000, ab150077, Abcam, USA).

Validation: All antibodies will be pilot tested according to the manufacturer's instructions to ensure that the antibodies are worked.

Eukaryotic cell lines

Policy information about cell lines and Sex and Gender in Research

Cell line source(s): Murine colon cancer cell line CT26 were purchased from the American Type Culture Collection (ATCC); cell line MC38 are stored in our laboratory.

Authentication: Murine colon cancer cell line CT26 Cell lines were purchased from the American Type Culture Collection (ATCC) and no other authentication method was performed; murine colon cancer cell line MC38 have been performed Authentication Service by Yaji Biological Company.

Mycoplasma contamination: All cell lines were tested to exclude mycoplasma contamination.

Commonly misidentified lines (See ICLAC register): No commonly misidentified cell lines were used.

Animals and other research organisms

Policy information about studies involving animals; ARRIVE guidelines recommended for reporting animal research, and Sex and Gender in Research

Laboratory animals: Male C57BL/6J and female BALB/c mice (4-5 weeks) were purchased from Beijing HFK Bioscience. C57BL6/J-ApcMin (ApcMin/+) mice were obtained from the Model Animal Research Center of Nanjing University

Wild animals: Not applicable

Reporting on sex: Not applicable

Field-collected samples: All animals were kept in specific-pathogen free miniature isolation cages. All experiments were performed in accordance with relevant guidelines and regulations. Using C57BL/6J and ApcMin/+ male mice to keep mice alive during the establishment of a model of primary colon cancer; female BALB/c mice were used in the establishment of subcutaneous tumor transplantation model to avoid skin ulceration caused by fighting.

Ethics oversight: The animal experiment were approved by the Institutional Animal Care and Animal Ethics Committee of Sichuan University (No.20211262A)

Note that full information on the approval of the study protocol must also be provided in the manuscript.
